# Expansion of the Catalytic Repertoire of Alcohol Dehydrogenases in Plant Metabolism**

**DOI:** 10.1002/anie.202210934

**Published:** 2022-10-26

**Authors:** Chloe Langley, Evangelos Tatsis, Benke Hong, Yoko Nakamura, Christian Paetz, Clare E. M. Stevenson, Jerome Basquin, David M. Lawson, Lorenzo Caputi, Sarah E. O'Connor

**Affiliations:** ^1^ Department of Natural Product Biosynthesis Max Planck Institute for Chemical Ecology Hans-Knöll Straße 8 Jena 07745 Germany; ^2^ Chinese Academy of Sciences Centre for Excellence in Molecular Plant Sciences Shanghai Institute of Plant Physiology and Ecology 300 Feng Lin Road Shanghai 200032 China; ^3^ Research Group Biosynthesis and NMR Max Planck Institute for Chemical Ecology Hans-Knöll Straße 8 Jena 07745 Germany; ^4^ Department of Biochemistry and Metabolism John Innes Centre Norwich Research Park Norwich NR4 7UH UK; ^5^ Department of Structural Cell Biology Max-Planck Institute for Biochemistry Am Klopferspitz 18, Martinsried 82152 Planegg Germany

**Keywords:** Alcohol Dehydrogenases, Biosynthesis, Enzymes, Natural Products, Oxidoreductases

## Abstract

Medium‐chain alcohol dehydrogenases (ADHs) comprise a highly conserved enzyme family that catalyse the reversible reduction of aldehydes. However, recent discoveries in plant natural product biosynthesis suggest that the catalytic repertoire of ADHs has been expanded. Here we report the crystal structure of dihydroprecondylocarpine acetate synthase (DPAS), an ADH that catalyses the non‐canonical 1,4‐reduction of an α,β‐unsaturated iminium moiety. Comparison with structures of plant‐derived ADHs suggest the 1,4‐iminium reduction does not require a proton relay or the presence of a catalytic zinc ion in contrast to canonical 1,2‐aldehyde reducing ADHs that require the catalytic zinc and a proton relay. Furthermore, ADHs that catalysed 1,2‐iminium reduction required the presence of the catalytic zinc and the loss of the proton relay. This suggests how the ADH active site can be modified to perform atypical carbonyl reductions, providing insight into how chemical reactions are diversified in plant metabolism.

## Graphical abstract

Medium chain alcohol dehydrogenases usually perform a reversible 1,2‐reduction of aldehydes to form the corresponding alcohols. Here we show how the active site of these dehydrogenases can be modified to perform 1,2‐reduction of iminium moieties, as well 1,4‐reduction of α,β‐unsaturated iminium groups and 1,4‐reduction of α,β‐unsaturated aldehydes.

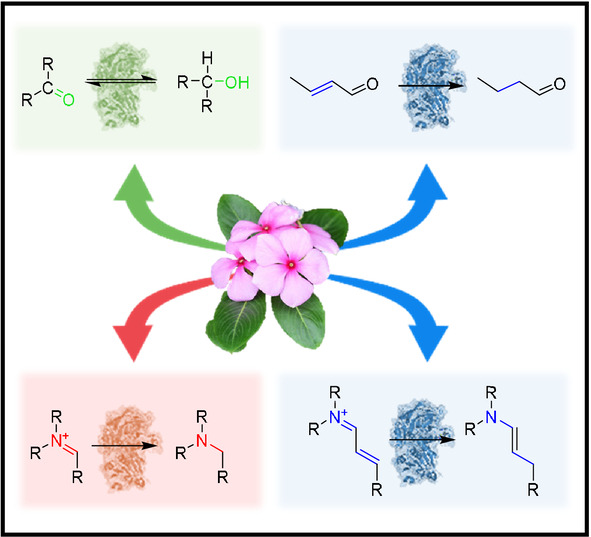

Alcohol dehydrogenases (ADHs EC 1.1.1.1) are NAD(P)H‐dependent medium‐chain oxidoreductases found in all kingdoms of life. These enzymes typically catalyse the reversible reduction of aldehydes or ketones to the corresponding alcohol (Figure 1A).[^1^, ^2^, ^3^, ^4^] The structural motifs of ADHs are highly conserved in all known eukaryotic examples; most notably, a zinc ion involved in catalysis, a second zinc ion involved in maintaining protein structure, and the Rossmann peptide‐fold involved in cofactor binding (Figures S1). ADHs have been shown to catalyse many complex biochemical transformations in plant natural product biosynthesis, suggesting that their catalytic repertoire has been expanded. For example, we recently reported the discovery of dihydroprecondylocarpine acetate synthase (DPAS), an ADH involved in vinblastine biosynthesis in the plant *Catharanthus roseus* ^[5]^ and in ibogaine biosynthesis in the phylogenetically related species *Tabernanthe iboga*.^[6]^ Since the product of DPAS is unstable and either immediately decomposes or rearranges in the presence of a downstream cyclase enzyme, the reaction remained unsubstantiated (Figure 1B). However, the cyclised products suggest that DPAS catalyses the 1,4‐reduction of an α,β‐unsaturated iminium which is an hitherto unprecedented reaction for an ADH. Here, we use isotopic labelling to definitively establish that DPAS catalyses this unusual 1,4‐reduction. We report four crystal structures of apo‐ and substrate‐bound DPAS from two phylogenetically related species. These structures reveal, surprisingly, the loss of the catalytic zinc ion from the DPAS active site, indicating that zinc is not strictly required for reduction by ADHs. We also report the structure of the ADH geissoschizine synthase (GS) that catalyses an atypical 1,2‐iminium reduction. Comparison of the active site of DPAS and GS with other highly similar ADHs that catalyse either 1,2‐aldehyde reduction or 1,2‐reduction of an iminium moiety suggests that changes in the proton relay system are also implicated in modulating ADH reactivity. The mechanism and structure of DPAS highlights the catalytic versatility of ADHs. Overall, these findings demonstrate how the active site of ADHs have been modulated to expand the catalytic scope of this large class of enzymes.

**Figure 1:**
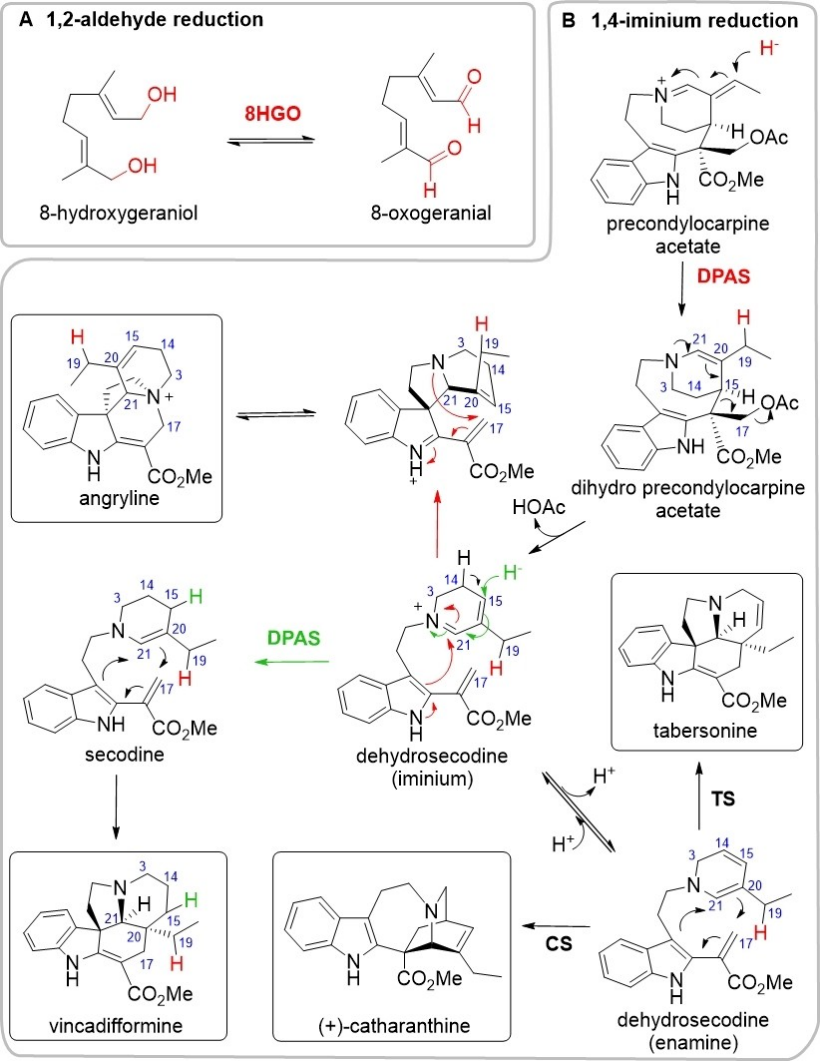
ADH catalysed reduction. A) Reversible oxidation/reduction reaction of 8‐hydroxygeraniol/8‐oxogeranial catalysed by canonical ADH 8HGO. B) Proposed 1,4‐reduction of precondylocarpine acetate catalysed by *Cr*DPAS leading to dehydrosecodine intermediate. Dehydrosecodine can undergo a further 1,4‐reduction catalysed by *Cr*DPAS to form vincadifformine, rearrangement in acidic conditions to form angryline, or cyclisation reaction catalysed by various cyclase enzymes such as tabersonine synthase (TS) or catharanthine synthase (CS). See Figure S2 for MS/MS of isotopic labelled angryline and vincadifformine.

The recently discovered DPAS reduces the substrate precondylocarpine acetate to yield an initial, unstable product, which is hypothesised to be dihydroprecondylocarpine acetate. Dihydroprecondylocarpine acetate is proposed to spontaneously desacetoxylate, likely forming dehydrosecodine, which either decomposes or can serve as a substrate for several cyclase enzymes such as catharanthine synthase (CS) or tabersonine synthase (TS) to form distinct alkaloid scaffolds (Figure 1B).[^5^, ^6^, ^7^, ^8^] We proposed the reduction of precondylocarpine acetate proceeds through an irreversible 1,4‐α,β‐unsaturated iminium reduction at C19, though this could only be inferred due to the extensive rearrangement of the reduced product. Although dihydroprecondylocarpine acetate or dehydrosecodine cannot be isolated, a trapped form of the desacetoxylated product, angyline, is sufficiently stable under acidic conditions to be characterised (Figure 1B).^[5]^ Therefore, to definitively establish the regioselectivity of the DPAS reduction, we performed the enzymatic reaction in the presence of deuterium‐labelled coenzyme, 4‐pro‐*R*‐NADPD (Figure S2). The isolated deuterated angryline was characterised by NMR which clearly showed deuterium incorporation at C19 (Figure S3). The site of incorporation indicates DPAS catalyses the 1,4‐reduction of the α,β‐unsaturated iminium moiety and is thereby the first reported ADH with 1,4‐reduction activity (Figure S2).

Although previous reports have indicated that a dedicated cyclase enzyme is required to form the product vincadifformine,^[9]^ we observed product formation when only DPAS and NADPH is incubated with precondylocarpine acetate (Figure S4). This suggests after DPAS initially reduces precondylocarpine acetate to form dehydrosecodine, this product can be reduced a second time by DPAS to form secodine, which spontaneously cyclises to vincadifformine (Figure 1B). Isolation and characterisation of the deuterated vincadifformine product indicate DPAS catalyses a second non‐stereoselective 1,4‐reduction reaction at C15 of dehydrosecodine (Figure S2, Figure S5–11). We hypothesise that the free rotation of dehydrosecodine allows multiple binding orientations within the active site, resulting in non‐stereoselective reduction (Figure 1B). Moreover, CD measurement of the isolated vincadifformine suggest the product is racemic, consistent with a non‐enzymatic cyclisation (Figure S11).

Having established DPAS catalyses a 1,4‐iminium reduction reaction, we solved the crystal structure of this enzyme to understand the structural basis for the switch in catalytic activity compared to canonical 1,2‐aldehyde reducing ADHs. The partial structure of DPAS from the vinblastine producing plant *C. roseus* (*Cr*DPAS, PDB 8B26, 2.16 Å, Figure S12, Table S3), as well as the full structure of the apo‐ and substrate‐bound DPAS from the phylogenetically related alkaloid producing plant *T. iboga* (*Ti*DPAS2, 86 % amino acid identity to *Cr*DPAS, 2.42 Å apo‐ PDB 8B26, 1.88 Å precondylocarpine acetate‐bound PDB 8B1V, and 2.24 Å stemmadenine acetate‐bound PDB 8B25, Figure 2A and C, Figure S13, Table S4–S6) were solved by X‐ray crystallography. Despite the presence of NADP+ in DPAS crystallisation conditions, none of the solved structures had sufficient density to model the cofactor. Therefore, to assess the position of the bound substrate relative to the cofactor, NADPH was docked into the active site of DPAS co‐crystallised with the substrate precondylocarpine acetate. The 4‐pro‐*R* hydride of NADPH was measured to be 3.7 Å from C19 of the substrate which is consistent with hydride addition at this carbon (Figure 2C). Additionally, the 4‐pro‐*R* hydride was 4.6 Å from C15, which is the site of the second reduction to form secodine (Figure S13), although we note that dehydrosecodine may bind in a different orientation within the substrate cavity.

**Figure 2:**
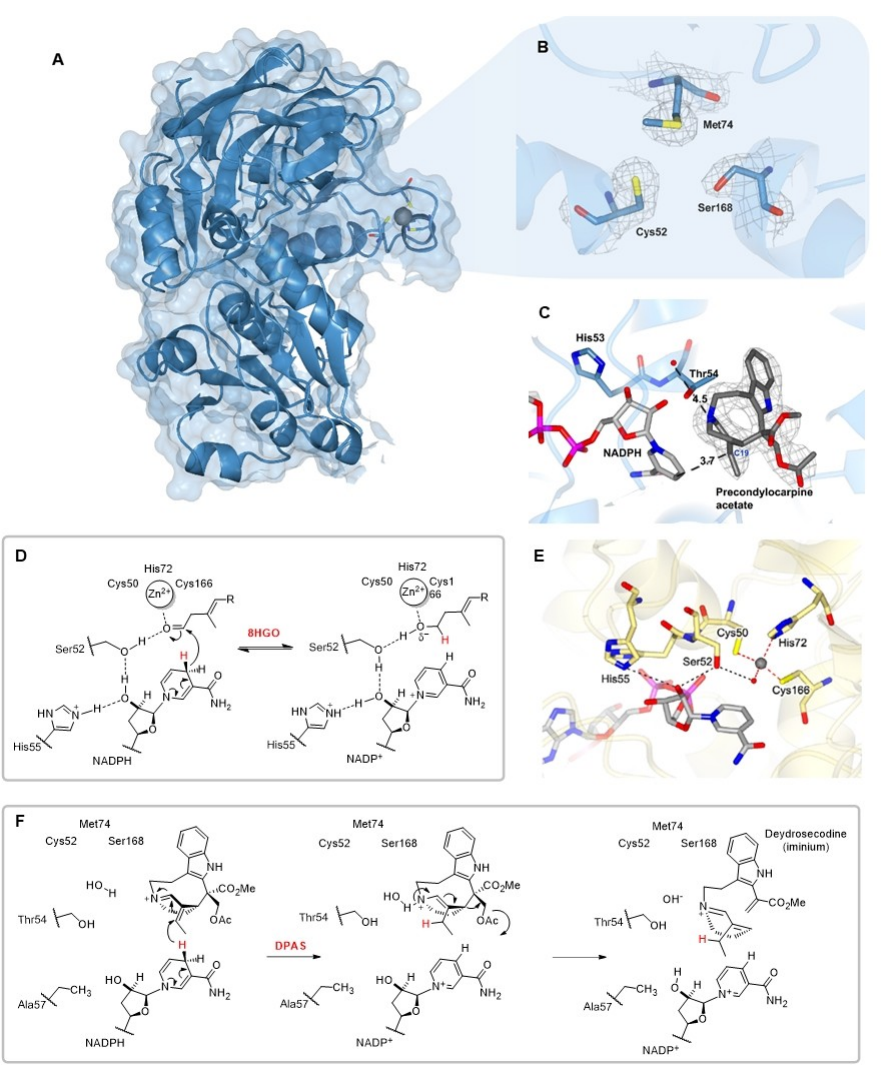
Comparison of DPAS with canonical ADH 8HGO. A) Structure of *Ti*DPAS2 structure showing coordination of the structural zinc. B) Electron density of *Ti*DPAS2 active site showing lack of catalytic zinc. C) Active site of *Ti*DPAS2 showing bound precondylocarpine acetate with docked NADPH. D) Proposed mechanism of *Cr*8HGO aldehyde reduction. E) Active site of *Cr*8HGO with NADP^+^ showing residues involved in catalysis. F) Proposed mechanism of 1,4‐ reduction of an α,β‐unsaturated iminium catalysed by DPAS.

To establish a point of comparison for DPAS, we used the previously reported structure of a canonical ADH from the vinblastine pathway of *C. roseus*, 8HGO, which catalyses the reversible oxidation/reduction of 8‐hydroxygeraniol/8‐hydroxygeranial (PDB 6K3G, 60.67 % amino acid identity to DPAS, Figure 2D).^[10]^ The active site of 8HGO contains all of the highly conserved residues involved in ADH catalysis (Figure 2E, Figure S1). Specifically, the catalytic zinc is coordinated by two cysteine thiol groups (Cys50, Cys166), a histidine side chain (His72) and a water molecule.[^4^, ^11^] The structural zinc—which is distal to the substrate binding pocket and maintains protein domain structure—is tetrahedrally coordinated by four thiol groups (Cys98, Cys106, Cys109, and Cys117). In the canonical ADH reduction mechanism, the aldehyde of the substrate is thought to bind to the catalytic zinc, displacing the water molecule, and the hydride group is transferred from the cofactor to the electrophilic carbon of the aldehyde moiety. The catalytic zinc likely acts as a Lewis acid during catalysis, stabilizing the alkoxide intermediate that forms during the reduction. The reduction is also facilitated by a proton relay system composed of a hydrogen‐bonding network between a histidine side chain (His55 in 8HGO), the 2′O ribose of the cofactor, a hydroxyl‐group (typically a serine or threonine side chain, Ser52 in 8HGO) and the aldehyde of the substrate (Figure 2D).^[12]^ The residues involved in binding the catalytic zinc and the proton relay have been demonstrated to be essential for catalysis in numerous studies of ADHs.[^2^, ^3^, ^12^, ^13^]

Although electron density for the structural zinc ion was clearly observed in all *Ti*DPAS2 structures (the *Cr*DPAS structure lacked electron density for this region), surprisingly, the substrate pocket lacked electron density at the expected site of the catalytic zinc (Figure 2B, Figure S12). This has previously only been reported in a prokaryote ADH.^[14]^ The lack of the catalytic zinc in DPAS is substantiated by the observation that two of the conserved catalytic zinc coordinating residues are mutated (His74Met and Cys168Ser, Figure S1). The zinc ion plays a crucial catalytic role as a Lewis acid in aldehyde reduction. However, the lack of negatively charged intermediate in the 1,4‐reduction of an unsaturated iminium system may obviate the need for a Lewis acid.

To investigate the catalytic mechanism of *Cr*DPAS, we performed site directed mutagenesis of key residues involved in coordination of the catalytic zinc and the proton relay (Figure S14). A DPAS mutant in which the zinc ion coordinating residues were restored (Met74His, Ser168Cys) still reduced precondylocarpine acetate (Figure S15). However, the Met74His mutant had decreased levels of the over reduced vincadifformine product. In contrast, the Ser168Cys mutant showed increased vincadifformine production. We speculate removal of the catalytic zinc in DPAS enables a larger substrate pocket to better accommodate the substrate. Computational estimation found *Ti*DPAS2 had a larger substrate cavity than other structures of ADHs involved in monoterpene indole alkaloid (MIA) biosynthesis (Figure S16). The change in the ratio between the single (angryline) and the double (vincadifformine) reduced product in these mutants could be due to modulating the shape of the binding pocket and the manner in which the substrates bind. Due to the instability of the substrate and products, accurate product quantification required for steady‐state kinetics was impractical, so only qualitative conclusions were drawn from these mutational analyses.

Canonical ADHs rely on a proton relay enabled by conserved Ser/Thr and His residues (Figure 2D). The hydroxyl moiety of Thr54 in TiDPAS (corresponding to Ser52 in 8HGO) was 3.6 Å from the nitrogen of the substrate's iminium suggesting this residue may act in the proton relay (Figure 2C). However, while enzyme activity was abolished in a Thr54Phe mutant, the Thr54Ala mutant was active (Figure S15). Therefore, while the size of this residue may impact substrate binding, a proton relay involving a hydroxyl group is not essential for catalysis. The γN of a highly conserved histidine which typically acts as a proton donor during catalysis (His55 in 8HGO, Figure 2D), is mutated to Ala57 in *Cr*DPAS and Thr57 in *Ti*DPAS2. We speculated a nearby histidine (His53) may be acting as a proton donor, however His53Ala mutation did not affect DPAS activity, indicating this residue is not essential for catalysis of the 1,4‐reduction reaction (Figure S15). Collectively, these findings suggest the 1,4‐α,β‐unsaturated iminium reduction catalysed by *Cr*DPAS does not require a catalytic zinc or a proton relay, but reintroduction of these features did not abolish activity (Figure 2F). The inherent reactivity of the unsaturated iminium substrate compared to aldehyde substrates means that the enzyme may not require a Lewis acid to stabilize the reaction as the reduction proceeds. A number of ordered water molecules in the DPAS structure could take over the role of proton donor (Figure 2C). Correct positioning of the substrate relative to the NADPH cofactor may be sufficient for reduction of this already activated substrate.

In addition to the 1,4‐reduction catalysed by DPAS, one additional non‐canonical reduction reaction has been reported for eukaryotic ADHs: the 1,2‐reduction of an iminium moiety. GS catalyses the irreversible 1,2‐iminium reduction of strictosidine aglycone to form geissoschizine, another intermediate in vinblastine biosynthesis (Figure 3A).[^8^, ^15^] Additionally, tetrahydroalstonine synthase (THAS) is an 1,2‐iminium reductase that reduces an alternate isomer of strictosidine aglycone to generate the alkaloid tetrahydroalstonine (Figure 3A).[^16^, ^17^] The apo‐ and holo structures of THAS were previously solved (PDB accessions 5FI3 and 5H83), but the mechanistic basis behind the switch in activity from aldehyde to iminium reduction remains poorly understood. In light of the insights from the DPAS structure, we solved the holo structure of GS to 2.00 Å resolution (PDB 8A3N, Figure S17, Table S7) and compared the THAS and GS structures with the 1,4‐reduction mechanism of precondylocarpine acetate.

**Figure 3:**
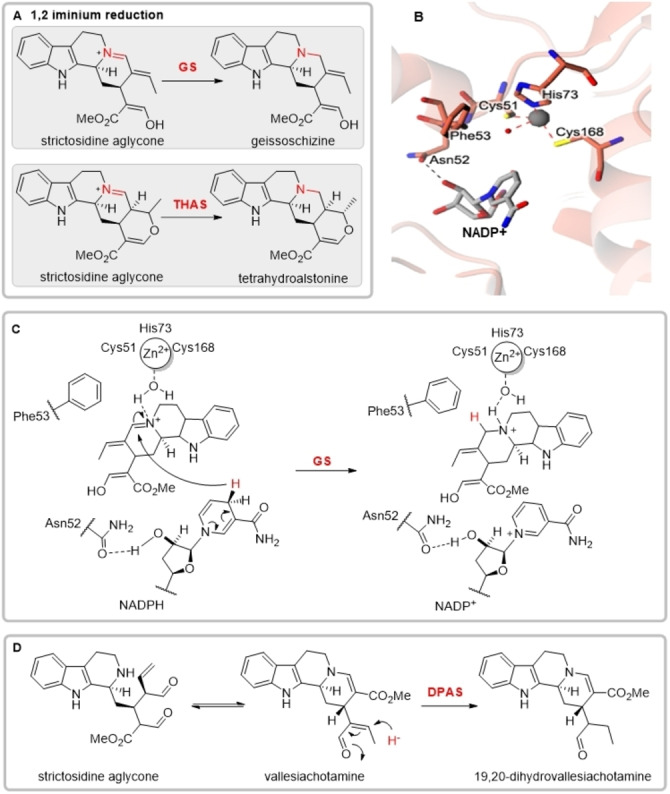
Mechanism of strictosidine aglycone reduction. A) 1,2‐iminium reductions of strictosidine aglycone catalysed by ADHs GS and THAS. B) Active site of GS co‐crystallised with NADP^+^ showing residues involved in coordination of the catalytic zinc and loss of the proton relay. C) Proposed mechanism of 1,2‐iminium reduction catalysed by GS. D) Proposed mechanism of 1,4‐aldehyde reduction of strictosidine aglycone catalysed by DPAS.

GS and THAS (47.7 % and 53.8 % amino acid identity to DPAS respectively) each crystallised with both the catalytic and structural zinc ions bound. Mutagenesis of GS residues involved in the coordination of the catalytic zinc (His73 and Cys168) resulted in the abolishment of GS activity, suggesting that, as in the canonical ADH mechanism, the catalytic zinc is essential for 1,2‐reduction of the iminium (Figure S18). Additionally, GS and THAS each had mutations in highly conserved residues involved in the proton relay observed in canonical ADH reduction (Figure 3B, Figure S1). THAS was previously postulated to use Tyr56 (corresponding to Ser52 in 8HGO) in the proton relay^[16]^ but careful inspection of the THAS structure in the context of this study suggests the hydroxyl group may not be appropriately positioned for catalysis. Moreover, GS contains a Phe residue in this position (Phe53). No substantial change in reduction activity was observed for GS Phe53Tyr mutant or the corresponding THAS Tyr56Phe mutant, suggesting the hydroxyl‐group has no catalytic role (Figure S19). GS and THAS also both lack the highly conserved histidine residue involved in the proton relay (His55 in 8HGO replaced with Glu56 in GS and Glu59 in THAS). Although THAS Glu59 is positioned 3.6 Å from the 2′O of bound‐NADP^+^, GS Glu56 is positioned 7.6 Å from this moiety which is not consistent with a role for this residue in cofactor binding or catalysis (Figure 3B). This residue has been previously reported to affect the stereoselectivity of the reduction in THAS, likely due to the orientation of the cofactor in the substrate pocket.^[16]^ These findings indicate the proton relay present in canonical ADHs is not required for 1,2‐iminium reduction, possibly due to iminium moieties typically being more activated than aldehydes in the context of reduction. We hypothesise the water molecule in the fourth coordination position of the catalytic zinc remains bound upon substrate binding. This water could hydrogen bond to the substrate iminium, serving as the proton donor, with the zinc ion acting as a Lewis base (Figure 3C, Figure S17).

GS has been reported to be able to reduce precondylocarpine acetate, albeit it at substantially reduced efficiency compared to DPAS (Figure S20).^[8]^ This suggests 1,2‐iminium reducing ADHs can also catalyse a 1,4‐reduction provided the substrate can productively bind in the active site. Conversely, assay of DPAS with strictosidine aglycone, the substrate of GS and THAS, showed that DPAS could also turnover this substrate (Figure S21). However, NMR characterisation of the major product revealed a compound consistent with the structure of 19,20‐dihydrovallesiachotamine, which had only been previously partially characterised (Figure S22–S40).^[18]^ This structure indicates DPAS catalyses the 1,4‐reduction of the α,β‐unsaturated carbonyl in strictosidine aglycone at C19 (Figure 3D, Figure S13). Assay of strictosidine aglycone with DPAS mutants showed removal of the hydroxyl moiety of Thr54—either by mutating to Phe or Ala—abolished enzyme activity. This suggests DPAS Thr54 has a catalytic role in the presence of less electronically activated substrate strictosidine aglycone. Additionally, mutation of the residues corresponding to catalytic zinc binding and proton relay in DPAS resulted in either loss of activity with strictosidine aglycone, or 1,2‐iminium reduction of an alternative rearrangement of strictosidine aglycone to form tetrahydroalstonine (Figure S41). Mutation of these residues likely impact substrate binding, highlighting the plasticity of the active site of this enzyme. Increased substrate promiscuity within the ADH active site may be observed in these cases since certain catalytic requirements—zinc ion and proton relay—are not strictly required for the reduction of these substrates.

Phylogenetic analysis of previously characterised plant ADHs revealed three clades that grouped according to conservation of residues involved in the coordination of the catalytic zinc, and residues involved in proton relay and cofactor coordination (Figure 4A). Interestingly, we found the predicted catalytic activity based on residues involved in the coordination of the catalytic zinc and proton relay discussed in this study largely correlated with the experimentally verified activities of these enzymes (Figure S42). The phylogenetic distribution of these atypical ADHs is limited to the Apocynaceae, Loganiaceae and Rubiaceae families, plant families known to produce MIAs. However, this distribution may increase as more enzymes involved in plant secondary metabolism are discovered. The identification of sequence motifs may allow better predictions of enzyme function and mechanism. For example, the previously reported vomilenine reductase 2 (VR2) from *Rauwolfia* (a member of the Apocynaceae family) is in the same phylogenetic clade as DPAS (66 % amino acid identity) and is also missing the conserved residues important in coordinating the catalytic zinc (Phe73 instead of His and Asn167 instead of Cys) and the proton relay (Gln56 instead of His).^[19]^ After inspection of the substrate and product of VR2, we propose a mechanism for VR2 based on the mechanism of DPAS that involves a 1,4 unsaturated aldehyde reduction at C19 (Figure 4B).

**Figure 4:**
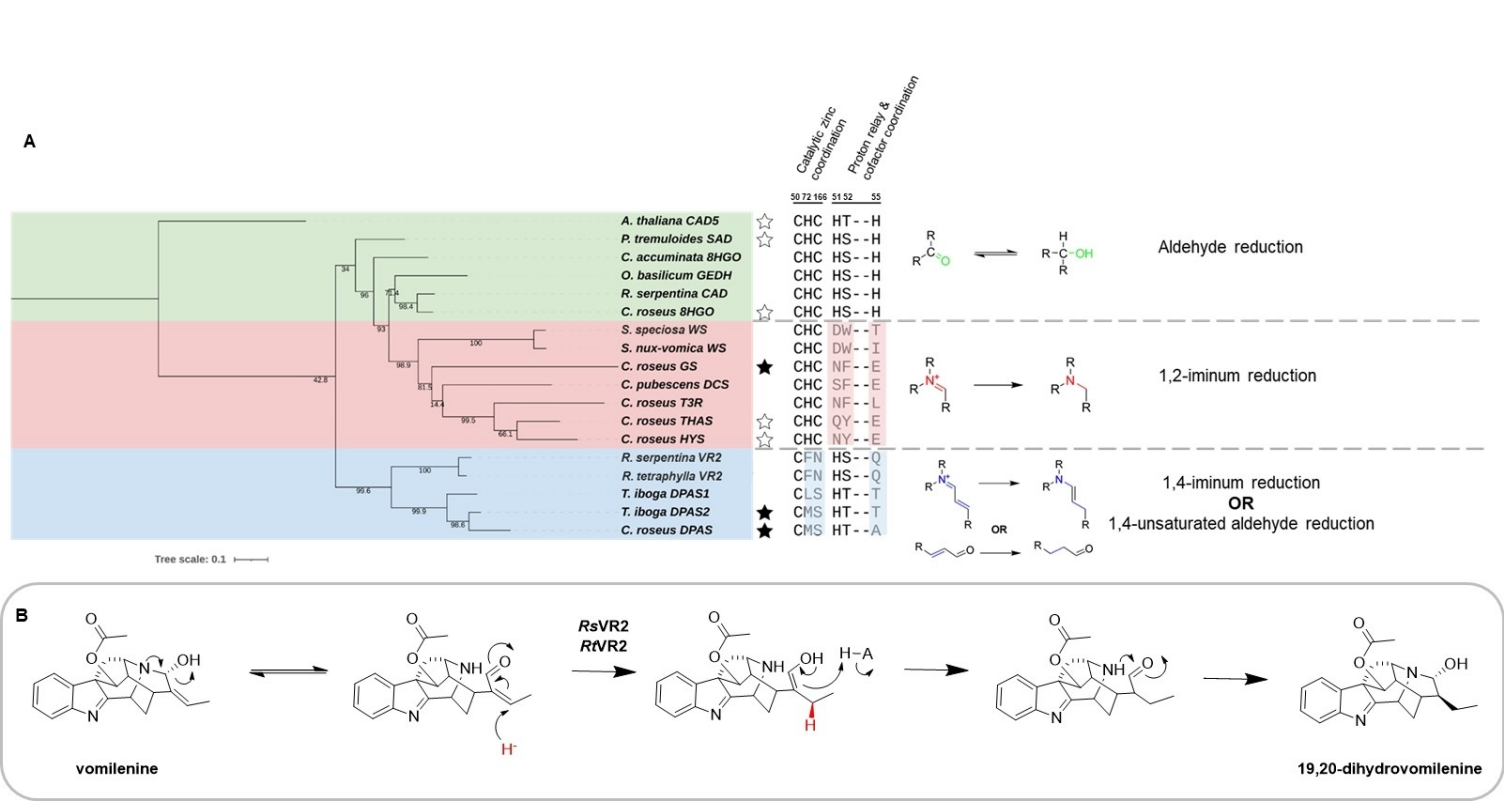
Phylogenetic distribution of non‐canonical ADHs in plantae. A) Tree of maximum likelihood of previously characterised plant ADHs that perform atypical reductions and conservation of residues coordinating the catalytic zinc and involved in the proton relay. Residue numbering based on *Cr*8HGO structure, stars indicate proteins with structure solved in either previous (line) or this study (filled). See Table S8 for sequences used. B) Proposed mechanism of *Rauwolfia* VR2 1,4 unsaturated aldehyde reduction of vomilenine.

ADHs are an ancient and widespread protein family that until recently had a limited reported catalytic repertoire. Here we show the structural basis of how ADHs can catalyse two distinct variations on carbonyl reduction. These enzymes make a valuable addition to other known 1,2‐imine reductases (IREDs).^[20]^ Furthermore, these findings demonstrate how ADH function has been co‐opted to perform atypical chemistry by mutations in key catalytic residues, enabling various biotechnological and gene discovery applications, and sheds light on the evolution of chemical diversity in plants.

## Conflict of interest

The authors declare no conflict of interest.

## Supporting information

As a service to our authors and readers, this journal provides supporting information supplied by the authors. Such materials are peer reviewed and may be re‐organized for online delivery, but are not copy‐edited or typeset. Technical support issues arising from supporting information (other than missing files) should be addressed to the authors.

Supporting Information

## Data Availability

The data that support the findings of this study are available in the Supporting Information of this article.
