# Supplementary material for: Expansion of the Catalytic Repertoire of Alcohol Dehydrogenases in Plant Metabolism
Source: Angew Chem Int Ed Engl. 2022 Oct 26;61(48):e202210934. doi: 10.1002/anie.202210934 (PMC9828224; doi:10.1002/anie.202210934)
Supplement: Supplementary file 1 — Supporting Information [file ANIE-61-0-s001.pdf]

Angewandte Chemie
Eine Zeitschrift der Gesellschaft Deutscher Chemiker

Supporting Information

Expansion of the Catalytic Repertoire of Alcohol Dehydrogenases in Plant Metabolism

C. Langley, E. Tatsis, B. Hong, Y. Nakamura, C. Paetz, C. E. M. Stevenson, J. Basquin, D. M. Lawson, L. Caputi, S. E. O'Connor**

Table of Contents

Experimental Procedures....4
Chemicals and molecular biology reagents....4
Cloning and mutagenesis....4
Expression and purification of proteins in *E. coli*....4
*Cr*PAS insect cell expression....4
*Cr*DPAS, *Cr*GS and *Cr*THAS small-scale protein expression and purification....4
*Cr*DPAS, *Ti*DPAS2, *Cr*GS, *Cr*SGD, *Cr*PAS and *Tb*ADH large-scale purification....5
Synthesis of NADPD....5
In vitro enzyme assays....5
UPLC-MS....5
Production and isolation of *d*-angryline and d_2-vincadifformine....6
Production and isolation of 19,20-dihydrovallesiachotamine....6
NMR of *d*-angryline, *d*-vincadifformine and 19,20-dehydrovallesiachotamine....6
ECD measurement....6
ECD spectral calculations for (-)-vincadifformine....7
Protein crystallisation....7
X-ray data collection, processing and structure solution....7
Docking simulations....8
Phylogenetic analysis....8
Supporting Figures....9
Figure S1. MUSCLE amino acid sequence alignment of ADHs highlighting key residues....9
Figure S2. Deuterium labelling of DPAS reduction of precondylocarpine acetate....10
Figure S3. Comparison of ^{1}H NMR data for angryline and *d*-angryline showing position of deuterium incorporation....12
Figure S4. LC-MS chromatograms showing formation of angryline and vincadifformine with *Catharanthus roseus* ADHs....13
Figure S5. ^{1}H NMR data for (-)-vincadifformine in MeOH-d_3....14
Figure S6. ^{1}H NMR data for *m/z* 339, (–)-vincadifformine (standard). Phase sensitive HSQC, full range in MeOH-d_3....15
Figure S7. ^{1}H NMR data for *m/z* 339, (–)-vincadifformine (standard). Phase sensitive HSQC, aliphatic range in MeOH-d3....16
Figure S8. ^{1}H NMR data for d_2-(±)-vincadifformine in MeOH-d_3....17
Figure S9. Phase sensitive HSQC NMR data for *m/z* 341, d_2-(±)-vincadifformine full range in MeOH-d_3....18
Figure S10. Phase sensitive HSQC NMR data for *m/z* 341, d_2-(±)-vincadifformine, aliphatic range in MeOH-d_3....19
Figure S11. Comparison of the experimental ECD spectra of d_2-vincadifformine (green), vincadifformine standard (black) and calculated ECD spectra of (-)-vincadifformine (red)....20
Figure S12. Structure of *Catharanthus roseus* DPAS (*Cr*DPAS) crystallised as a homodimer at 2.45 Å resolution....21
Figure S13. Structure of *Tabernanthe iboga* DPAS2 (*Ti*DPAS2)....22
Figure S14. Catharanthus DPAS (*Cr*DPAS) active site with highly conserved residues involved in canonical ADH enzymes with the coordination of the catalytic zinc (Met74 Ser168) and the proton relay (His53, Thr54) that were mutated in this study....23
Figure S15. Wild-type *Cr*DPAS and mutants incubated with precondylocarpine acetate showing production of angryline and vincadifformine....24
Figure S16. Substrate cavity volume and area of *Cr*8HGO, *Cr*GS, *Cr*THAS and *Ti*DPAS2....25
Figure S17. A. *Catharanthus roseus* GS (*Cr*GS) crystallised as a homodimer bound to cofactor $NADP^+$ at 2.00 Å....26
Figure S18. LC-MS chromatograms of *Cr*GS and mutants reacted with substrate strictosidine aglycone and cofactor NADPH....27
Figure S19. LC-MS chromatograms of *Cr*GS and *Cr*GS Phe53Tyr mutant and the corresponding *Cr*THAS and *Cr*THAS Tyr56Phe mutant reacted with substrate strictosidine aglycone and cofactor NADPH....28
Figure S20. LC-MS chromatograms of *Cr*DPAS and CrGS reacted with substrate precondylocarpine acetate and cofactor NADPH incubated for 1 hour at 30 °C....29
Figure S21. LC-MS chromatograms of *Cr*DPAS, *Ti*DPAS1 and *Ti*DPAS2 reacted with substrate strictosidine aglycone and cofactor NADPH....30
Figure S22. MS/MS and NMR data of 19,20-dihydrovallesiachotamine....31
Figure S23. ^{1}H NMR data of 19,20-dihydrovallesiachotamine with water suppression, full range in MeOH-d_3....32

Figure S24. ^{1}H NMR data of 19,20-dihydrovallesiachotamine with water suppression, aldehyde range in MeOH-*d_3* 33
Figure S25. ^{1}H NMR data of 19,20-dihydrovallesiachotamine with water suppression, aromatic range in MeOH-*d_3*.. 34
Figure S26. ^{1}H NMR data of 19,20-dihydrovallesiachotamine with water suppression, aliphatic range in MeOH-*d_3*. 35
Figure S27. NMR data of 19,20-dihydrovallesiachotamine, phase sensitive HSQC, full range in MeOH-*d_3*. 36
Figure S28. NMR data of 19,20-dihydrovallesiachotamine, phase sensitive HSQC, aldehyde and aromatic range in MeOH-*d_3*. 37
Figure S29. NMR data of 19,20-dihydrovallesiachotamine, phase sensitive HSQC, aliphatic range in MeOH-*d_3*. 38
Figure S30. NMR data of 19,20-dihydrovallesiachotamine, HMBC, full range in MeOH-*d_3*. 39
Figure S31. NMR data of 19,20-dihydrovallesiachotamine, HMBC, aldehyde and aromatic range in MeOH-*d_3*. 40
Figure S32. NMR data of 19,20-dihydrovallesiachotamine, HMBC, aliphatic range in MeOH-*d_3*.. 41
Figure S33. NMR data of 19,20-dihydrovallesiachotamine, DEPTQ, power spectrum, full range in MeOH-*d_3*. 42
Figure S34. NMR data of 19,20-dihydrovallesiachotamine, 1H-1H DQF COSY with water suppression, magnitude mode processed, full range in MeOH-*d_3*. 43
Figure S35. NMR data of 19,20-dihydrovallesiachotamine, 1H-1H DQF COSY with water suppression, magnitude mode processed, aldehyde and aromatic range in MeOH-*d_3*. 44
Figure S36. NMR data of 19,20-dihydrovallesiachotamine, 1H-1H DQF COSY with water suppression, magnitude mode processed, aliphatic range in MeOH-*d_3*. 45
Figure S37. NMR data of 19,20-dihydrovallesiachotamine, ^{1}H-^{1}H ROESY with water suppression, full range in MeOH-*d_3* 46
Figure S38. NMR data of 19,20-dihydrovallesiachotamine, ^{1}H-^{1}H ROESY with water suppression, aldehyde and aromatic range in MeOH-*d_3* 47
Figure S39. NMR data of 19,20-dihydrovallesiachotamine, ^{1}H-^{1}H ROESY with water suppression, aliphatic range in MeOH-*d_3*. 48
Figure S40. Structure of 19,20-dihydrovallesiachotamine optimized using Gaussian 16 (DFT APFD/6-311G++(2d,p), solvent MeOH). 49
Figure S41. LC-MS chromatograms of *Cr*DPAS and mutants reacted with substrate strictosidine aglycone and cofactor NADPH.. 50
Figure S42. Previously characterised reactions in monoterpene indole alkaloid biosynthesis catalysed by ADHs. Reactions A-F are 1,2-iminium reductions, reactions G-H are 1,4 unsaturated aldehyde reductions and reaction I is 1,4-iminium reduction. 51
Supporting Tables 52
Table S1. Primer sequences used in this study. 52
Table S2. Full length sequences of codon optimized genes used in this study. 53
Table S3. Summary of X-ray data and model parameters for *Cr*DPAS 54
Table S4. Summary of X-ray data and model parameters for apo-*Ti*DPAS2 55
Table S5. Summary of X-ray data and model parameters for precondylocarpine acetate-bound *Ti*DPAS2 56
Table S6. Summary of X-ray data and model parameters for stemmadenine acetate-bound *Ti*DPAS2 57
Table S7. Summary of X-ray data and model parameters for *Cr*GS 58
Table S8. Genbank accession for sequences used to construct tree of maximum likelihood in Figure 4A. 59
References 60

Experimental Procedures

Chemicals and molecular biology reagents

All solvents used for extractions, chemical synthesis and preparative HPLC were HPLC grade, and solvents used for UPLC/MS were MS grade. All solvents were purchased from Sigma Aldrich. Carbenicillin, kanamycin sulfate, isopropyl β-D-thiogalactoside (IPTG) salts were purchased from Sigma. Synthetic genes were purchased from IDT. All gene amplifications and mutations were performed using Platinum II Superfi DNA Polymerase (Thermo Fisher). Constructs were transformed into vectors using In-Fusion kit (ClonTech Takara) and colony PCR was performed using Phire II mastermix (Thermo Fisher) according to manufacturer's instructions. PCR product purification was performed using Zymoclean Gel DNA Recovery kit (Zymo). Plasmid purification was performed using the Wizard Miniprep kit (Promega). Strictosidine, precondylocarpine acetate, stemmadenine acetate, angryline, vincadifformine, 19-*E*-geissoschizine and tetrahydroalstonine were enzymatically prepared and purified as previously described [1–4].

Cloning and mutagenesis

Cloning of *Cr*DPAS, *Ti*DPAS1, *Ti*DPAS2, *Cr*GS and *Cr*THAS has been previously reported [1,2,4,5]. Full-length *Cr*DPAS, *Ti*DPAS2, GS and THAS were amplified by PCR from the codon optimized synthetic genes listed in Table S2 using corresponding primers listed in Table S1. *Thermoanaerobacter brockii* alcohol dehydrogenase (*Tb*ADH) synthetic gene (Table S2) was cloned into the pOPINF vector. DPAS, GS and THAS mutants were generated by overlap extension PCR as previously reported [6]. PCR products were purified from 1% agarose gel and ligated into the BamHI and KPNI restriction sites of pOPINK vectors for small-scale GS and GS mutants. All other ADHs were cloned into pOPINF vector. pOPINF and pOPINK were a gift from Ray Owens (Addgene plasmid # 26042 and # 41143 [7]). Constructs were ligated into vectors using the In-Fusion kit (Clontech Takara).

Expression and purification of proteins in *E. coli*

Constructs were transformed into chemically-competent *E. coli* Stellar cells (Clontech Takara) by heat shock at 42°C for 30 seconds and selected on LB agar containing 50µg/mL carbenicillin or kanamycin for pOPINF or pOPINK constructs respectively. Positive colonies were screened by colony PCR using primers listed in Table S1 and grown overnight at 37°C shaking at 200 r.p.m. Plasmids were then isolated and constructs were sequence verified. Plasmids were transformed into chemically competent *E. coli* SoluBL21 cells by heat shock for 30 seconds at 42°C and selected on LB agar containing 50 µg/mL carbenicillin or kanamycin for pOPINF or pOPINK constructs respectively. For small scale protein purification, 10 mL starter cultures of LB with 50 µg/mL of the respective antibiotic and a colony of transformed construct in SoluBL21 cells were grown at 37°C 200 r.p.m. overnight. Media (100 mL 2xYT media) containing 50 µg/mL antibiotic was inoculated with 1 mL of the starter culture and grown until OD_{600} of 0.6 was reached. For large scale purification, 20 mL starter cultures of LB with antibiotic and a colony of transformed construct in SoluBL21 cells were grown at 37°C 200 r.p.m. overnight. Media (1L 2xYT media) containing 50 µg/mL carbenicillin was inoculated with 10 mL of starter culture and grown until OD600 of 0.6 was reached. Once cultures had reached the desired OD_{600}, cultures were transferred to 18°C 200 r.p.m shaking incubator for 30 minutes before protein expression was induced by addition of 300 µM IPTG, after which cultures were grown for an additional 16 hours.

*Cr*PAS insect cell expression

N-terminal His_6-tagged *Cr*PAS was expressed in Sf9 insect cells as previously described [1]. Cells were harvested by centrifugation and the pellets frozen at –80°C until large-scale purification.

*Cr*DPAS, *Cr*GS and *Cr*THAS small-scale protein expression and purification

Cells were harvested by centrifugation at 4000 x *g* for 15 minutes and re-suspended in 10 mL buffer A1 (50 mM Tris-HCl pH 8, 50 mM glycine, 500 mM NaCl, 5% glycerol, 20 mM imidazole) with addition of EDTA-free protease inhibitor cocktail (Roche Diagnostics Ltd.) and 10 mg lysozyme (Sigma). Cells were lysed at 4°C using a sonicator (40% amplitude, 2 seconds on, 3 seconds off cycles for 2 minutes) and centrifuged at 35000 x *g* to remove insoluble cell debris. The supernatant was collected and filtered with 0.2 um PES syringe filter (Sartorious) and purified by addition of 150 µL washed Ni-NTA agarose beads (QIAGEN). Samples were incubated on a rocking incubator at 4°C for 1 hour. Beads were washed by centrifuging at 1000 x *g* for 1 minute to remove the supernatant, and then

the beads were resuspended in 10 mL of A1 Buffer. This step was performed a total of three times. Protein was eluted by resuspending the beads in 600 µL of buffer B1 (50 mM Tris-HCl pH 8.0, 50 mM glycine, 500 mM NaCl, 5% glycerol, 500 mM imidazole) before centrifuging for 1000 x *g* for 1 minute and then collecting the supernatant. This elution step was repeated to remove all Ni-NTA bound protein. Proteins were buffer exchanged into buffer A4 (20 mM HEPES pH 7.5, 150 mM NaCl) and concentrated using 10K Da molecular weight cut off centrifugal filter (Merck) and stored at –80°C.

_Cr_DPAS, _Ti_DPAS2, _Cr_GS, _Cr_SGD, _Cr_PAS and _Tb_ADH large-scale purification

Cells were harvested by centrifugation at 3200 x *g* for 15 minutes and re-suspended in 50 mL buffer A1 (50 mM Tris-HCl pH 8, 50 mM glycine, 500 mM NaCl, 5% glycerol, 20 mM imidazole) with addition of EDTA-free protease inhibitor cocktail (Roche Diagnostics Ltd.) and 10 mg lysozyme (Sigma). Dithiothreitol (Sigma) (final concentration of 0.05 mM) was additionally added to all buffers in purification of *Cr*DPAS, *Ti*DPAS2 and *Cr*GS for crystallisation. Cells were lysed at 4°C using a cell disruptor at 30 KPSI and centrifuged (35000 x *g*) to remove insoluble cell debris. The supernatant was collected and filtered with 0.2 µm PES syringe filter (Sartorious) and purified using an AKTA pure FPLC (Cytiva). Sample was applied at 2 mL/min onto a His-Trap HP 5mL column (Cytiva) and washed with 5 column volumes (CV) of buffer A1 before being eluted with 5 CV of buffer B1. Protein was detected and collected using the UV 280 nm signal and then further purified on a Superdex Hiload 16/60 S200 gel filtration column (Cytiva) at a flow rate of 1 mL/min using buffer A4. Proteins were finally buffer exchanged into buffer A4 and concentrated using 10K Da molecular weight cut off centrifugal filter (Merck) before being snap frozen in liquid nitrogen and stored at -80°C.

For crystallisation of *Cr*DPAS,*Ti*DPAS2 *and Cr*GS, protein after gel filtration was incubated on a rocker overnight at 4°C with 3C protease to cleave the 6xHis-tag. Proteins were then passed through a 1mL HisTrap column (Cytiva) to remove the cleaved tag. Proteins were then buffer exchanged into buffer A4 (20 mM HEPES pH 7.5, 150 mM NaCl) containing 0.05 mM tris(2-carboxyethyl)phosphine (Sigma) and concentrated using 10K Da molecular weight cut off centrifugal filter (Merck) and stored at –80°C.

Synthesis of NADPD

Deuterated pro-*R*-NADPD was produced in vitro as previously described [8] with minor modifications. A 20 mL reaction mixture containing 2 mM $NADP^+$, 4 mM d_8-isopropanol, 1 mM semicarbazide and 5 µM *Tb*ADH in 50 mM ammonium bicarbonate buffer at pH 7.5 was incubated at 30°C. The progression of the reaction was monitored spectrophotometrically at 340 nm. When no significant increase in absorbance was observed (approximately 3 hours), 300 µL of Ni-NTA agarose beads (Qiagen) was added and the sample incubated rocking at room temperature for 30 minutes. The reaction was centrifuged to remove the Ni-NTA beads bound to *Tb*ADH, and the supernatant was filtered through a 45 µm glass filter and lyophilized to remove the unreacted d_8-isopropanol, the acetone that forms during the reaction and the buffer. The residue, containing primarily NADPD, was stored at –20°C until use.

In vitro enzyme assays

Enzymatic assays with precondylocarpine acetate were performed in 50 mM HEPES buffer (pH 7.5) with 50 µM precondylocarpine acetate in MeOH (not exceeding 5% of the reaction volume), 250 µM NADPH cofactor (Sigma) and 150 nM enzyme to a final reaction volume of 100 µL. Reactions were incubated for 30 minutes at 30°C and shaking at 60 r.p.m. before being quenched with 1 volume of 70% MeOH with 1% H_2CO_2. Enzymatic assays with strictosidine aglycone were performed in 50 mM HEPES buffer (pH 7.5), 100 µM strictosidine and 1 mM SGD to a final reaction volume of 100 µL. Assays were incubated for 30 minutes at 30°C and shaking at 60 r.p.m before 500 nM of ADH enzyme and 250 µM NADPH was added. As control, the reactions were performed without the addition of ADH enzyme. Reactions were incubated for a further 30 minutes at 30°C shaking at 60 r.p.m. before being quenched with 1 volume of 70% MeOH with 0.1% H_2CO_2. All enzymatic assays were centrifuged at 14000 x *g* for 15 minutes and the supernatant analysed by UPLC-MS.

UPLC-MS

All assays were analysed using a Thermo Scientific Vanquish UPLC coupled to a Thermo Q Exactive Plus orbitrap MS. For assays using precondylocarpine acetate, chromatographic separation was performed using a Phenomenex Kinetex C18 2.6 µm (2.1 x 100 mm) column using water with 1% H_2CO_2 as mobile phase A and acetonitrile with 1% H_2CO_2 as mobile phase B. Compounds were separated using a linear gradient of 10-30% B in 5 minutes followed by 1.5 minutes isocratic at 100% B. The column was then re-equilibrated at 10% B for 1.5 minutes. The column was heated to 40°C and flow rate was set to 0.6 mL/min. For assays using strictosidine aglycone, separation was carried out using a Waters Acquity BEH C18 1.7 µm (2.1 x 50 mm) using 0.1% NH_4OH in water as mobile phase A and acetonitrile as mobile phase B. Compounds were separated using a linear gradient of 10-90% B in 9

minutes followed by 2 minutes isocratic at 90% B. The column was re-equilibrated at 10% B for 3 minutes. The column was heated to 50°C and flow rate was set to 0.4 mL/min. MS detection was performed in positive ESI under the following conditions: spray voltage was set to 3.5 kV ~ 67.4 µA, capillary temperature set to 275°C, vaporizer temperature 475°C, sheath gas flow rate 65, sweep gas flow rate 3, aux gas flow rate 15, S-lens RF level to 55 V. Scan range was set to 200 - 1000 *m/z* and resolution at 17500.

Production and isolation of *d*-angryline and *d*$_2$-vincadifformine

d-angryline was produced enzymatically from stemmadenine acetate using the same protocol previously described for the synthesis of angryline but replacing NADPH with NADPD [6]. Briefly, 0.25 mg of stemmadenine acetate, 40 µM flavin adenine dinucleotide (FAD) and 5 µg of *Cr*PAS were combined in a total volume of 500 µL in 50 mM TRIS-HCl buffer pH 8.5 and incubated at 37°C to form precondylocarpine acetate (reaction progress was monitored by LC-MS, *m/z* 395.19). After 2 hours, 1 mg of NADPD and 9 µg of *Cr*DPAS were added to the reaction and incubated for 20 minutes at 37 °C to obtain *d*-angryline (*m/z* 338.19). Multiple reactions were prepared to obtain sufficient product for NMR characterization. After completion, the reactions were snap frozen in liquid nitrogen and stored at –80 °C.

d$_2$-vincadifformine was also produced enzymatically, but in this case NADPD was generated directly in the reaction mixture using an alcohol dehydrogenase from *E. coli* (Merck product 49854). Multiple 500 µL reactions were prepared to obtain sufficient product for NMR characterization. Each reaction contained 400 µM $NADP^+$, 0.89 µg *d*$_8$-isopropanol, 1 µg of *Tb*ADH, 10 µg stemmadenine acetate, 0.8 µM *Cr*PAS and 0.8 µM *Ti*DPAS1 in 50 mM HEPES buffer pH 7.5. The reactions were incubated at 30 °C for 1 hour, snap frozen in liquid nitrogen and stored at –80 °C until purification of the final product.

d-angryline and *d*$_2$-vincadifformine were purified by semi-preparative HPLC on an Agilent 1260 Infinity II HPLC system. The reactions were thawed and 500 µL of 90:9:1 MeOH:H_2O:H_2CO_2 was added to the deuterated samples. The samples were filtered through 0.2 µm PTFE disc filters (Sartorius) to remove the precipitated enzymes and injected onto a Phenomenex Kinetex XB-C18 5 µm (250 x 10 mm) column. Chromatographic separation was performed using 0.1% H_2CO_2 in water as mobile phase A and acetonitrile as mobile phase B. A linear gradient from 10% B to 40% B in 15 minutes was used for chromatographic separation of the compounds followed by a wash at 40% B for 5 minutes and a re-equilibration step to 10% B for 5 minutes. Flow rate was 6 mL/min. Elution of *d*-angryline and *d*$_2$-vincadifformine was monitored at two wavelengths, 330 and 254 nm. Fractions containing the compounds of interest were collected, dried under reduced pressure and stored at –80 °C until further analysis.

Production and isolation of 19,20-dihydrovallesiachotamine

19,20-dihydrovallesiachotamine was produced enzymatically from 100 µM strictosidine reacted with 100 µM *Cr*SGD in 50 mM HEPEs buffer pH 7.5 in a 100 mL reaction at 30°C. After 90 minutes, 500 nM of *Cr*DPAS and 250 µM NADPH was added and the reaction monitored. After 2 hours a further 500 nM *Cr*DPAS was added to a final concentration of 1 µM and left for a further 3 hours until the reaction reached completion. The sample was snap frozen in liquid nitrogen and stored at –80 °C. For purification, the sample was thawed on ice and filtered through a 0.2 µm PTFE disc filter (Sartorius) to remove the precipitated enzymes and then passed through a Supelco DSC-18 column (MilliporeSigma) and eluted with methanol. Eluent was dried down in a rotovap and resuspended in 1.5 mL methanol. The product was purified on an Agilent 1290 Infinity II semi-preparative HPLC system using a Waters XBridge BEH C18 5 µm (10 x 250mm) column and using 0.1% NH_4OH in water as mobile phase A and acetonitrile as mobile phase B. Compounds were separated using a linear gradient of 10-65% B in 25 minutes followed by 10 minutes column re-equilibration at 10% B. Flow rate was set to 7mL/min. Compound was detected by measuring UV 290 nm and 254 nm signal. Fractions containing the compound of interest were collected and dried down using a rotovap and stored at –20 °C until NMR analysis.

NMR of *d*-angryline, *d*-vincadifformine and 19,20-dehydrovallesiachotamine

For *d*-angryline, NMR spectra were measured on a 400 MHz Bruker Advance III HD spectrometer (Bruker Biospin GmbH, Rheinstetten, Germany). NMR spectra for 19,20-dehydrovallesiachotamine, (–)-vincadifformine and *d*$_2$-(±)-vincadifformine were measured on a 700 MHz Bruker Advance III HD spectrometer (Bruker Biospin GmbH, Rheinstetten, Germany). For spectrometer control and data processing Bruker TopSpin ver. 3.6.1 was used. MeOH-*d*$_3$ was used as a solvent and all NMR spectra were referenced to the residual solvent signals at δH 3.31 and δC 49.0, respectively.

ECD measurement

ECD spectra were measured at 25 °C on a JASCO J-810 spectropolarimeter (JASCO cooperation, Tokyo, Japan) using a 350 µL cell. Spectrometer control and data processing was accomplished using JASCO spectra manager II.

ECD spectral calculations for (-)-vincadifformine

Based on the structure determined from NMR analysis a molecular model was created in GaussView ver.6 (Semichem Inc., Shawnee, Kansas, USA) and optimized using the semi-empirical method PM6 in Gaussian (Gaussian Inc., Wallingford, Connecticut, USA). The resulting structure was used for conformer variation with the GMMX processor of the Gaussian program package. Resulting structures were DFT-optimized with Gaussian ver.16 (APFD/6-31G(d)). A cut-off level of 4 kcal/mol was used to select conformers which were subjected to another DFT optimization on a higher level (APFD/6-311G+(2d,p)). All structures up to a deviation of 2.5 kcal/mol from the lowest energy conformer were used to determine the ECD-frequencies in a TD-SCF calculation on the same level as the former DFT optimization. The ECD curve was calculated from the Boltzmann-weighed contributions of all conformers with a cut-off level of two percent.

Experimentally measured ECD data and calculated data were compared using SpecDis ver.1.71 [9].

Protein crystallisation

Purified *Cr*DPAS and *Ti*DPAS2 were crystallised by sitting-drop vapour diffusion on MRC2 96-well crystallisation plates (SwissSci) with 0.3 uL protein and 0.3 uL precipitant solution drops dispensed by Oryx8 robot (Douglas Instruments). *Cr*DPAS was crystallised using JCSG screen (Jena Biosciences) with 1.26 M ammonium sulfate, 100 mM TRIS buffer pH 8.5 and 200mM lithium sulfate. Crystallisation condition with additional 1 mM $NADP^+$ and 25% ethylene glycol was used as cryoprotectant. *Ti*DPAS2 was initially screened using PEG/Salt screen (Jena Biosciences) before condition optimization. Apo-*Ti*DPAS2 was crystallised in 17% w/v PEG 3350, 200 mM ammonium chloride and 0.75 mM angryline (no electron density corresponding to angryline was observed in the structure). 17% w/v PEG 3350, 220 mM ammonium chloride, 1 mM $NADP^+$, 1 mM angryline and 25% ethylene glycol was used as cryoprotectant. Stemmadenine acetate-bound *Ti*DPAS2 was crystallised in 23% w/v PEG 3350, 250 mM sodium sulfate and 0.75 mM stemmadenine acetate, 23% w/v PEG 3350, 200 mM sodium sulfate, 1 mM $NADP^+$, 1 mM stemmadenine acetate and 25% ethylene glycol was used as cryoprotectant. Precondylocarpine acetate-bound *Ti*DPAS2 was crystallised in 25% w/v PEG 3350, 180 mM sodium sulfate and 0.75 mM precondylocarpine acetate. 23% w/v PEG 3350, 200mM sodium sulfate, 1 mM $NADP^+$, 1 mM precondylocarpine acetate and 25% ethylene glycol was used as cryoprotectant. *Cr*GS was crystallised in 25% w/v PEG 3350, 100 mM TRIS buffer pH8.0; 20% v/v ethylene glycol was added to this condition for the cryoprotectant. All crystals were soaked in the corresponding cryoprotectant before flash-cooling in liquid nitrogen.

X-ray data collection, processing and structure solution

X-ray data sets for *Cr*DPAS and *Ti*DPAS2 structures were recorded on the 10SA (PX II) beamline at the Paul Scherrer Institute (Villigen, Switzerland) at wavelength of 1.0 .Å using a Dectris Eiger3 16M detector with the crystals maintained at 100K by a cryocooler. Diffraction data were integrated using XDS [12] and scaled and merged using AIMLESS [14]; data collection statistics are summarized in Table S3-7. Structure’s solution was automatically obtained by molecular replacement using the structure of tetrahydroalstonine synthase from *C. roseus* (PDB accession code 5FI3) as template with which *Cr*DPAS and *Ti*DPAS2 share 54% and 56% amino acid identity respectively. In all cases the map was of sufficient quality to enable 90% of the residues expected for a homodimer to be automatically fitted using Phenix autobuild [10]. The models were finalized by manual rebuilding in COOT [11] and refined using in Phenix refine.

X-ray data for *Cr*GS was recorded at 100 K on beamline I03 at the Diamond Light Source (Oxfordshire, UK) using a Pilatus3 6M hybrid photon counting detector (Dectris), with crystals maintained at 100 K by a Cryojet cryocooler (Oxford Instruments). Diffraction data were integrated and scaled using XDS [12] via the XIA2 expert system [13] then merged using AIMLESS [14] A summary of the data processing is presented in Table S7. A template for molecular replacement was prepared with CHAINSAW [15] from the structure of tetrahydroalstonine synthase from *C. roseus* (PDB accession code 5FI3) with which *Cr*GS shares 57% amino acid sequence identity. The structure was solved by molecular replacement using PHASER [16] , giving two copies of the subunit in the asymmetric unit, which formed the homodimeric assembly expected for this class of enzyme. After restrained refinement in REFMAC5 [17] at 2.0 Å resolution, the protein component of the model was completely rebuilt using BUCCANEER [18]. The model was finalized after several iterations of manual editing in COOT [11] and further refinement in REFMAC5 incorporating TLS restraints. The model statistics are reported in Table S8.

All structures are in the PDB database under the following accessions: 8B27 (*Cr*DPAS), 8B26 (apo-*Ti*DPAS2), 8B1V (precondylocarpine acetate-bound *Ti*DPAS2), 8B25 (stemmadenine acetate-bound *Ti*DPAS2), 8A3N (*Cr*GS).

Docking simulations

Ligands were docked into the active site of *Ti*DPAS and *Cr*GS using AutoDock Vina on the Webina webserver using default parameters [19,20]. Coordinates of ligands were generated by PDBQTConvert. When assessing the results, we selected ligand orientations in which the 4-pro-*R* hydride of NADPH was in close proximity to the carbon being reduced; this orientation was not always the lowest possible energy solution. Results were visualised using PyMOL. Cavity pocket size estimation was computed using CASTp3.0 using default parameters [21]. Results were visualised using Chimera.

Phylogenetic analysis

Nucleic acid sequences of ADH genes were aligned using MUSCLE5 [22]. A maximum likelihood phylogenetic tree was constructed using IQTree using a best-fit substitution model followed by tree reconstruction using 1000 bootstrap alignments and the remaining parameters used default settings [23]. Tree visualisation and figures were made using iTOL version 6.5.2 [24].

Supporting Figures

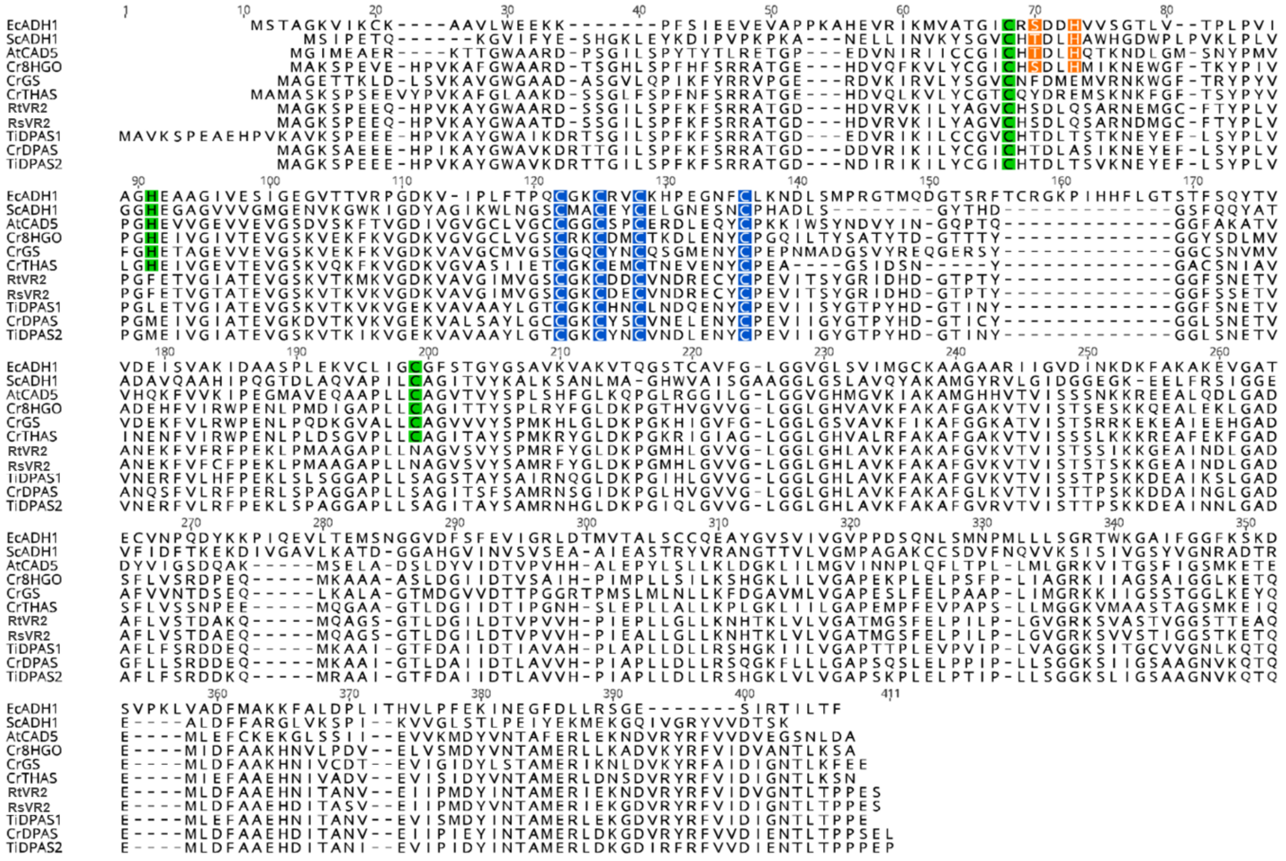

Figure S1. MUSCLE amino acid sequence alignment of ADHs highlighting key residues. Catalytic zinc coordinating residues are labelled in green, structural zinc coordinating residues in blue and proton relay residues in orange. Protein names and uniprot accessions: *Equus caballus* alcohol dehydrogenase (*Ec*ADH1) P00327; *Saccharomyces cerevisiae* alcohol dehydrogenase 1 (*Sc*ADH1), P00330; *Arabidopsis thaliana* cinnamyl alcohol dehydrogenase 5 (*At*CAD5), O49482; *Catharanthus roseus* 8-hydroxygeraniol dehydrogenase (*Cr*8HGO), Q6V4H0; *Catharanthus roseus* geissoschizine synthase (*Cr*GS), W8JWW7; *Catharanthus roseus* tetrahydroalstonine synthase (*Cr*THAS), A0A0F6SD02; *Rauwolfia tetraphylla* vomilenine reductase 2 (*Rt*VR2) A0A0U4BHM2, *Rauwolfia serpentina* vomilenine reductase 2 (*Rs*VR2), A0A0U3S9Q3; *Tabernanthe iboga* dihydroprecondylocarpine acetate synthase 1 (*Ti*DPAS1), A0A5B8XAH0; *Catharanthus roseus* dihydroprecondylocarpine acetate synthase (*Cr*DPAS), A0A1B1FHP3; *Tabernanthe iboga* dihydroprecondylocarpine acetate synthase 2 (*Ti*DPAS2), A0A5B8X8Z0.

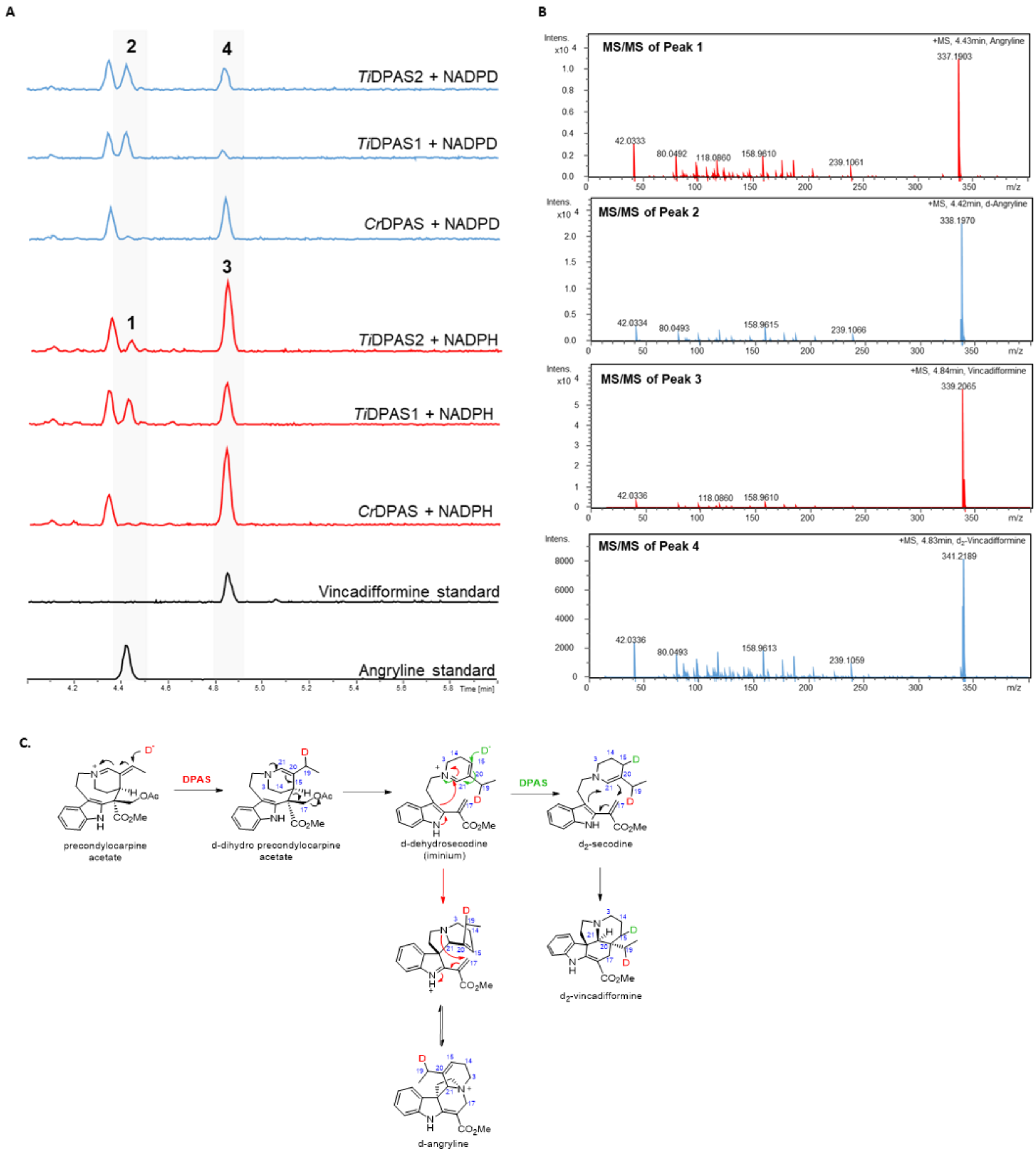

Figure S2. Deuterium labelling of DPAS reduction of precondylocarpine acetate. **A.** TIC LC/MS chromatograms of reactions product of precondylocarpine acetate substrate reacted with DPAS in the presence of NADPH or deuterated NADPD. Inset of MS/MS2 spectra of angryline, d-angryline, vincadifformine and d2-vincadifformine products. **B.** MS/MS of angryline, d-angryline, vincadiffromine and d2-vincadifformine. **C.** Proposed mechanism of DPAS reduction of precondylocarpine acetate.

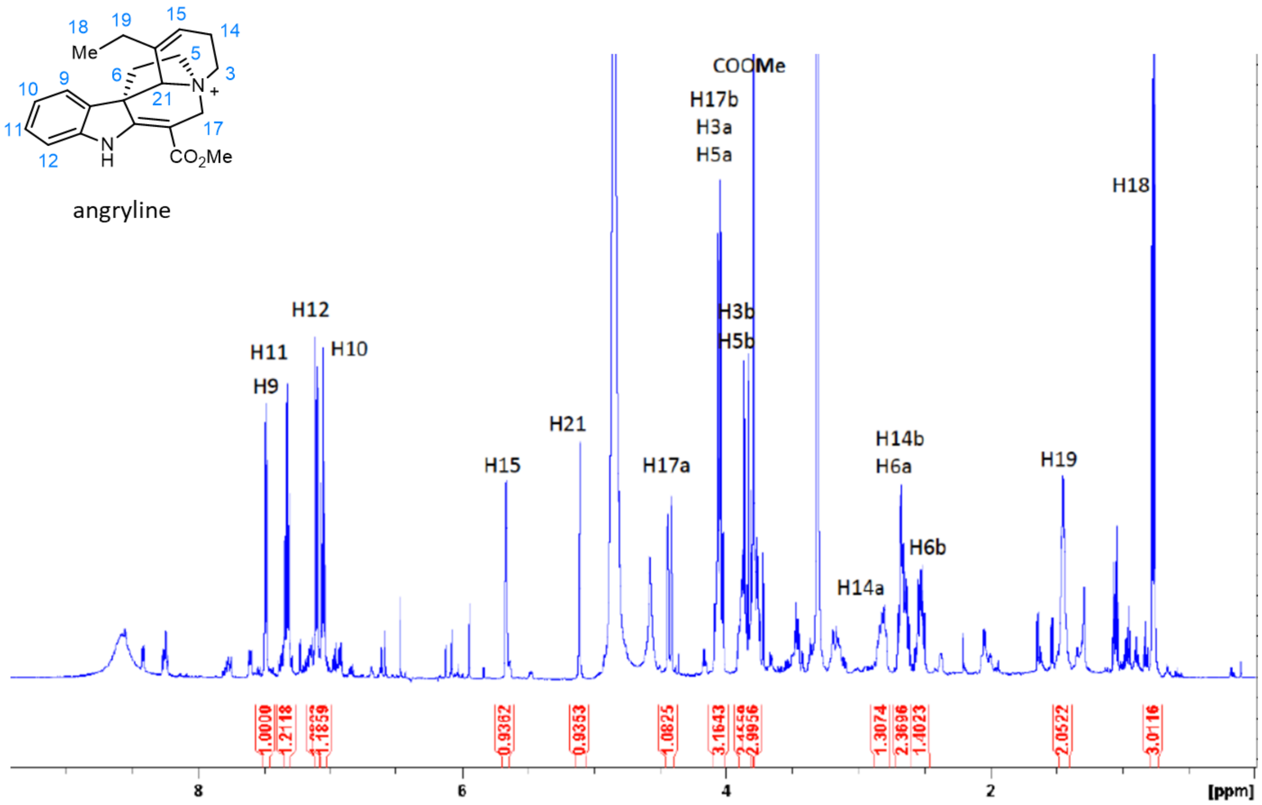

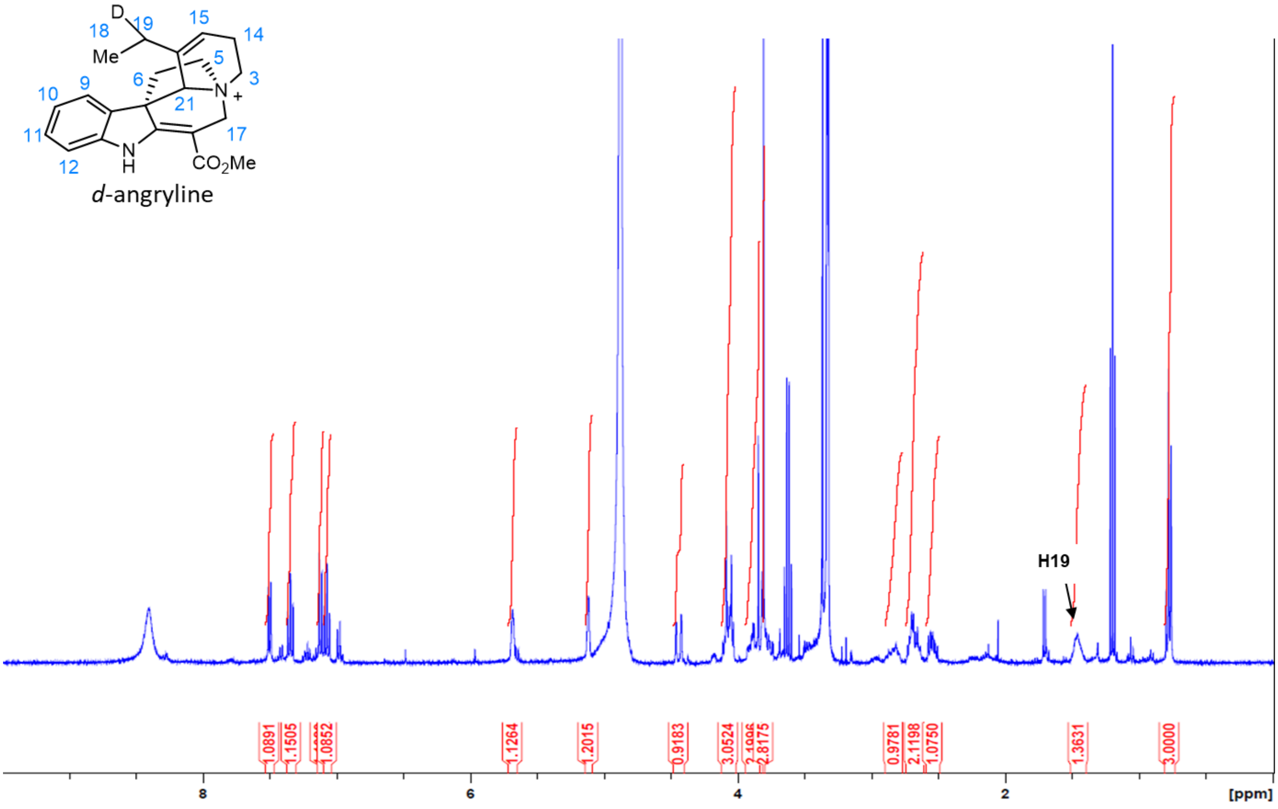

Figure continued on page 12.

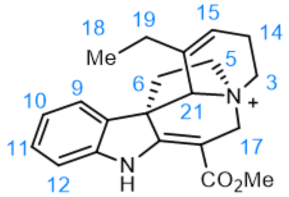

angryline
^{1}H 600 MHz,
MeOH-d_3, 25 °C

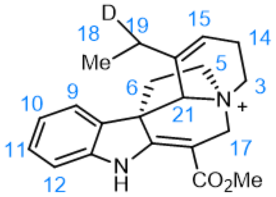

d-angryline
^{1}H 400MHz,
MeOH-d_3, 25 °C

Position	angryline[6] ^{1}H (600 MHz, MeOH-d_3)	d-angryline ^{1}H (400 MHz, MeOH-d_3)
3	4.05 (m, 1H)<br>3.76 (m, 1H)	4.04 (m, 1H)<br>3.82 (m,1H)
5	4.06 (m, 1H)<br>3.88 (m, 1H)	4.07 (m, 1H)<br>3.85 (m, 1H)
6	2.66 (m, 1H)<br>2.51 (m, 1H)	2.69 (m, 1H)<br>2.54 (m, 1H)
9	7.49 (d, 7.5, 1H)	7.50 (d, 7.5, 1H)
10	7.06 (dd, 7.6, 7.6, 1H)	7.07 (td, 7.5, 0.9, 1H).
11	7.33 (dd, 7.8, 7.8, 1H)	7.35 (td, 7.8, 1.1, 1H)
12	7.10 (d, 7.6, 1H)	7.12 (d, 7.9, 1H),
14	2.82 (m, 1H)<br>2.66 (m, 1H)	2.85 (m, 1H)<br>2.69 (m, 1H)
15	5.66 (s, 1H)	5.68 (s, 1H)
17	4.41 (dd, 15.0, 2.0, 1H) 4.06 (m, 1H)	4.44 (dd, 15.0, 2.2, 1H)<br>4.07 (m, 1H)
18	0.78 (t, 7.3, 3H)	0.78 (t, 7.4, 3H).
19	1.43 (m, 2H)	1.46 (m, 1.36H)
21	5.11 (s, 1H)	5.12 (s, 1H)
CO_2Me	3.78 (s, 3H)	3.81 (s, 3H)

Figure S3. Comparison of ^{1}H NMR data for angryline and *d*-angryline showing position of deuterium incorporation. Angryline was characterised by NMR in a previous study [6].

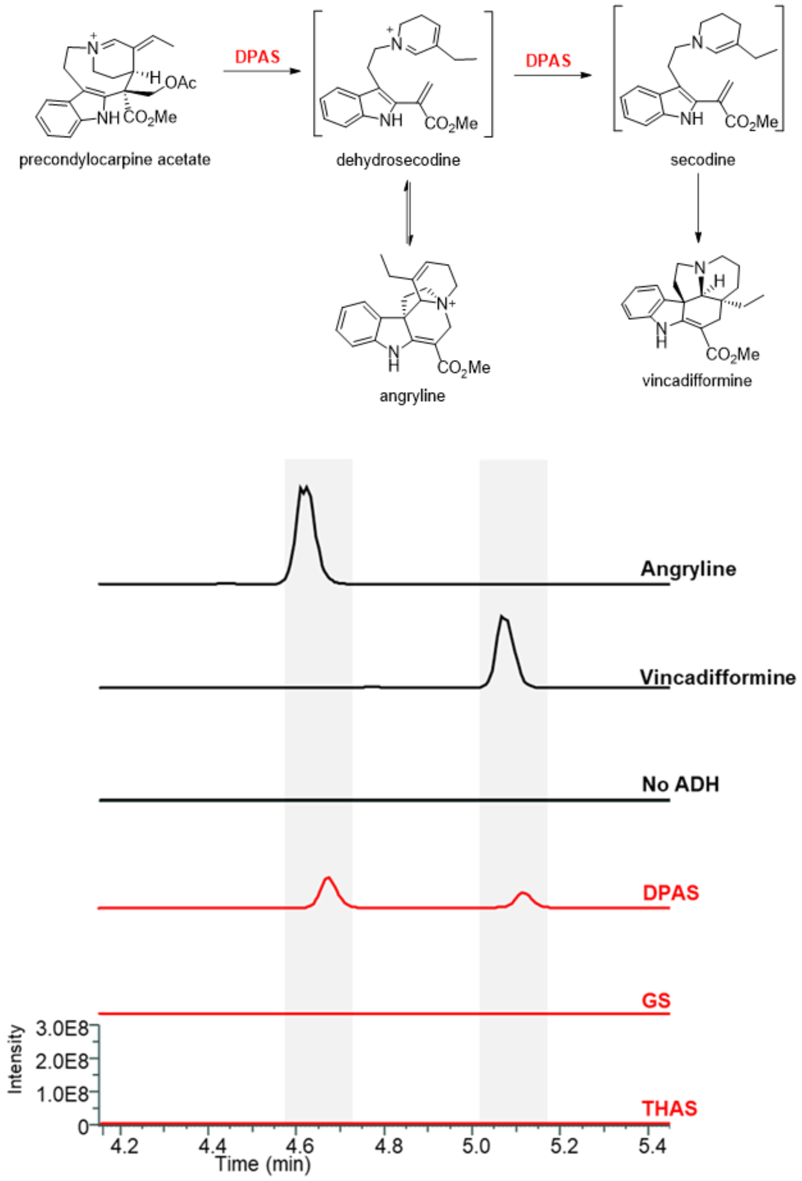

Figure S4. LC-MS chromatograms showing formation of angryline and vincadifformine when reacting *Catharanthus roseus* ADHs DPAS, GS and THAS with susbtrate precondylocarpine acetate in the presence of cofactor NADPH and in absence of any cyclase enzyme. EIC *m/z* 337.00-341.00.

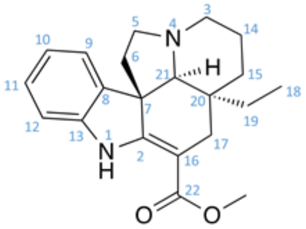

(-)-vincadifformine
^{1}H 700 MHz, MeOH-d_3, 25 °C

pos.	δ_H	mult.	J_{HH}	δ_C
1	9.16	bs	-	-
2	-	-	-	169.1
3a	2.45	ddd	10.9/10.9/3.0	51.4
3b	3.11	m	-	51.4
4	-	-	-	-
5a	2.59	ddd	11.3/8.5/4.8	52.5
5b	2.91	m	-	52.5
6a	1.64	ddd	11.3/4.8/1.2	46.6
6b	1.99	ddd	11.3/11.3/6.6	46.6
7	-	-	-	57.0
8	-	-	-	138.9
9	7.20	bd	7.2	121.8
10	6.84	bdd	7.7/7.2	121.5
11	7.09	ddd	7.7/7.7/0.9	128.5
12	6.89	bd	7.7	110.5
13	-	-	-	144.8
14a	1.54	m	-	22.8
14b	1.84	m	-	22.8
15a	1.29	ddd	13.1/13.1/4.9	33.9
15b	1.80	m	-	33.9
16	-	-	-	92.8
17a	2.28	dd	15.2/1.8	26.9
17b	2.70	d	15.2	26.9
18	0.57	t	7.3	7.3
19a	0.95	m	-	30.4
19b	0.63	m	-	30.4
20	-	-	-	39.0
21	2.51	bs	-	73.8
22	-	-	-	170.2
OMe	3.74	s	-	51.3

Multiplicity abbreviations: s, singlet; d, doublet; m, multiplet; bs, broad signlet; bd, broad doublet; dd, doublet of doublets; bdd, broad doublet of doublets; ddd, doublet of doublet of doublets.

Figure S5. ^{1}H NMR data for (-)-vincadifformine in MeOH-d_3.

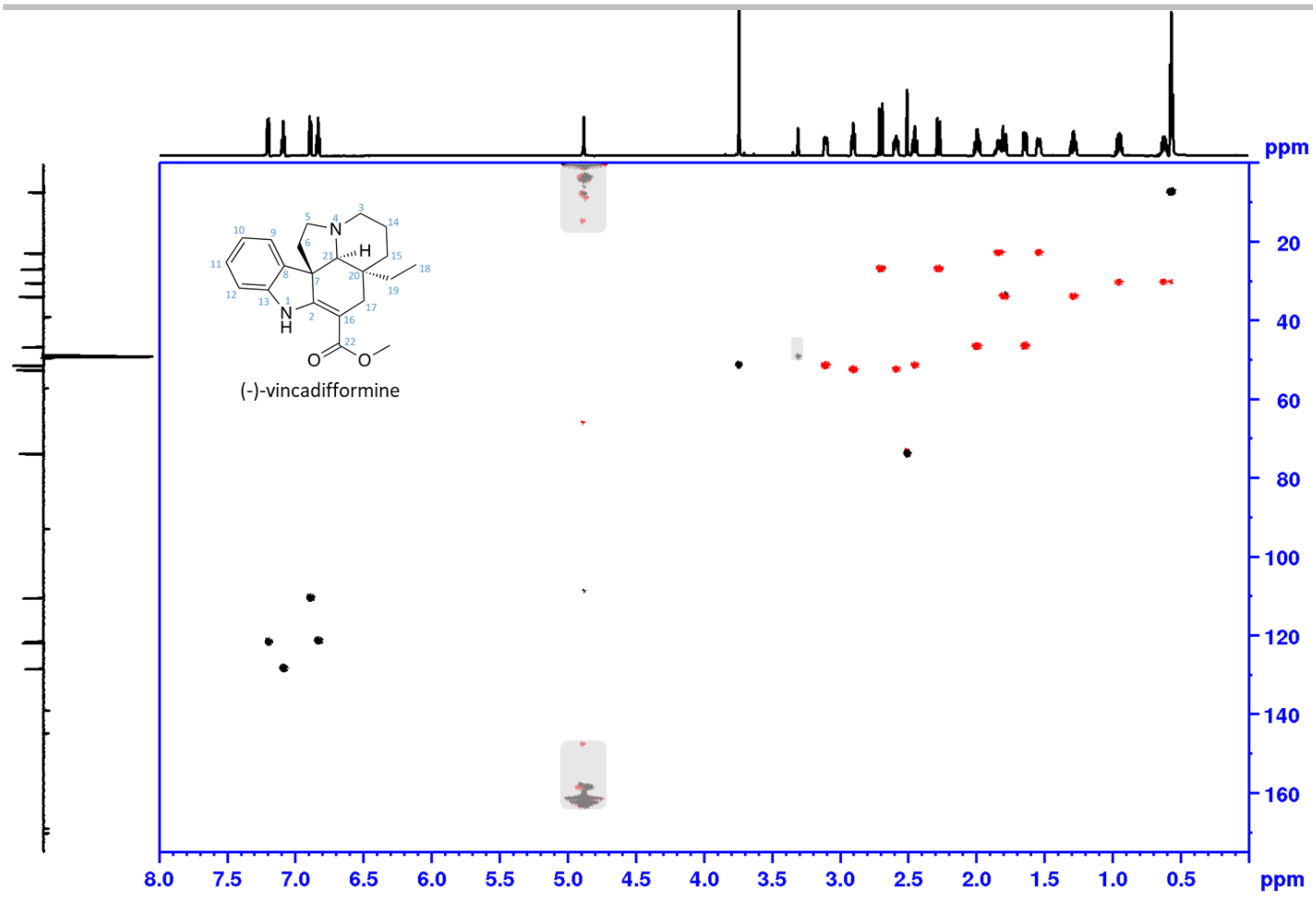

Figure S6. ^{1}H NMR data for *m/z* 339, (–)-vincadifformine (standard). Phase sensitive HSQC, full range in MeOH-d_3. Shaded areas mark impurity and solvent, red: CH2, black: CH, CH3. NMR data of (–)-vincadifformine in chloroform-*d* has been previously reported [25,26].

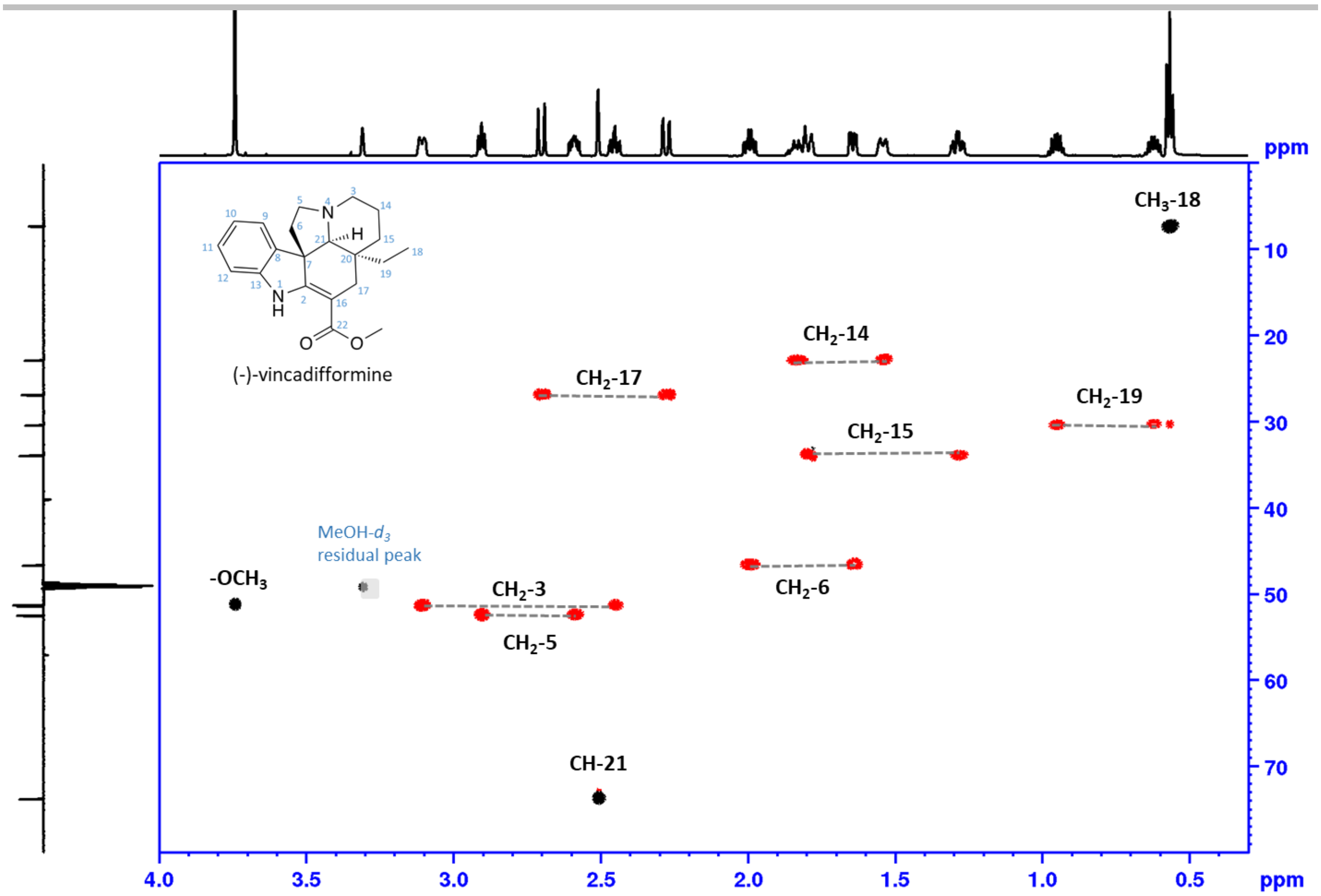

Figure S7. ^{1}H NMR data for *m/z* 339, (–)-vincadifformine (standard). Phase sensitive HSQC, aliphatic range in MeOH-d3. Shaded areas mark impurity and solvent, red: CH2, black: CH, CH3

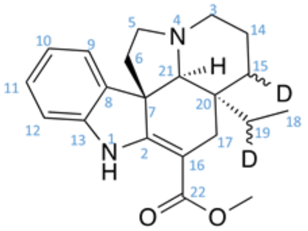

d_2-(±)-vincadifformine
^{1}H 700 MHz, MeOH-d_3,
25 °C

pos.	δ_H	mult.	J_{HH}	δ_C
1	9.18	bs	-	-
2	-	-	-	169.1
3a	2.51	m**	-	51.3
3b	3.14	m	-	51.3
4	-	-	-	-
5a	2.65	ddd	11.3/9.3/4.8	52.5
5b	2.95	m	-	52.5
6a	1.69	ddd	11.3/4.8/1.2	46.6
6b	2.03	ddd	11.3/11.3/6.4	46.6
7	-	-	-	56.9
8	-	-	-	138.8
9	7.23	bd	7.3	121.8
10	6.85	bdd	7.7/7.3	121.6
11	7.1	ddd	7.7/7.7/0.8	128.5
12	6.91	bd	7.7	110.6
13	-	-	-	144.8
14a	1.56	m	-	22.4
14b	1.85	m	-	22.4
15a*	1.30	m **	-	33.2
15b*	1.79	m	-	33.2
16	-	-	-	92.8
17a	2.30	dd	15.1	26.9
17b	2.69	dd	15.2/2.9	26.9
18	0.57	d	7.3	7.1
19a*	0.94	m**	-	30.1
19b*	0.63	m**	-	30.1
20	-	-	-	38.7
21	2.59	bs	-	73.6
22	-	-	-	170.2
OMe	3.75	s	-	51.4
*as CH signal				
** overlapped signals *J* unresolved				

Multiplicity abbreviations: s, singlet; d, doublet; m, multiplet; bs, broad signlet; bd, broad doublet; dd, doublet of doublets; bdd, broad doublet of doublets; ddd, doublet of doublet of doublets.

Figure S8. ^{1}H NMR data for d_2-(±)-vincadifformine in MeOH-d_3.

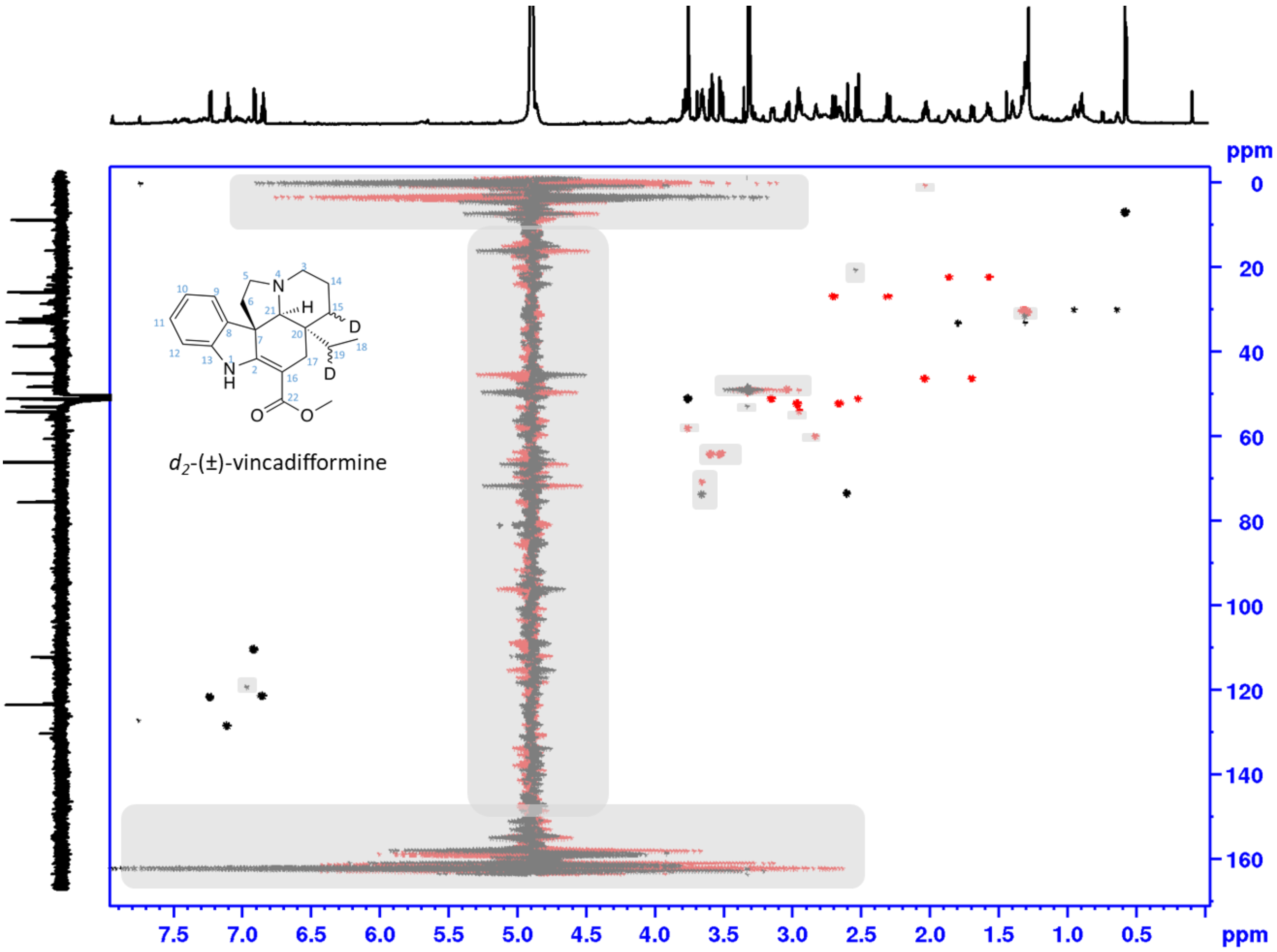

Figure S9. Phase sensitive HSQC NMR data for *m/z* 341, d_2-(±)-vincadifformine full range in MeOH-d_3. Shaded areas mark impurity and solvent, red: CH2, black: CH, CH3

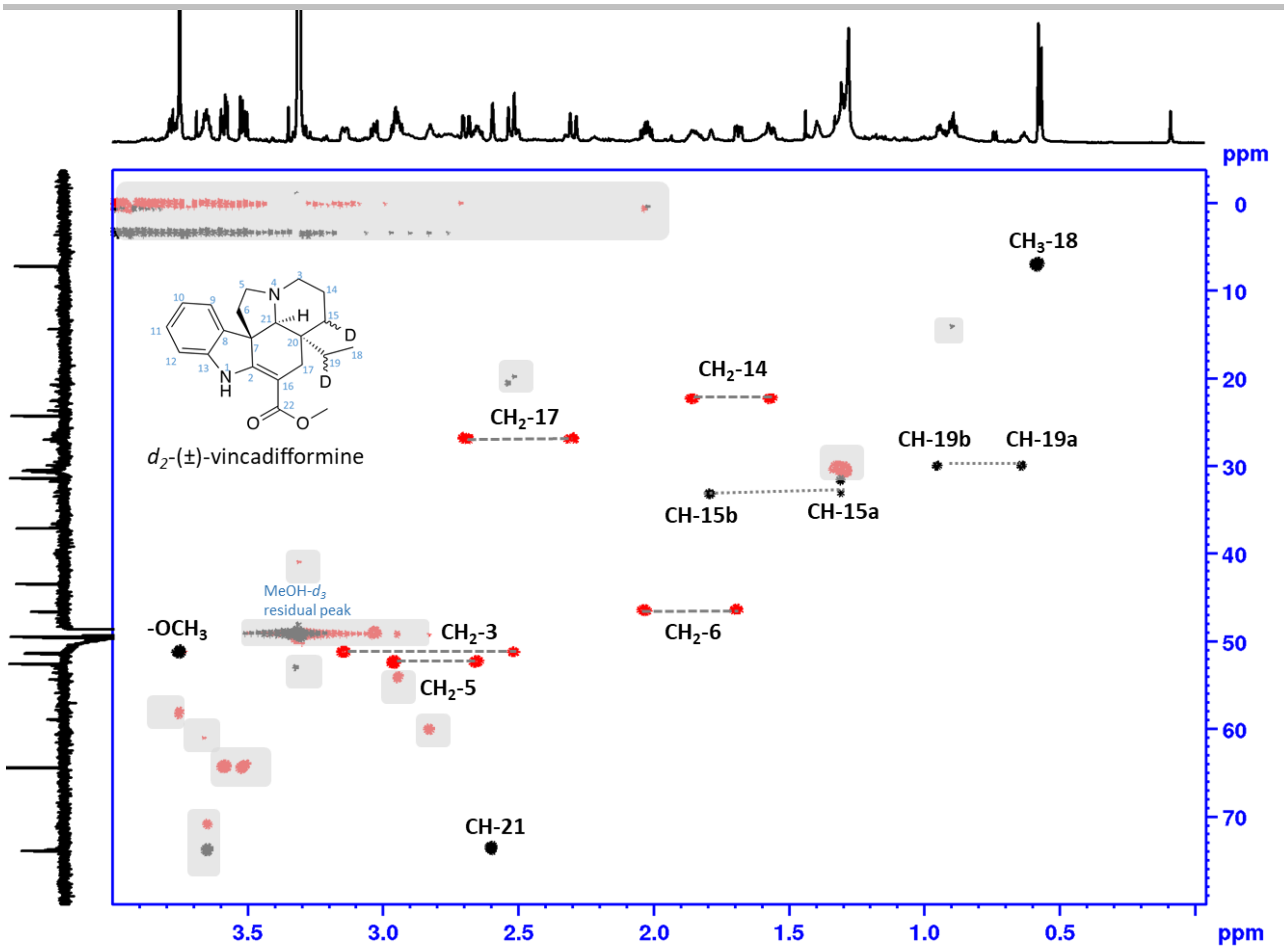

Figure S10. Phase sensitive HSQC NMR data for *m/z* 341, *d*2-(±)-vincadifformine, aliphatic range in MeOH-*d*3. Shaded areas mark impurity and solvent, red: CH2, black: CH, CH3

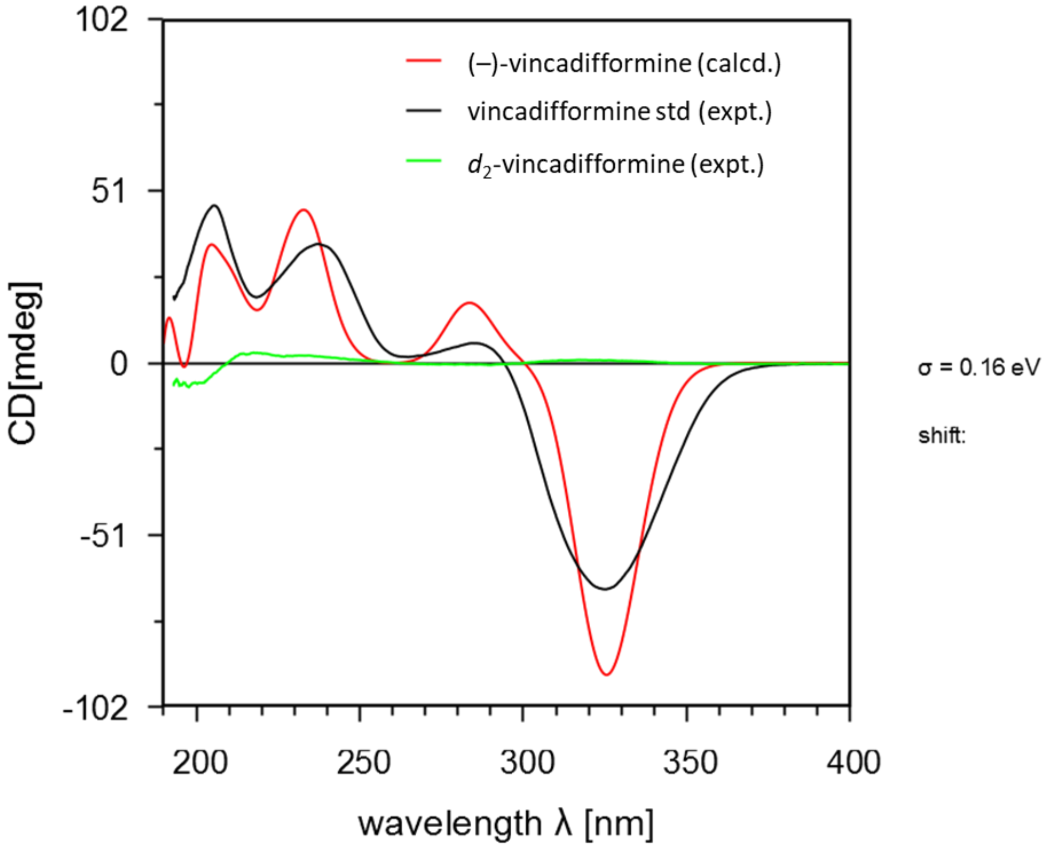

Figure S11. Comparison of the experimental ECD spectra of d_2-vincadifformine (green), vincadifformine standard (black) and calculated ECD spectra of (-)-vincadifformine (red).

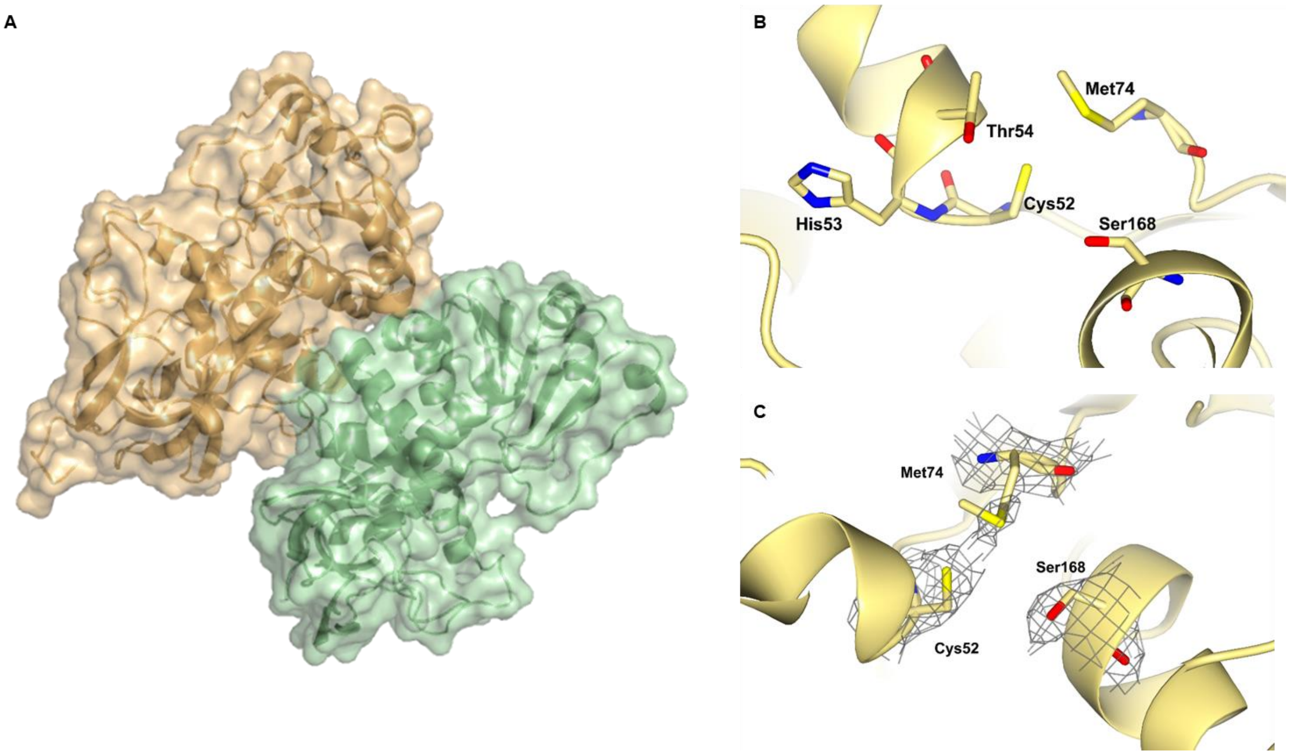

Figure S12. Structure of *Catharanthus roseus* DPAS (*Cr*DPAS) crystallised as a homodimer at 2.45 Å resolution. **A.** Structure coloured by chains. Structure lacked electron density for residues Gly102-Thr134. **B.** Active site of *Cr*DPAS showing atypical residues canonically involved in coordination of the catalytic zinc. **C.** Electron density of *Cr*DPAS residues canonically involved in coordinating the catalytic zinc.

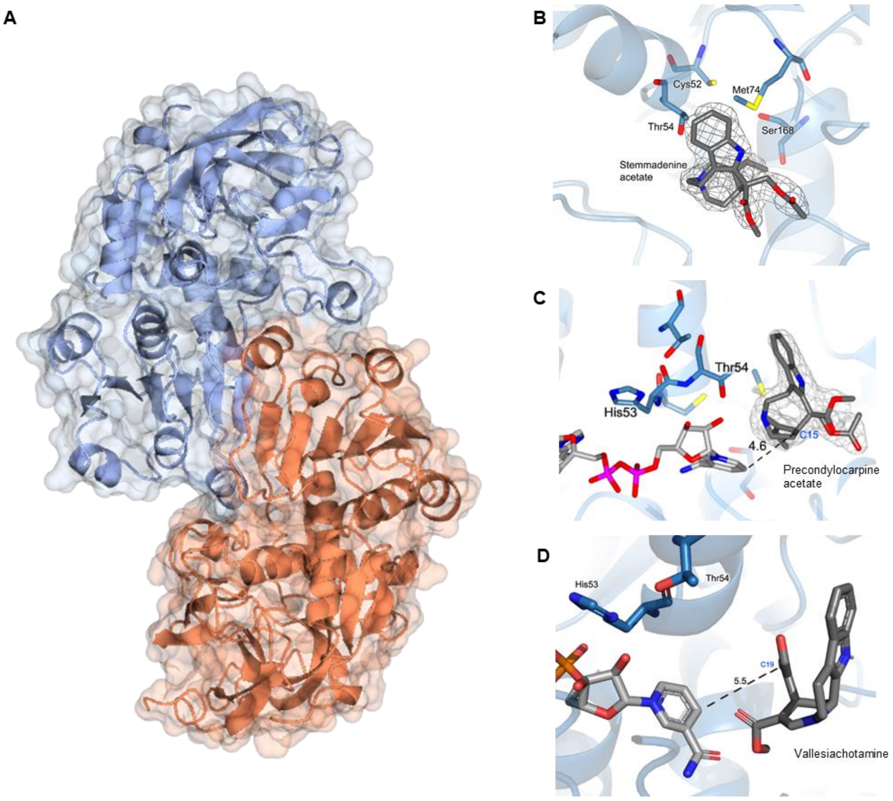

Figure S13. Structure of *Tabernanthe iboga* DPAS2 (*Ti*DPAS2). **A.** Apo-*Ti*DPAS2 crystallised as a homodimer at 2.42 Å coloured by chains. Electron density for $NADP^+$ cofactor was not observed. **B.** Active site of *Ti*DPAS2 bound to stemmadenine acetate. **C.** *Ti*DPAS2 bound to precondylocarpine acetate and docked with NADPH cofactor. Measurement shows distance between the 4-pro-*R*-hydride of NADPH and position of reduction at C15 of precondylocarpine acetate. **D.** *Ti*DPAS2 docked with vallesiachotamine and NADPH cofactor showing distance between the 4-pro-*R*-hydride of NADPH and position of reduction.

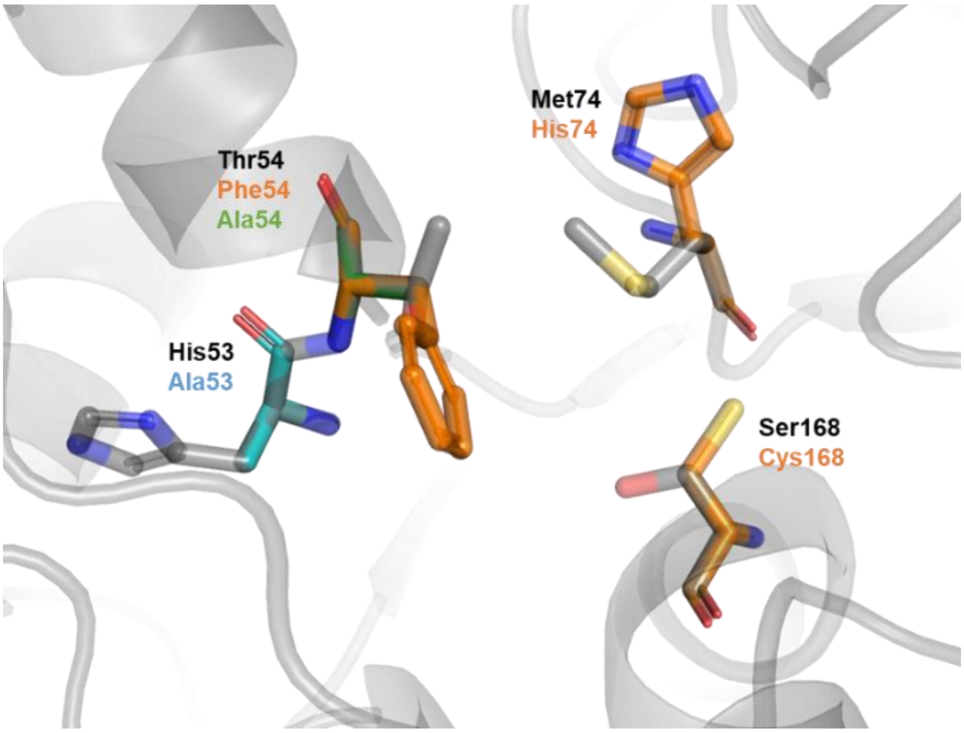

Figure S14. Catharanthus DPAS (*Cr*DPAS) active site with highly conserved residues involved in canonical ADH enzymes with the coordination of the catalytic zinc (Met74 Ser168) and the proton relay (His53, Thr54) that were mutated in this study.

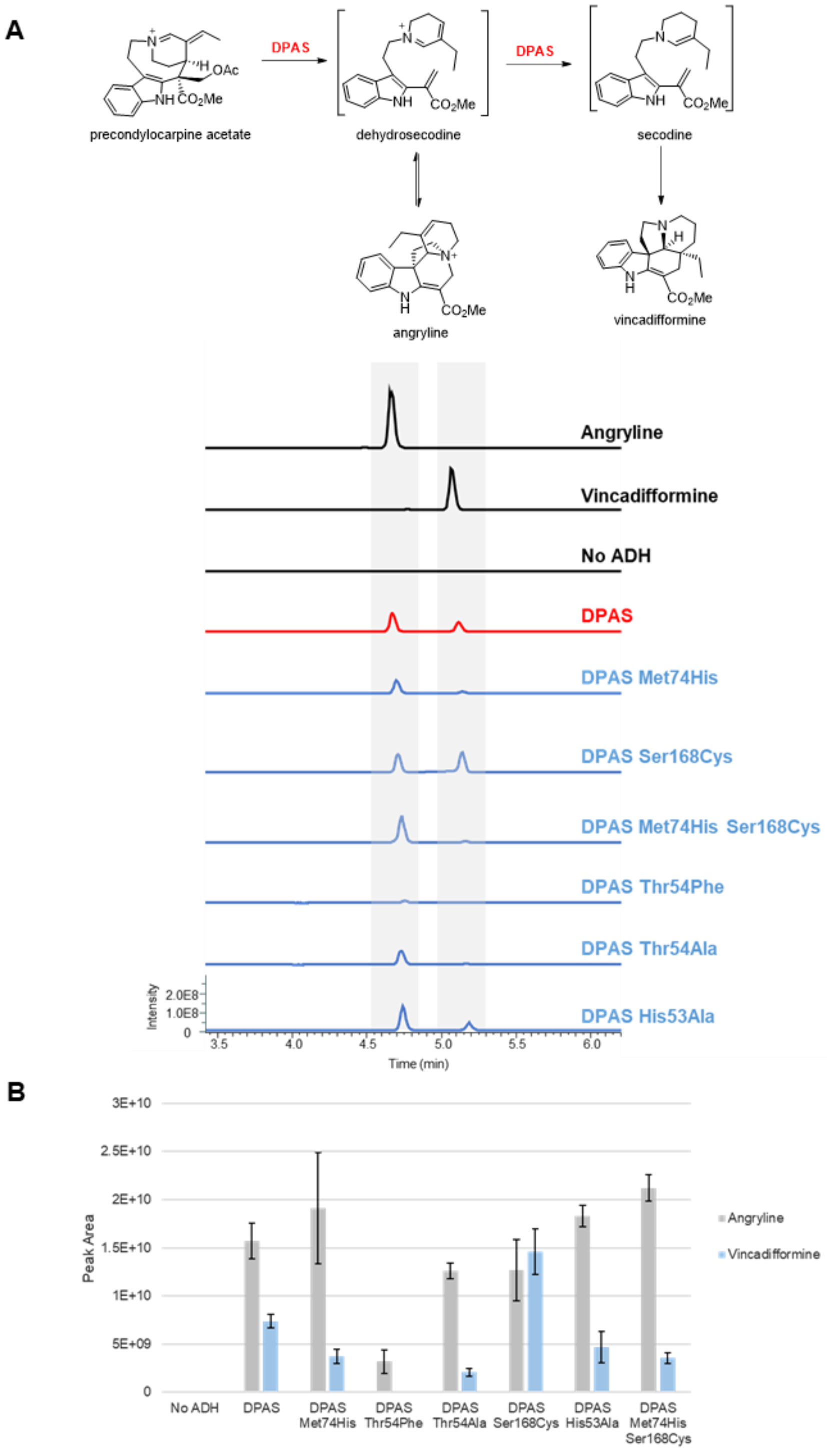

Figure S15. Wild-type *Cr*DPAS and mutants incubated with precondylocarpine acetate showing production of angryline and vincadifformine. **A.** Representative LC-MS chromatogram for wild-type DPAS and mutants. EIC *m/z* 337.05-340.05 **B.** Peak area of angryline and vincadifformine products of DPAS and mutants resulting from an endpoint assay. n=3, bars represent standard deviation.

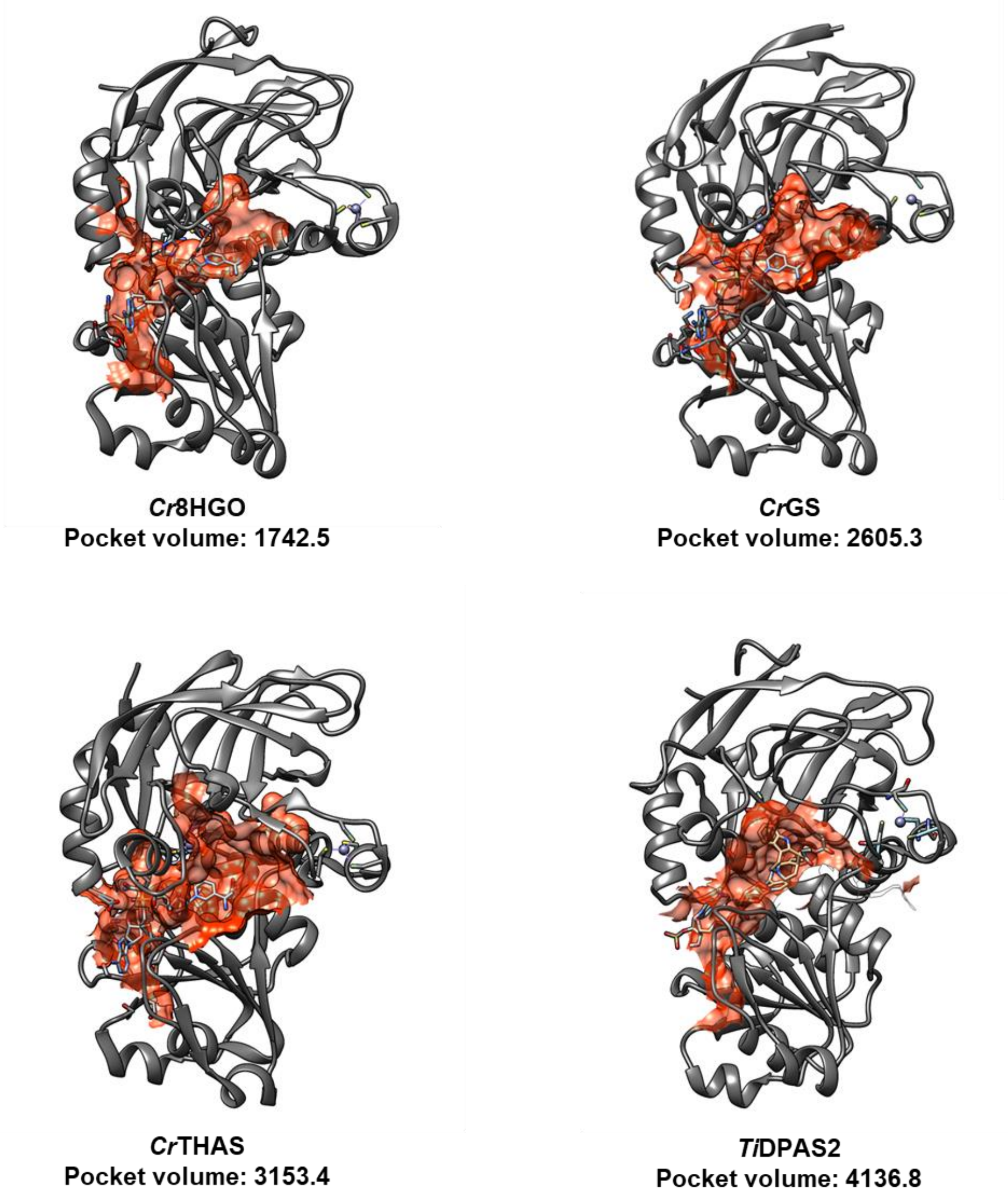

Figure S16. Substrate cavity volume and area of *Cr*8HGO, *Cr*GS, *Cr*THAS and *Ti*DPAS2. Cavity coloured in red, in co-crystallised (8HGO, GS and THAS) or modelled (DPAS2) cofactor $NADP^+$ coloured in grey, and co-crystallised precondylocarpine acetate in DPAS2 coloured in yellow. Pocket volumes computed by CASTp 3.0 [21].

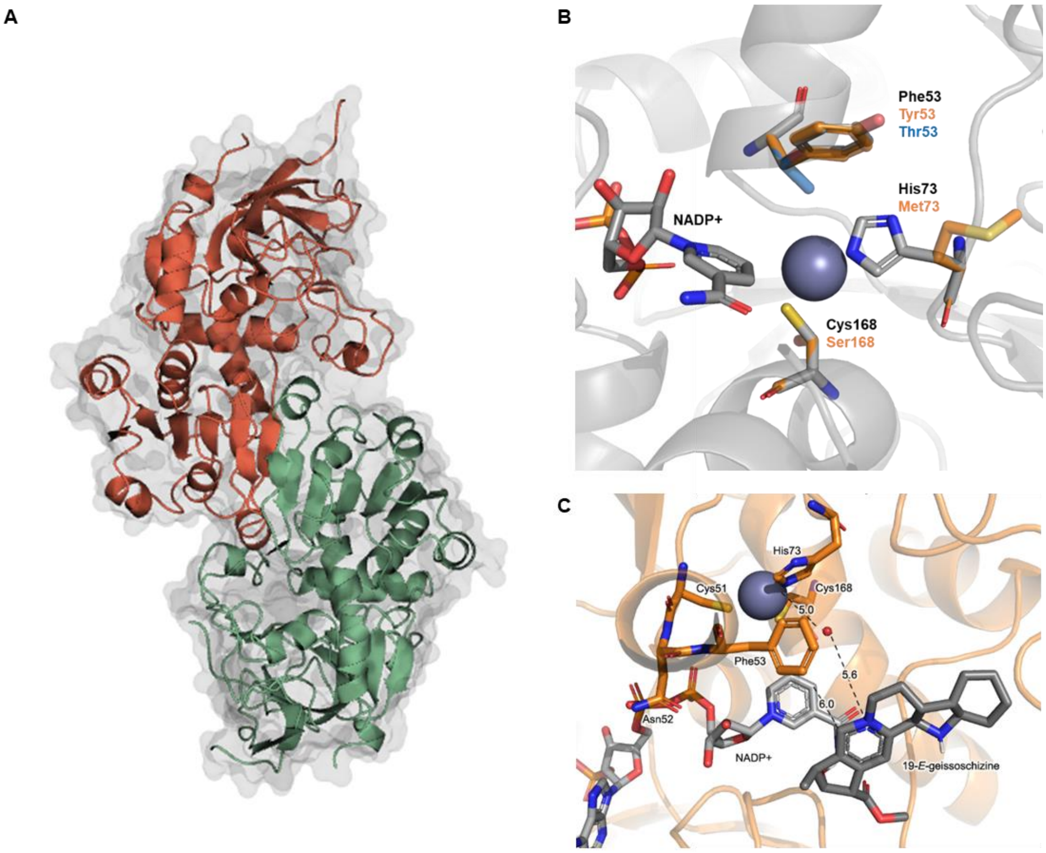

Figure S17. A. *Catharanthus roseus* GS (*Cr*GS) crystallised as a homodimer bound to cofactor NADP+ at 2.00 Å. Structure coloured by chains. **B.** GS active site with residues involved in coordination of the catalytic zinc and proton relay mutated in this study. **C.** NADP+-bound GS active site docked with 19-*E*-geissoschizine. Distance between the 4-pro-*R* -hydride of NADPH and position of reduction, and the distance between the catalytic zinc, bound water molecule and the substrate iminium as in proposed enzyme mechanism are shown.

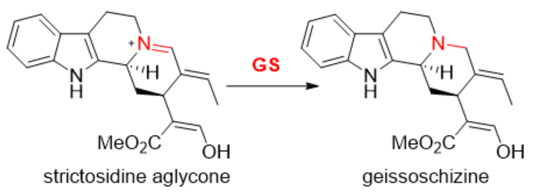

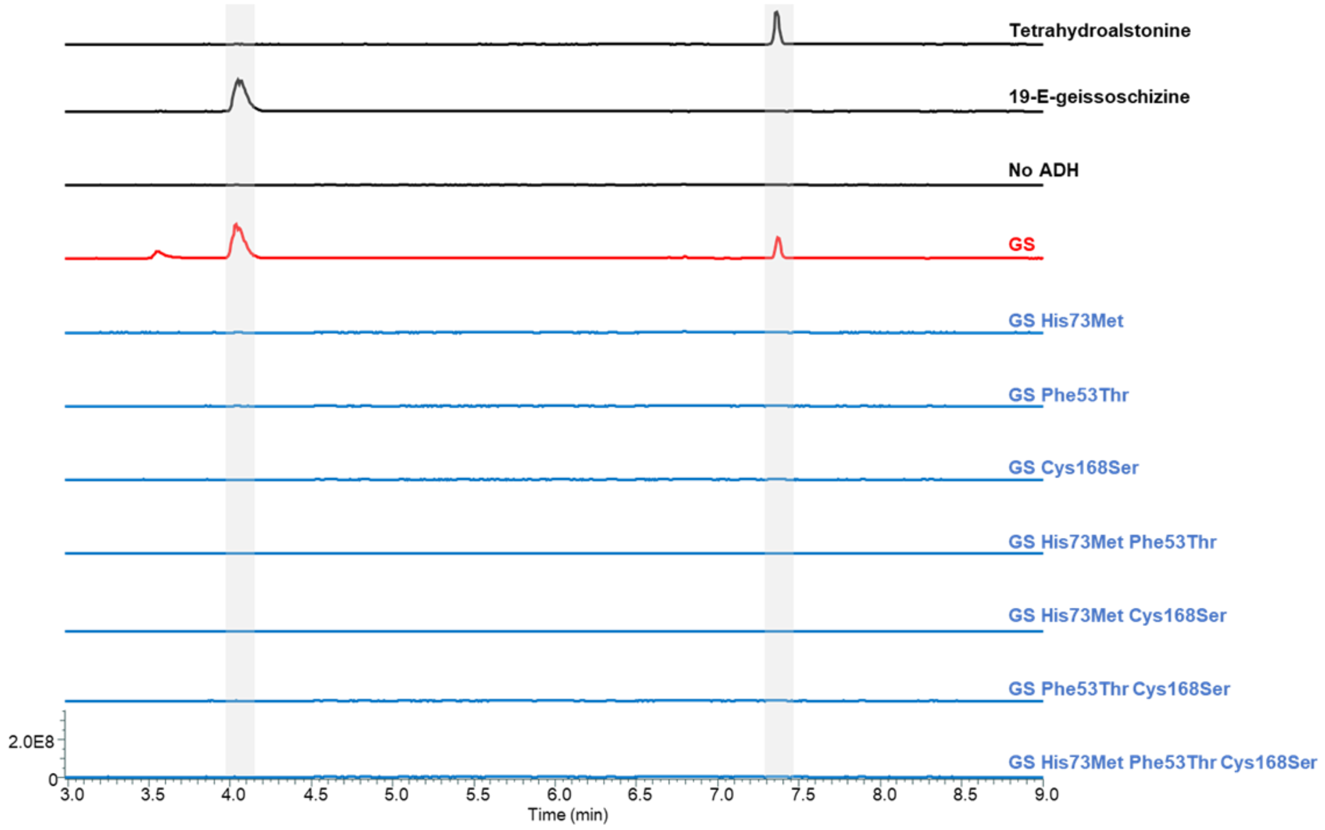

Figure S18. LC-MS chromatograms of *Cr*GS and mutants reacted with substrate strictosidine aglycone and cofactor NADPH. These mutants probe the role of residues involved in coordination of the catalytic zinc and involved in the proton relay. EIC *m/z* 353.185-353.225.

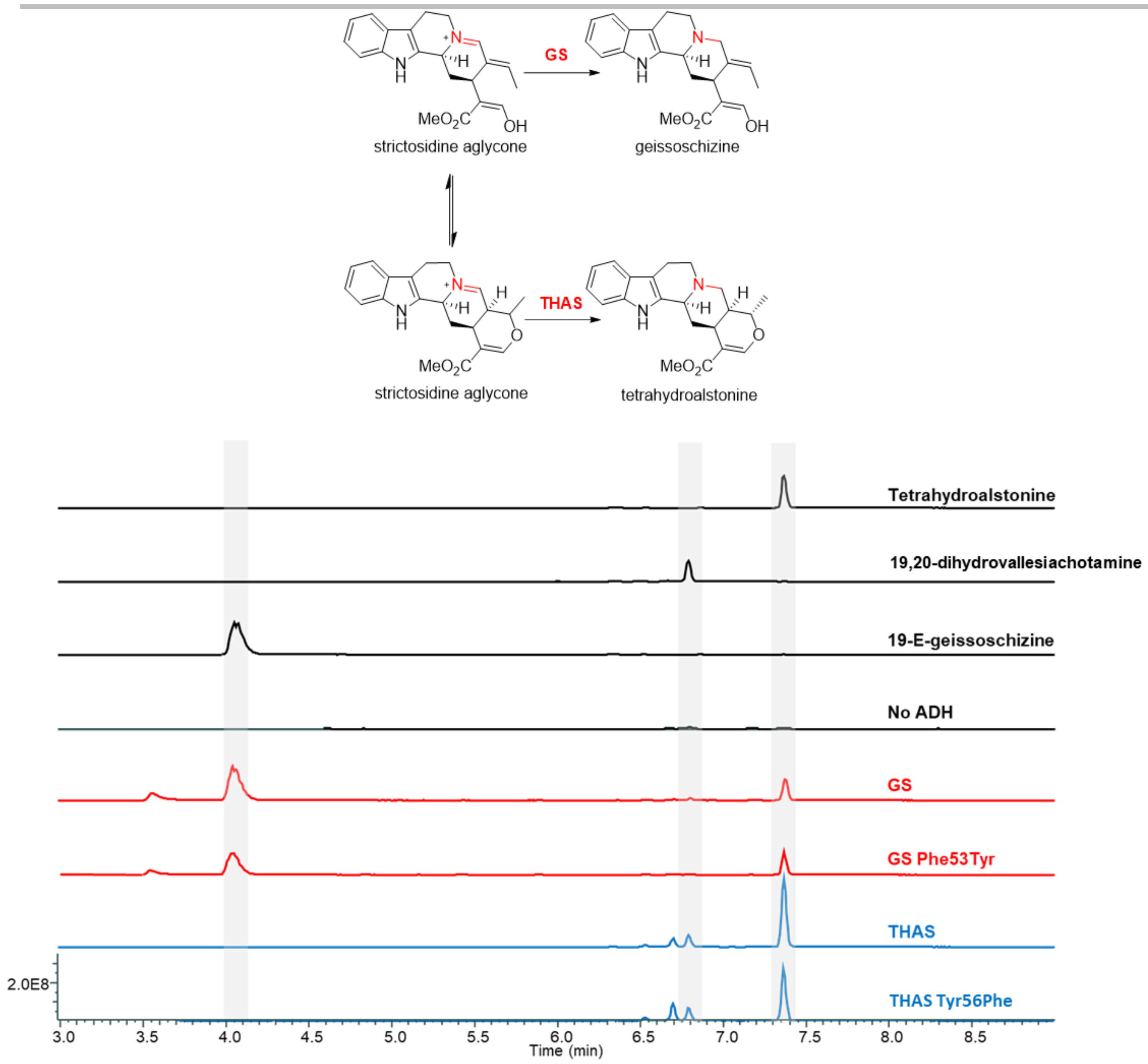

Figure S19. LC-MS chromatograms of *Cr*GS and *Cr*GS Phe53Tyr mutant and the corresponding *Cr*THAS and *Cr*THAS Tyr56Phe mutant reacted with substrate strictosidine aglycone and cofactor NADPH. These mutations probe the role of the hydroxyl group in the proton relay. EIC *m/z* 353.185-353.225.

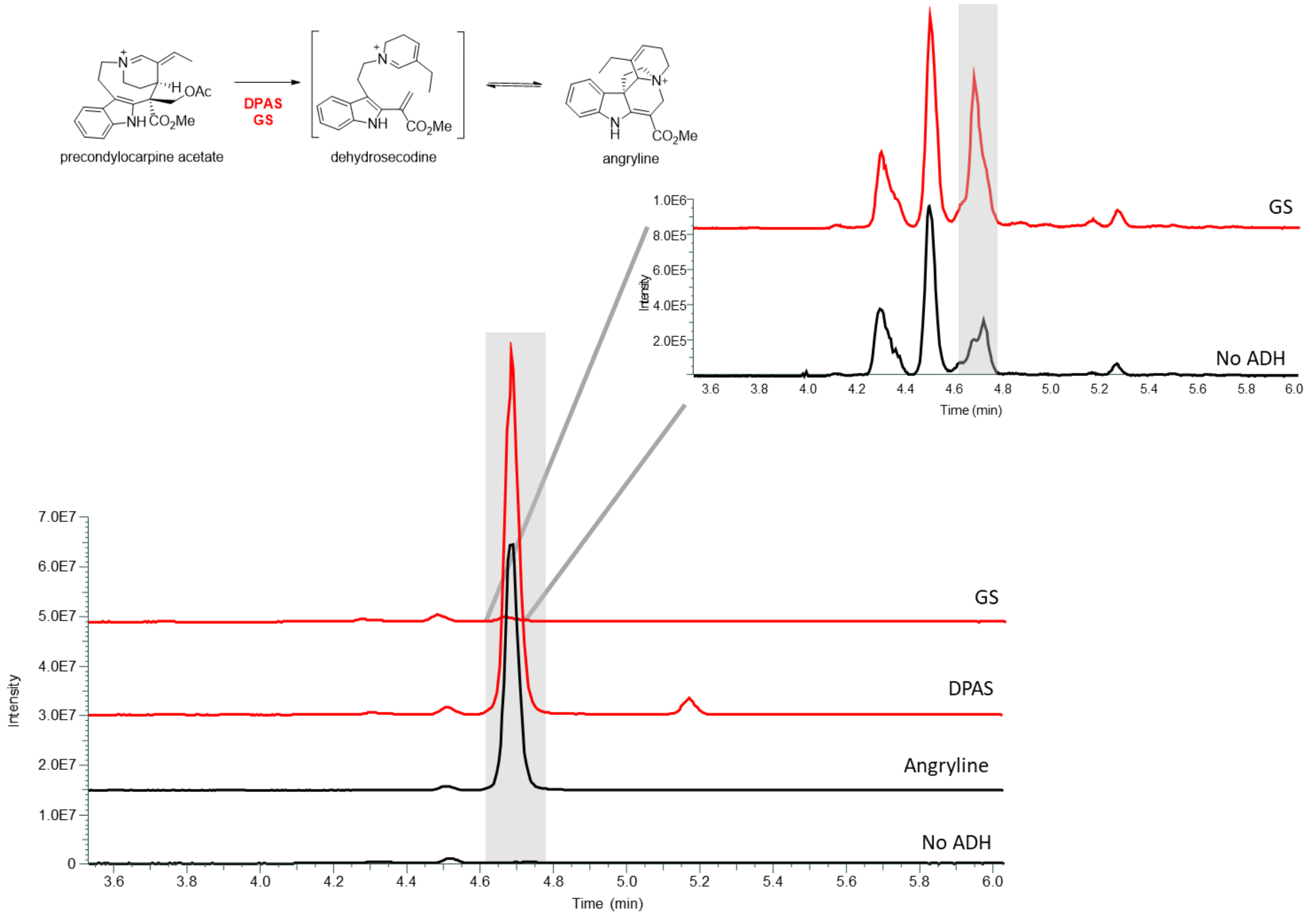

Figure S20. LC-MS chromatograms of *Cr*DPAS and CrGS reacted with substrate precondylocarpine acetate and cofactor NADPH incubated for 1 hour at 30 °C. EIC *m/z* 337.180-337.200. Inset of GS reaction to show small amount of peak with same mass and elution time as angryline standard.

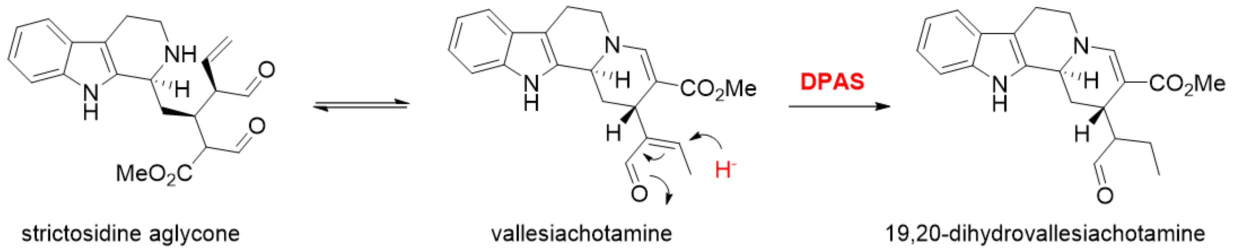

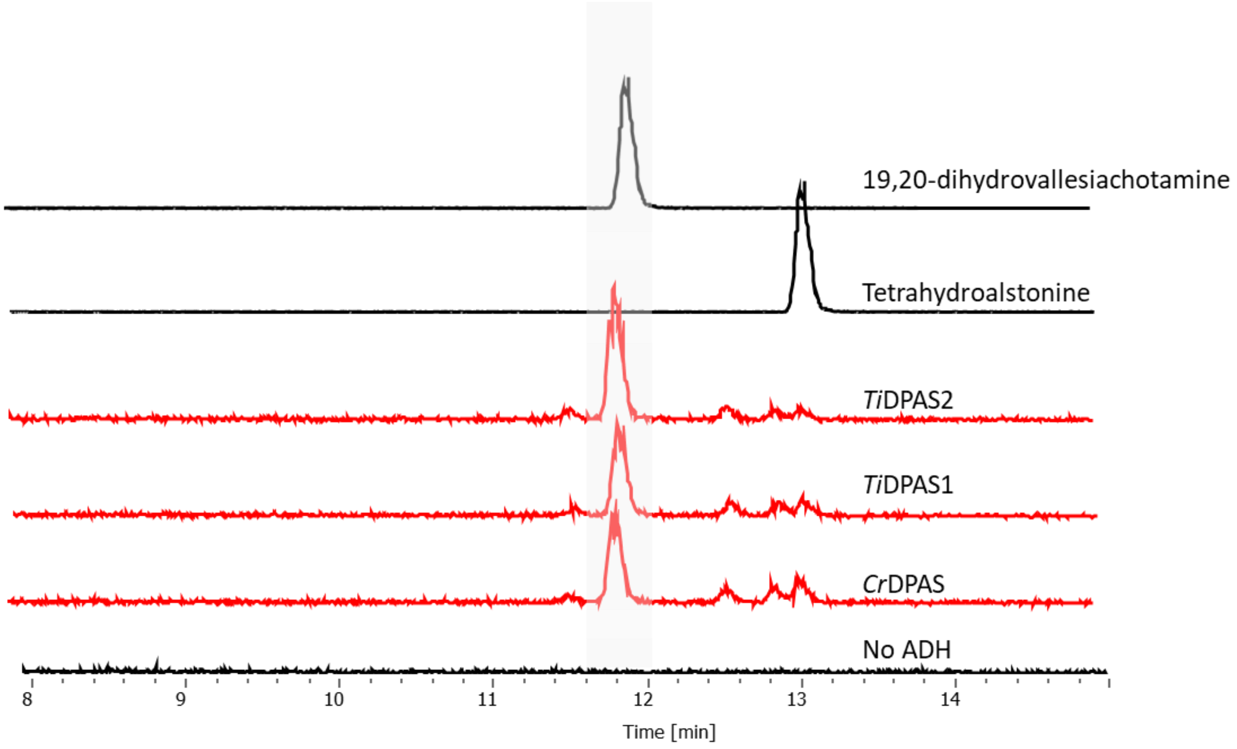

Figure S21. LC-MS chromatograms of *Cr*DPAS, *Ti*DPAS1 and *Ti*DPAS2 reacted with substrate strictosidine aglycone and cofactor NADPH. EIC *m/z* 353.185-353.225.

A

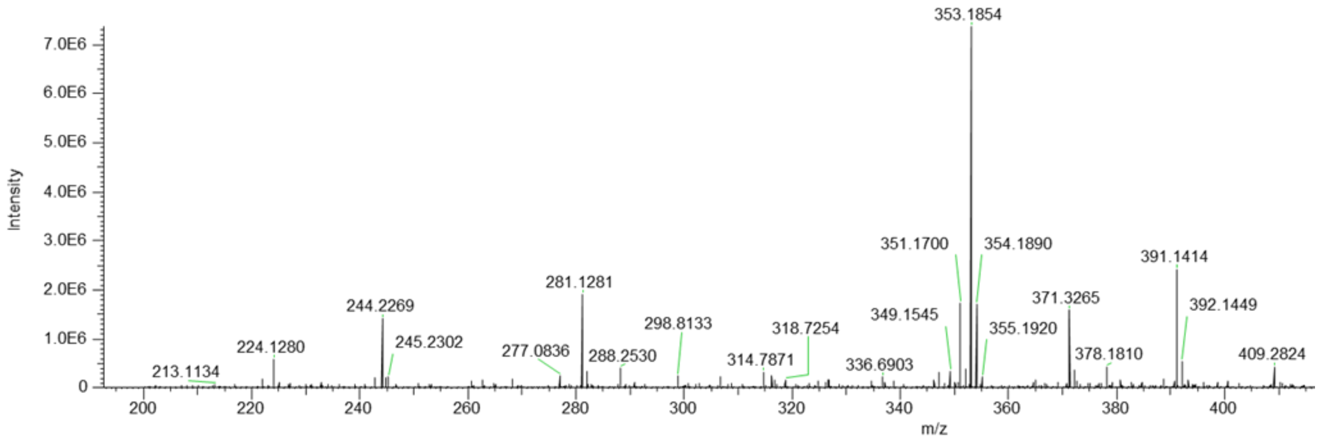

B

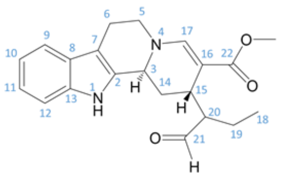

19,20-dihydrovallesiachotamine

^{1}H 700 MHz, MeOH-d_3, 25 °C

pos.	δ_H	mult.	J_{HH}	δ_C
1	10.4	*bs*	-	-
2	-	-	-	133.9
3α	4.55	*bd*	12.0	49.9
4	-	-	-	-
5α	3.61	*ddd*	13.0/12.4/3.9	52.2
5β	3.81	*dd*	13.0/5.4	52.2
6α	2.76	*m*	-	22.9
6β	2.86	*m*	-	22.9
7	-	-	-	108.2
8	-	-	-	127.9
9	7.39	*bd*	7.8	118.6
10	6.98	*dd*	7.8/7.2	119.8
11	7.06	*dd*	8.0/7.2	122.3
12	7.28	*bd*	8.0	112.0
13	-	-	-	138.3
14α	2.50	*ddd*	13.8/4.0/2.0	32.5
14β	1.60	*ddd*	13.8/12.0/5.0	32.5
15β	3.18	*ddd*	7.5/5.0/2.0	31.6
16	-	-	-	94.1
17	7.74	*s*	-	148.9
18	0.93	*t*	7.4	12.5
19a	1.82	*ddt*	14.0/9.6/7.4	20.8
19b	1.54	*m*	-	20.8
20	2.42	*m*	-	60.2
21	9.72	*d*	3.4	208.0
22	-	-	-	170.9
OMe	3.67	*s*	-	51.1

Multiplicity abbreviations: s, singlet; d, doublet; t, triplet; m, multiplet; bs, broad signlet; bd, broad doublet; dd, doublet of doublets; bdd, broad doublet of doublets; ddd, doublet of doublet of doublets; ddt, doublet of doublet of triplets.

Figure S22. MS/MS and NMR data of 19,20-dihydrovallesiachotamine. **A.** MS/MS spectra of 19,20-dehydrovallesiachotamine. Formula: $C_{21}H_{24}N_2O_3$; observed mass: 353.1854; theoretical mass: 353.1860; error 1.6988 p.p.m. **B.** ^{1}H NMR spectra for 19,20-dihydrovallesiachotamine in MeOH-d_3.

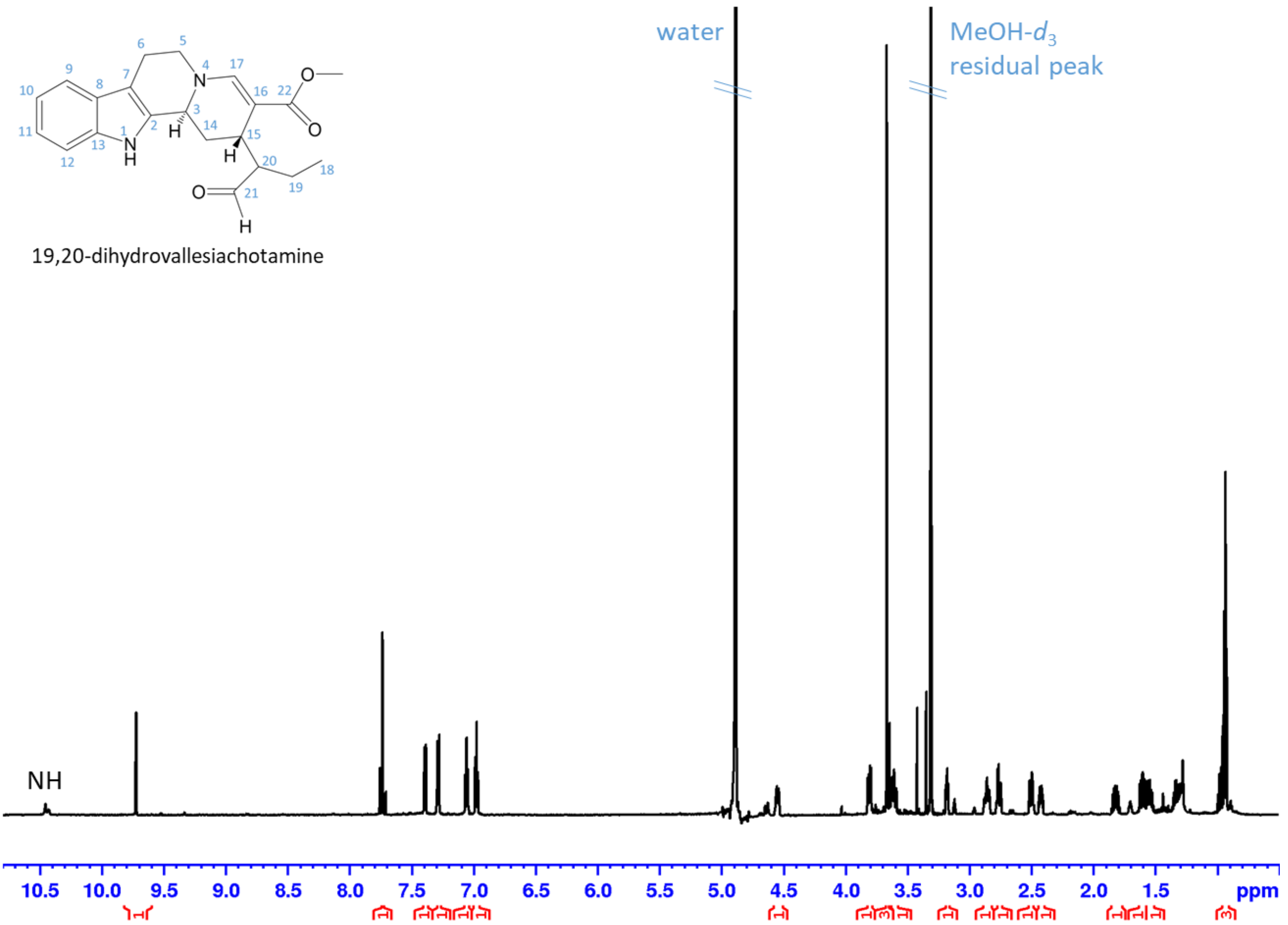

Figure S23. ^{1}H NMR data of 19,20-dihydrovallesiachotamine with water suppression, full range in MeOH-d_3

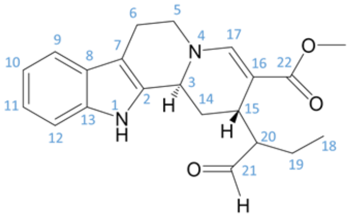

19,20-dihydrovallesiachotamine

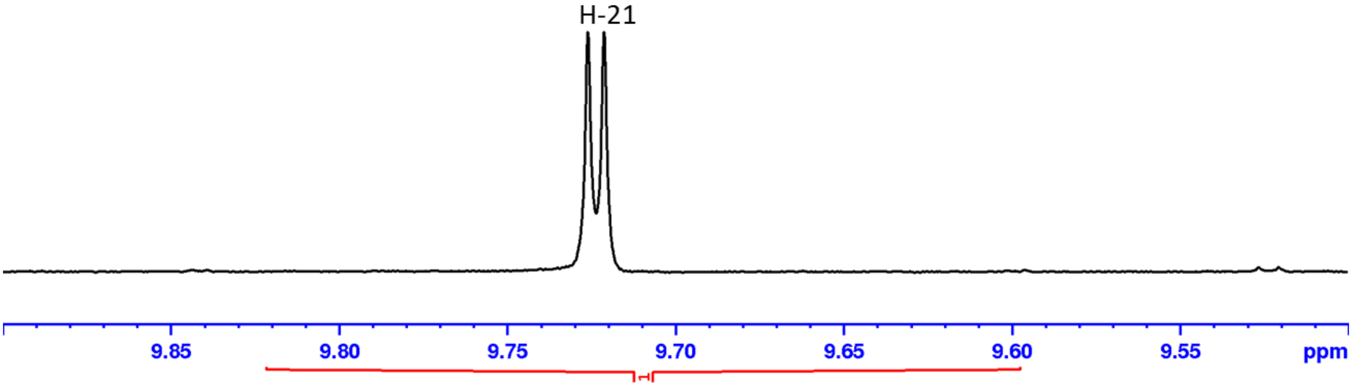

Figure S24. ^{1}H NMR data of 19,20-dihydrovallesiachotamine with water suppression, aldehyde range in MeOH-d_3

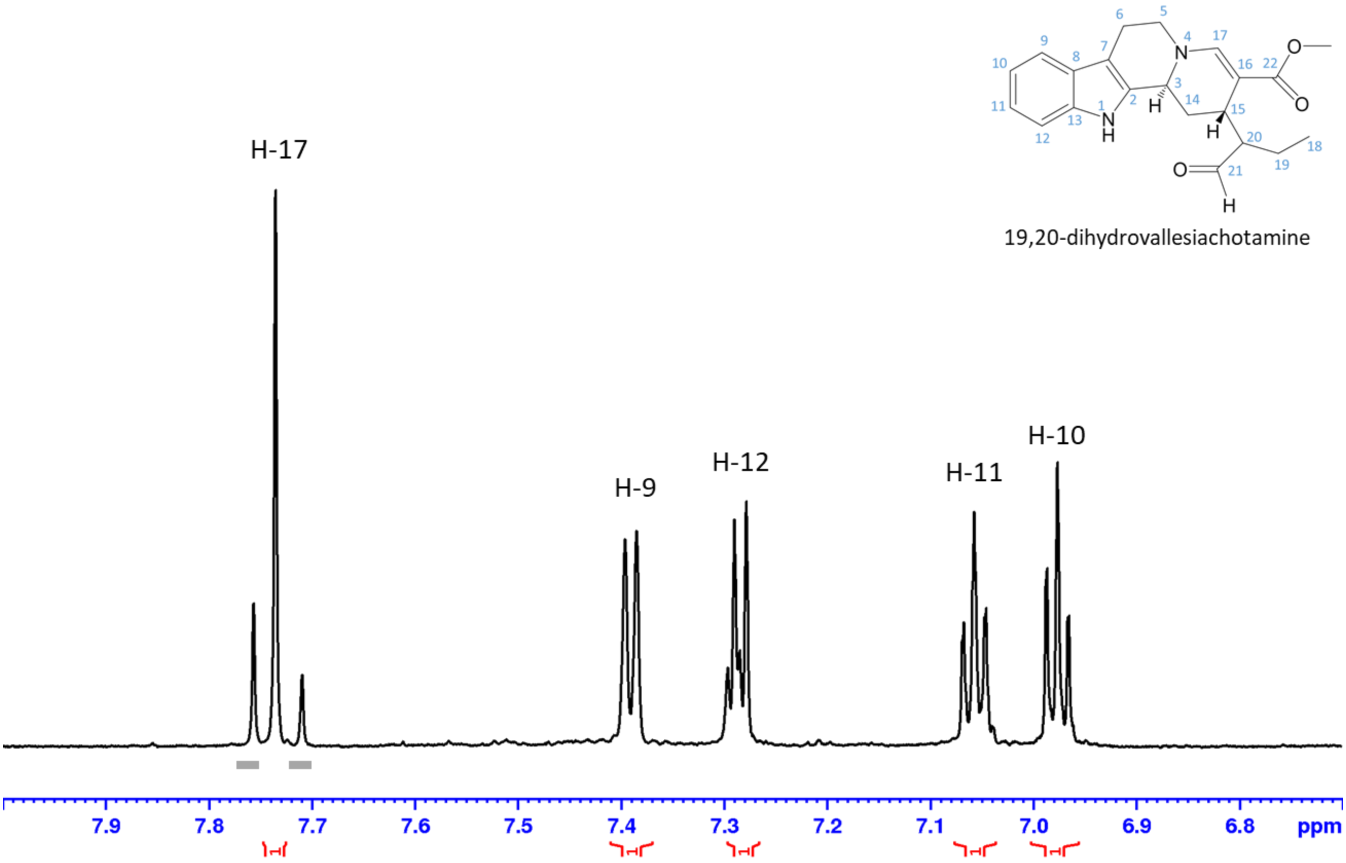

Figure S25. ^{1}H NMR data of 19,20-dihydrovallesiachotamine with water suppression, aromatic range in MeOH-d_3. Grey bars indicate impurities.

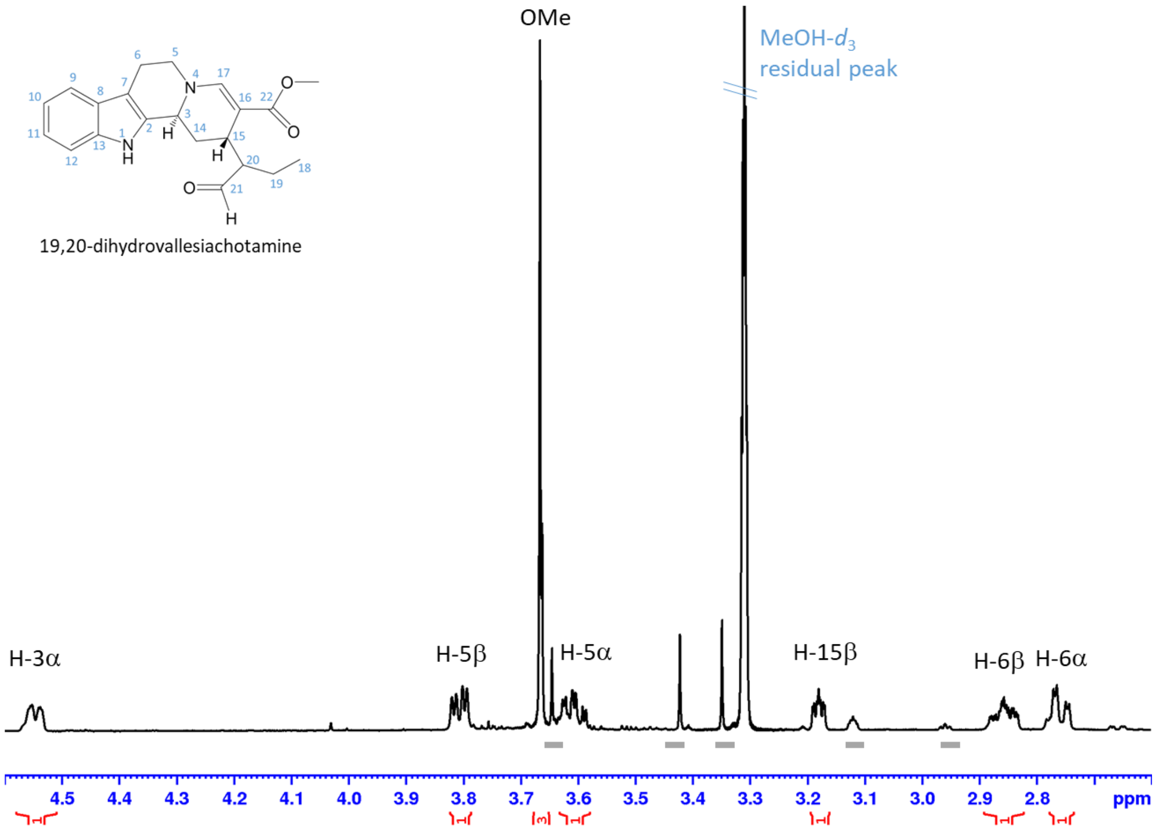

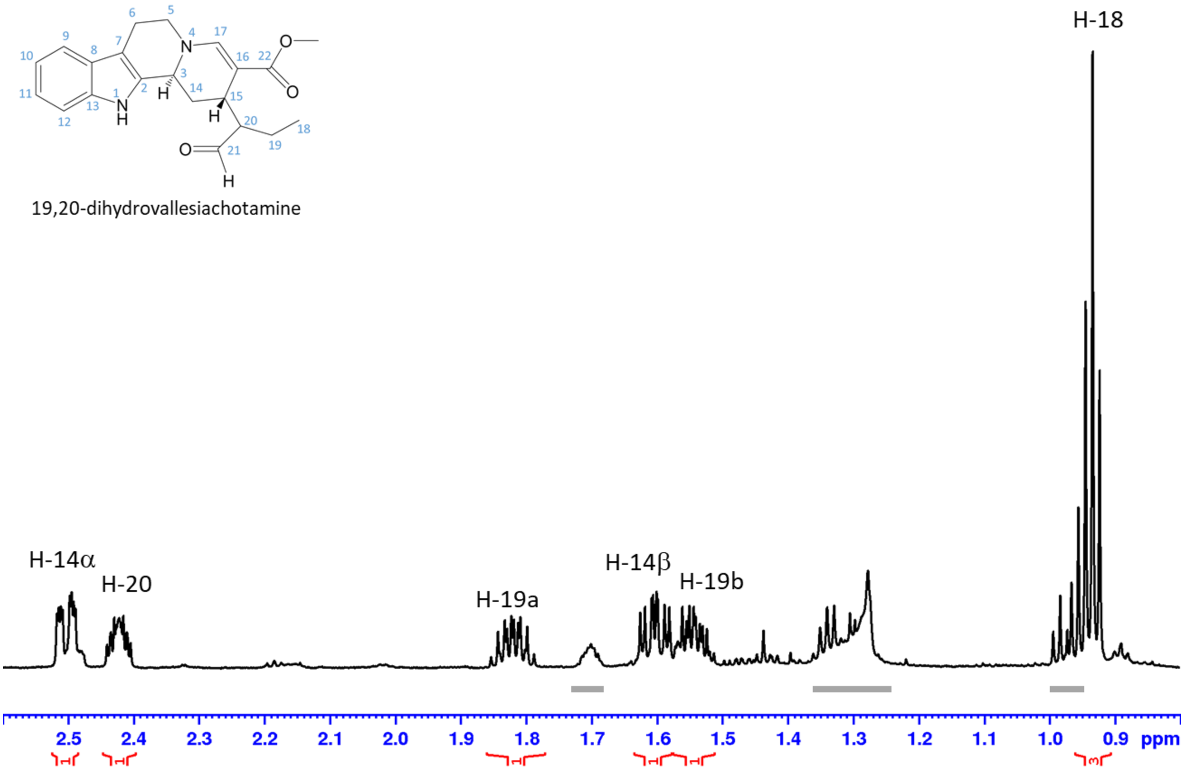

Figure S26. ^{1}H NMR data of 19,20-dihydrovallesiachotamine with water suppression, aliphatic range in MeOH-d_3. Grey bars indicate impurities.

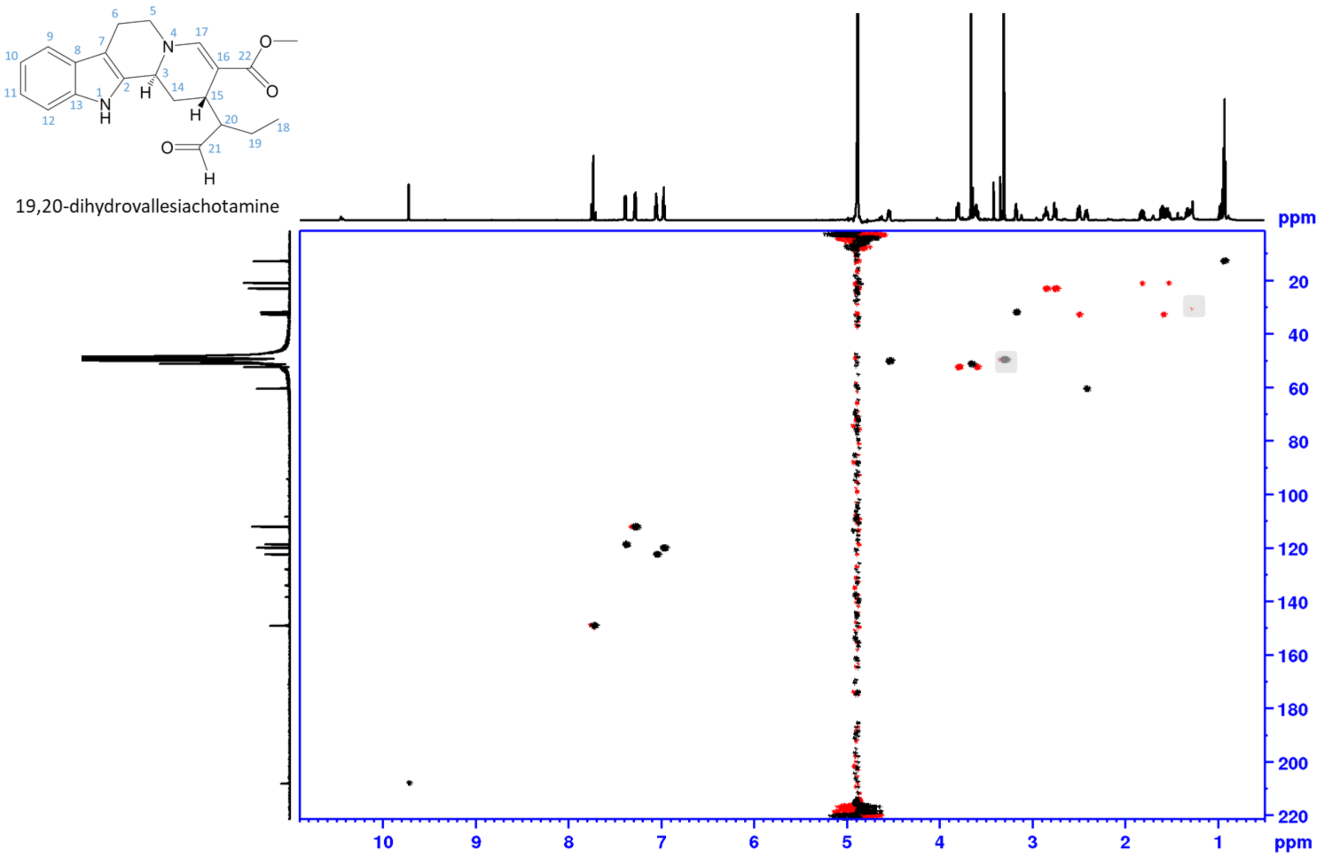

Figure S27. NMR data of 19,20-dihydrovallesiachotamine, phase sensitive HSQC, full range in MeOH-d_3. Shaded areas mark impurity and solvent, red: CH2, black: CH, CH3

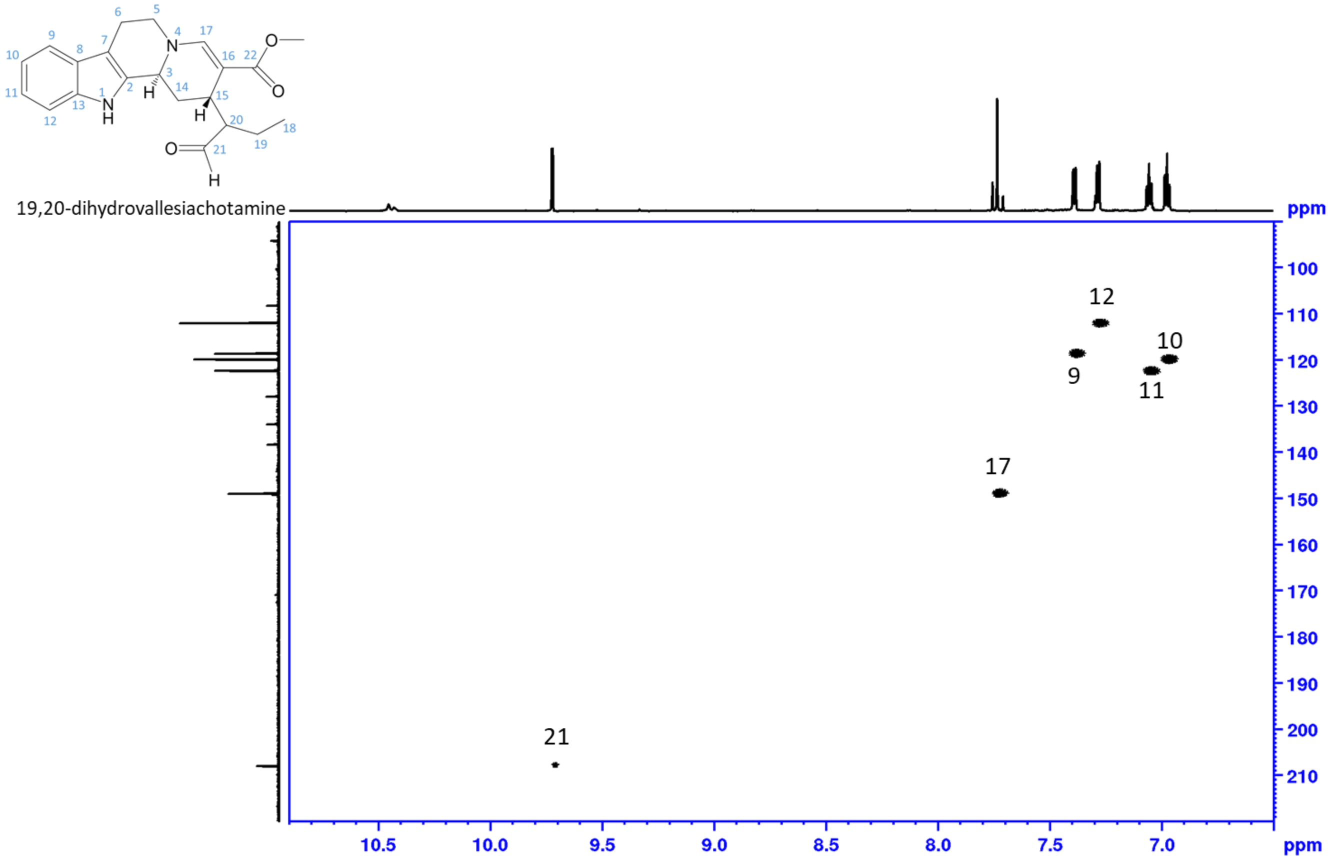

Figure S28. NMR data of 19,20-dihydrovallesiachotamine, phase sensitive HSQC, aldehyde and aromatic range in MeOH-d_3. Shaded areas mark impurity and solvent, red: CH2, black: CH

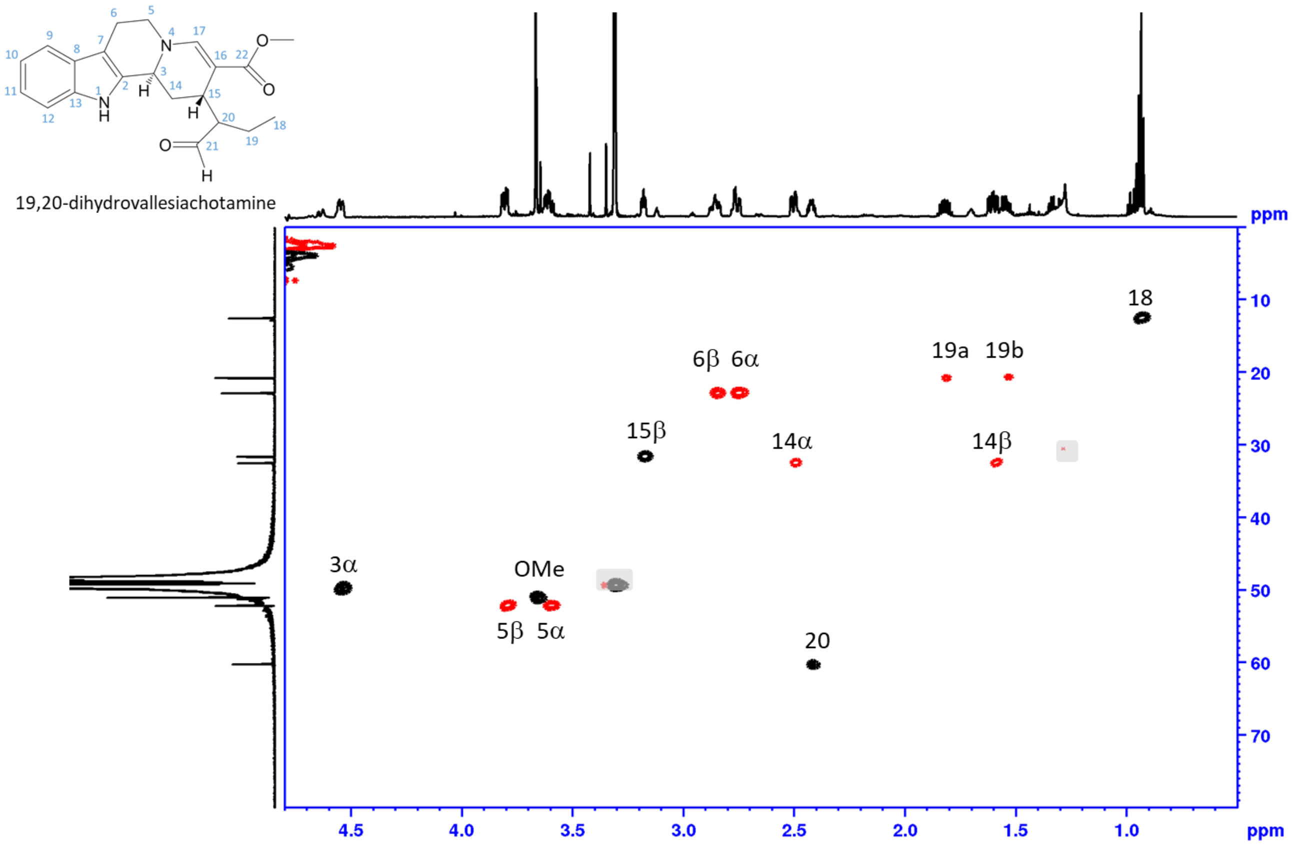

Figure S29. NMR data of 19,20-dihydrovallesiachotamine, phase sensitive HSQC, aliphatic range in MeOH-d_3. Shaded areas mark impurity and solvent, red: CH2, black: CH

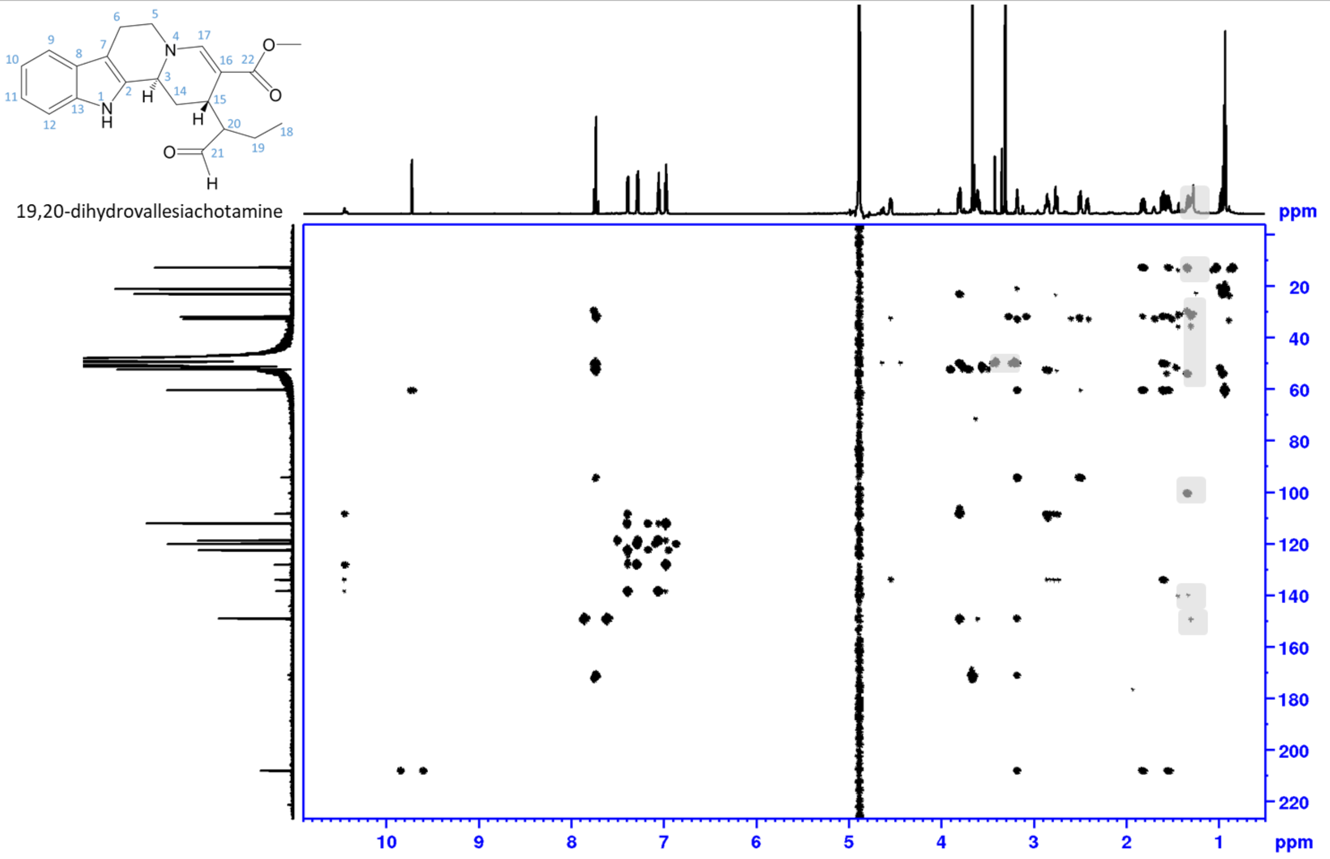

Figure S30. NMR data of 19,20-dihydrovallesiachotamine, HMBC, full range in MeOH-d_3. Shaded areas mark impurity and solvent.

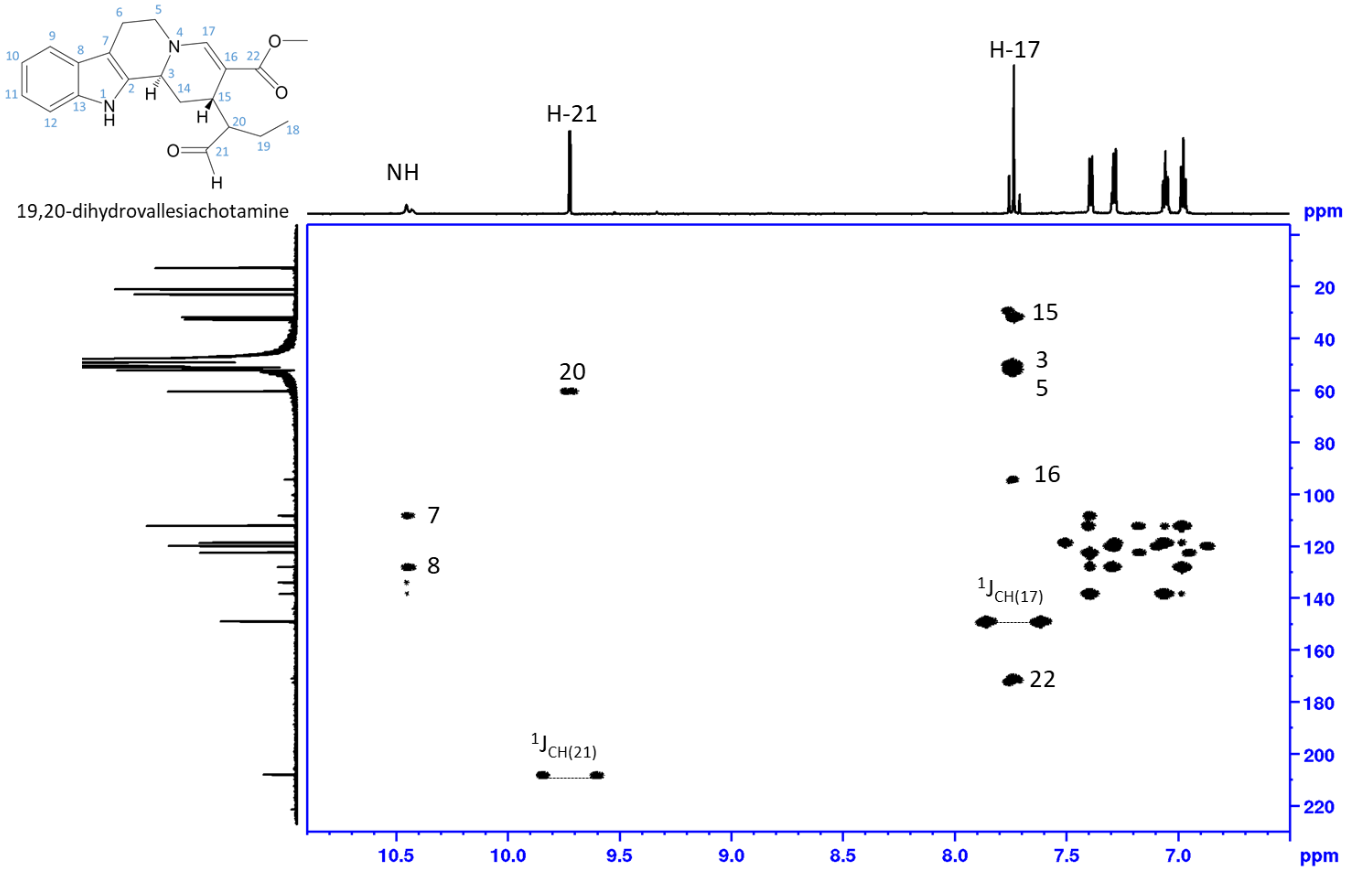

Figure S31. NMR data of 19,20-dihydrovallesiachotamine, HMBC, aldehyde and aromatic range in MeOH-d_3. Shaded areas mark impurity and solvent.

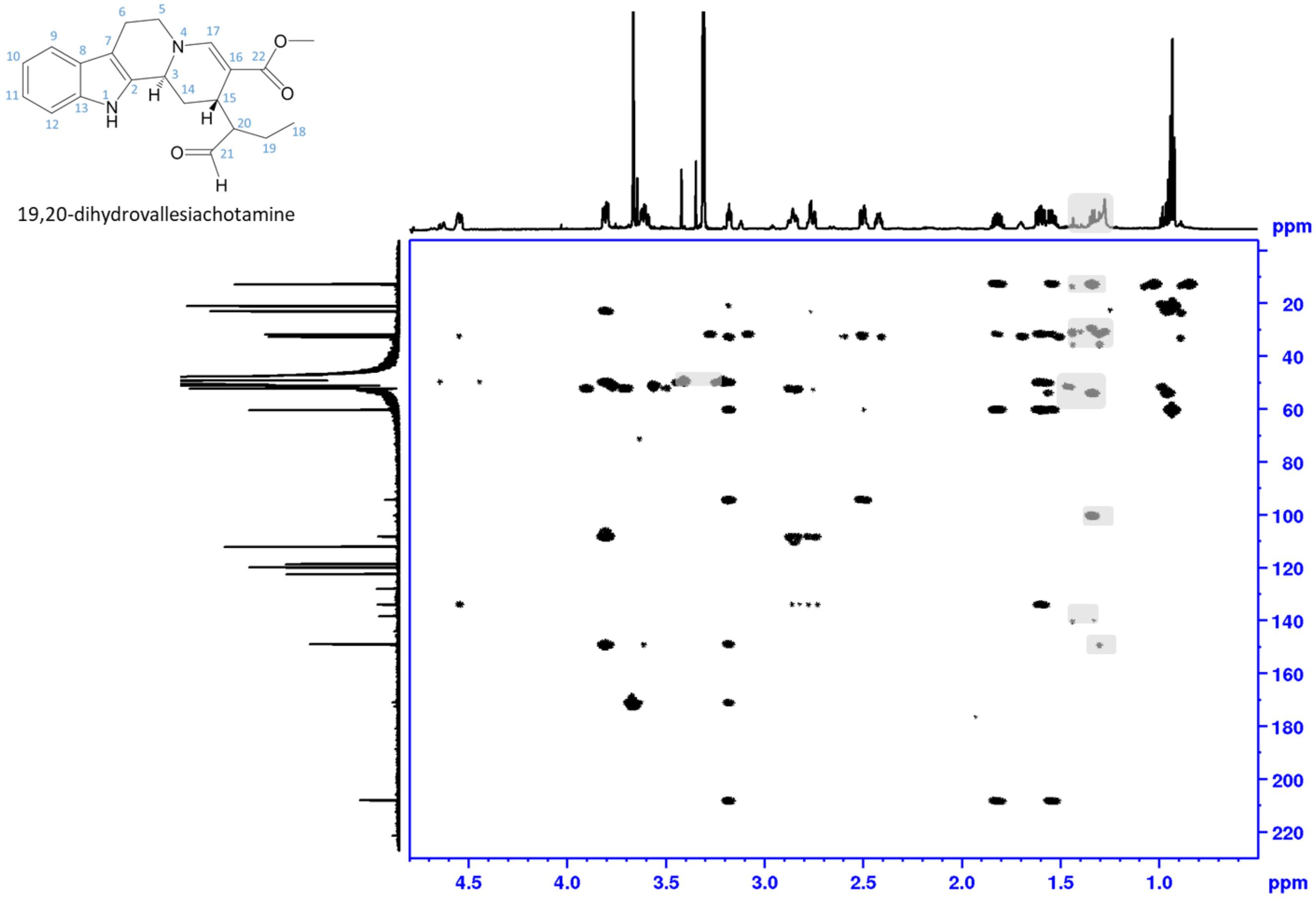

Figure S32. NMR data of 19,20-dihydrovallesiachotamine, HMBC, aliphatic range in MeOH-d_3. Shaded areas mark impurity and solvent.

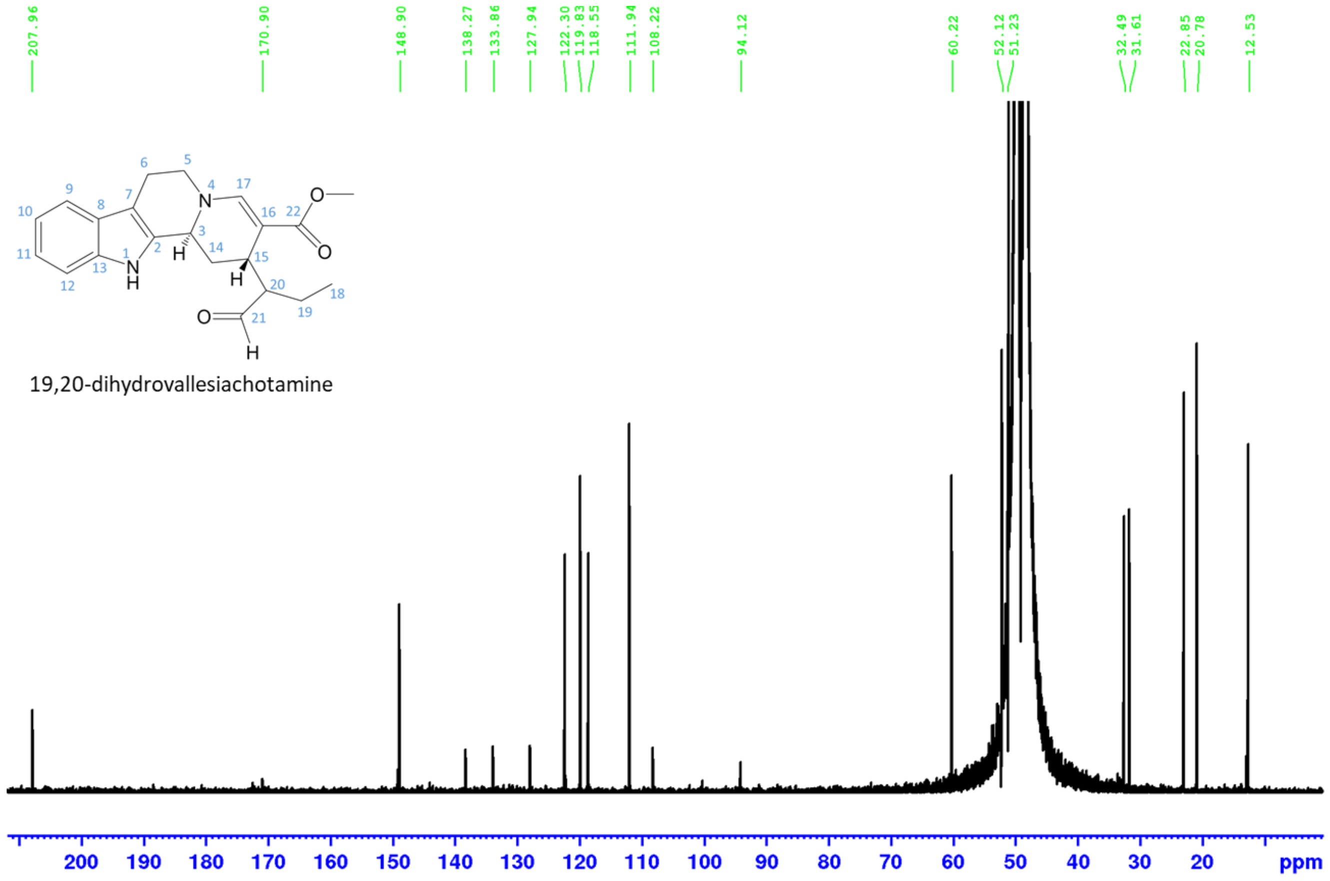

Figure S33. NMR data of 19,20-dihydrovallesiachotamine, DEPTQ, power spectrum, full range in MeOH-d_3.

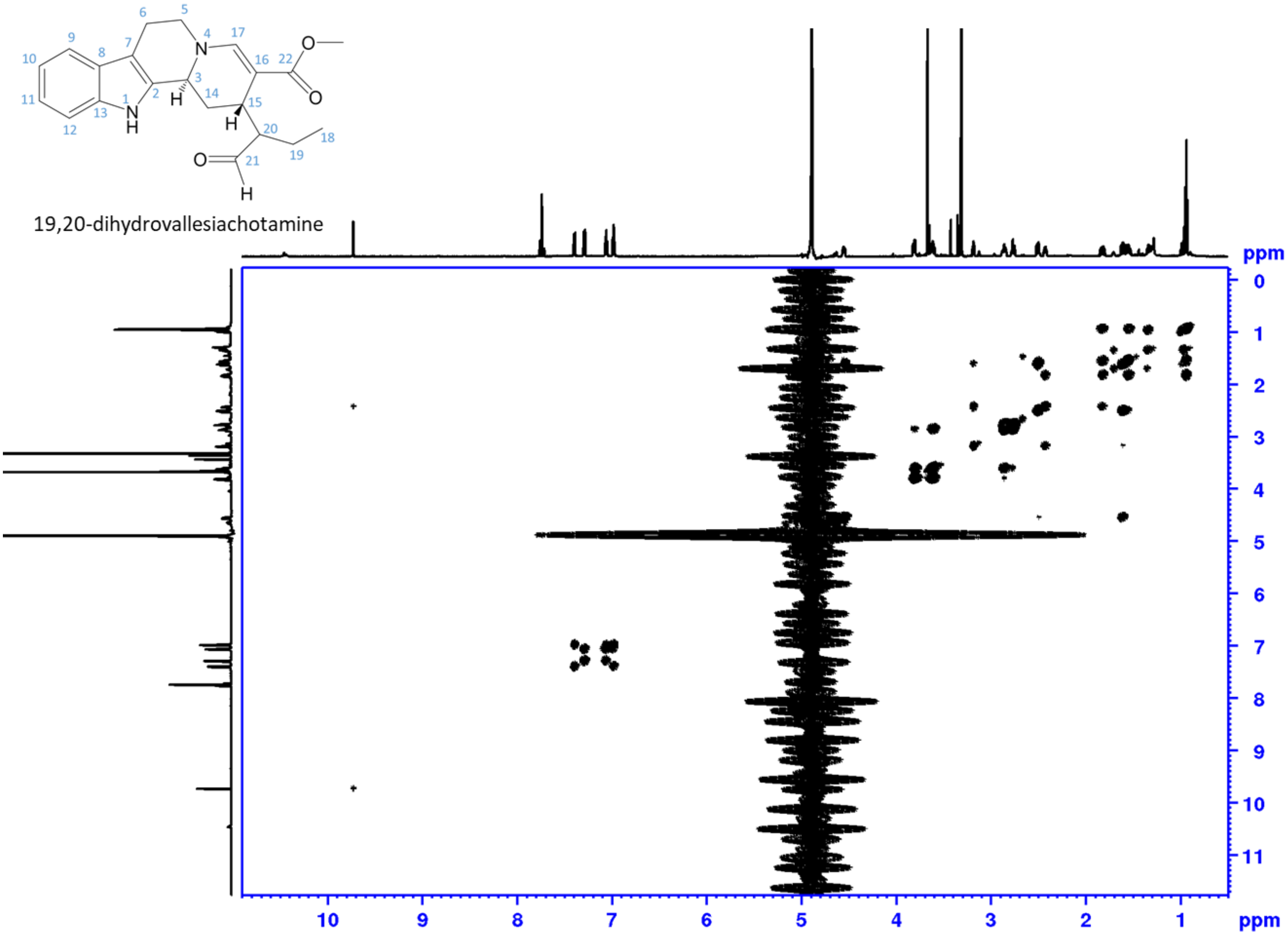

Figure S34. NMR data of 19,20-dihydrovallesiachotamine, 1H-1H DQF COSY with water suppression, magnitude mode processed, full range in MeOH-d_3.

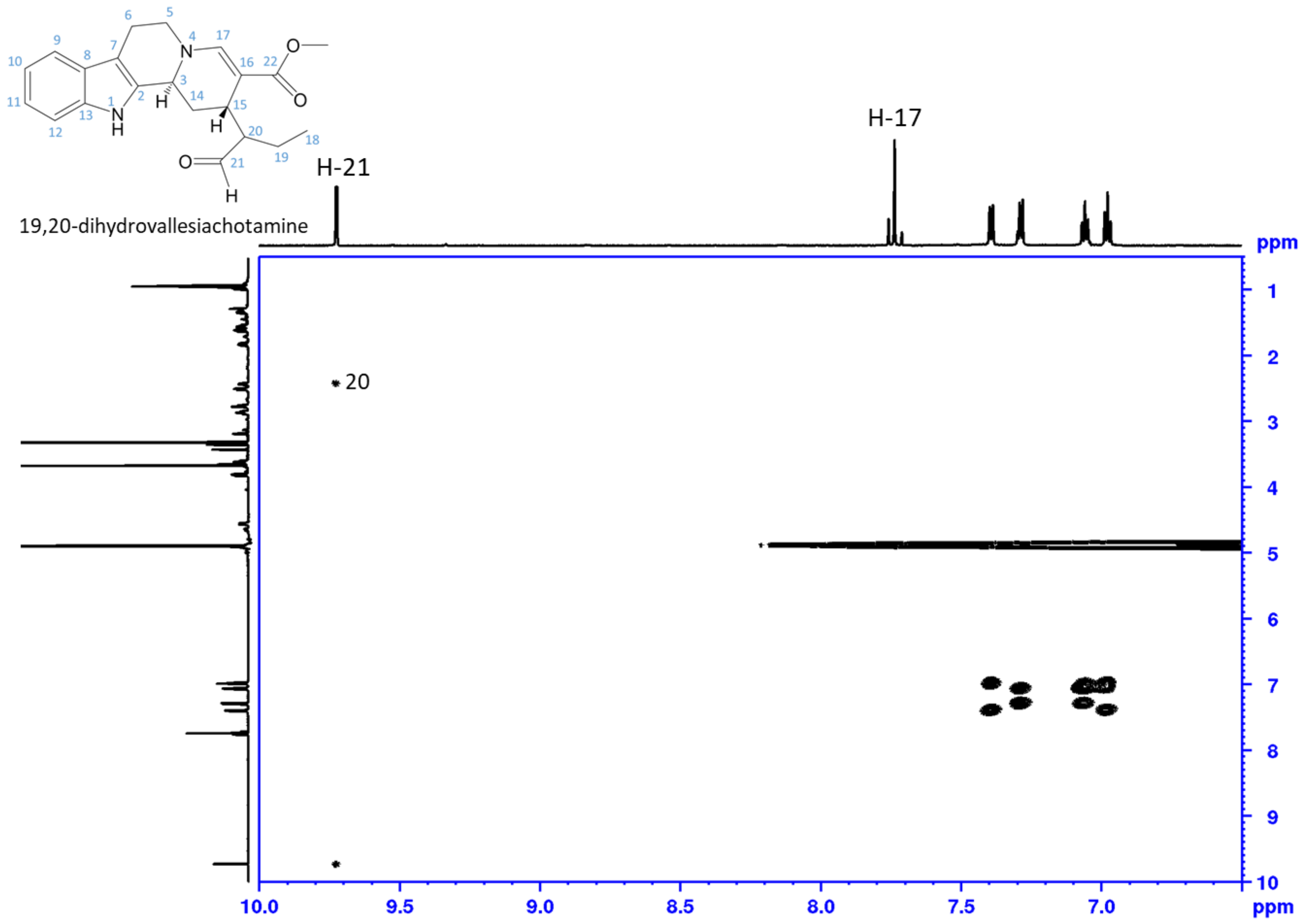

Figure S35. NMR data of 19,20-dihydrovallesiachotamine, 1H-1H DQF COSY with water suppression, magnitude mode processed, aldehyde and aromatic range in MeOH-d_3.

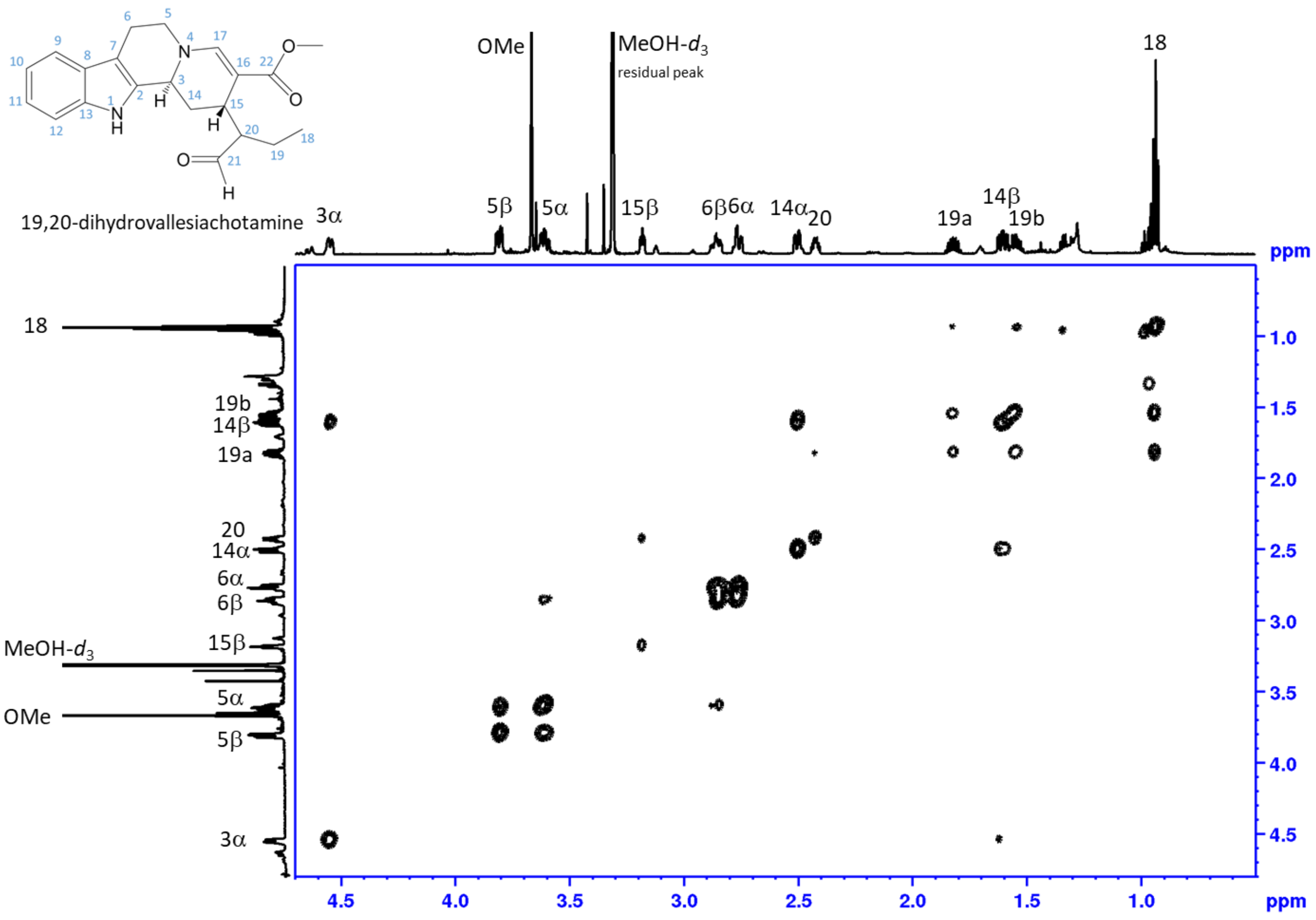

Figure S36. NMR data of 19,20-dihydrovallesiachotamine, 1H-1H DQF COSY with water suppression, magnitude mode processed, aliphatic range in MeOH-*d*3.

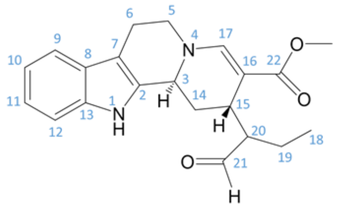

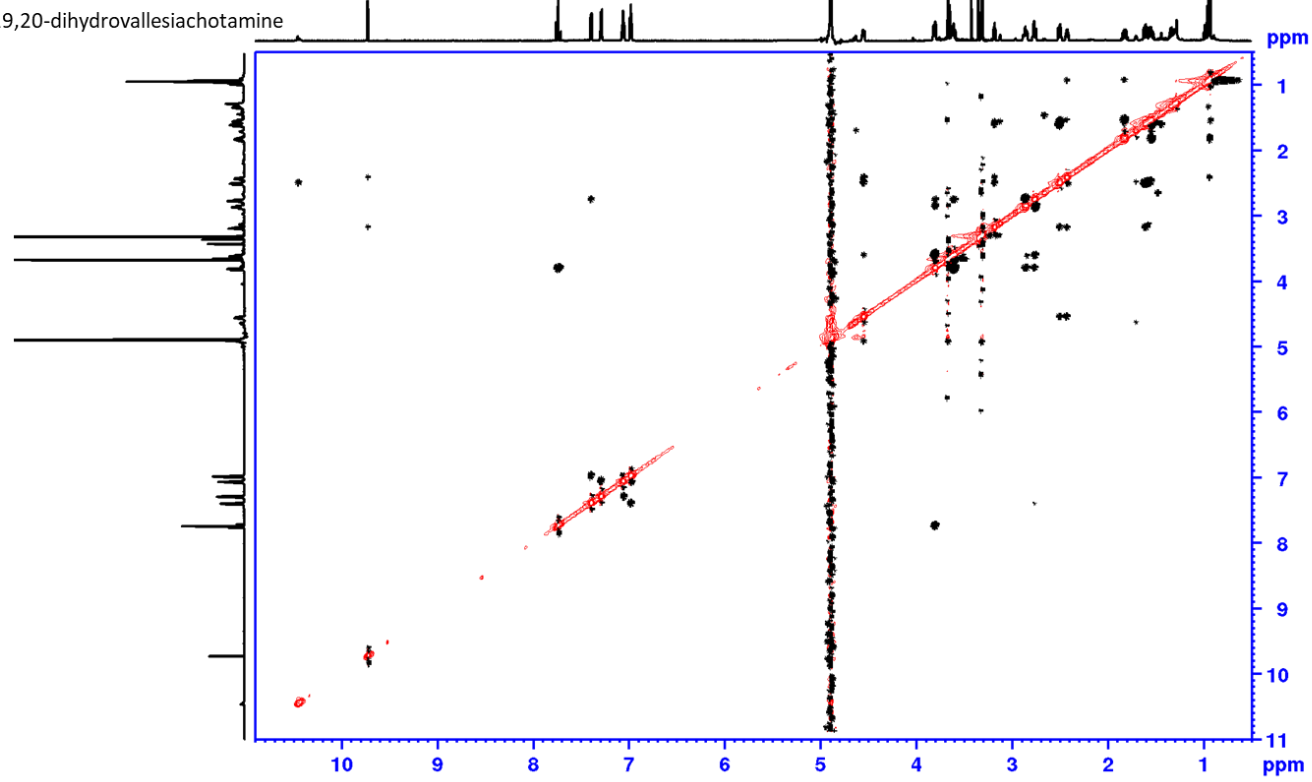

Figure S37. NMR data of 19,20-dihydrovallesiachotamine, ^{1}H-^{1}H ROESY with water suppression, full range in MeOH-d_3

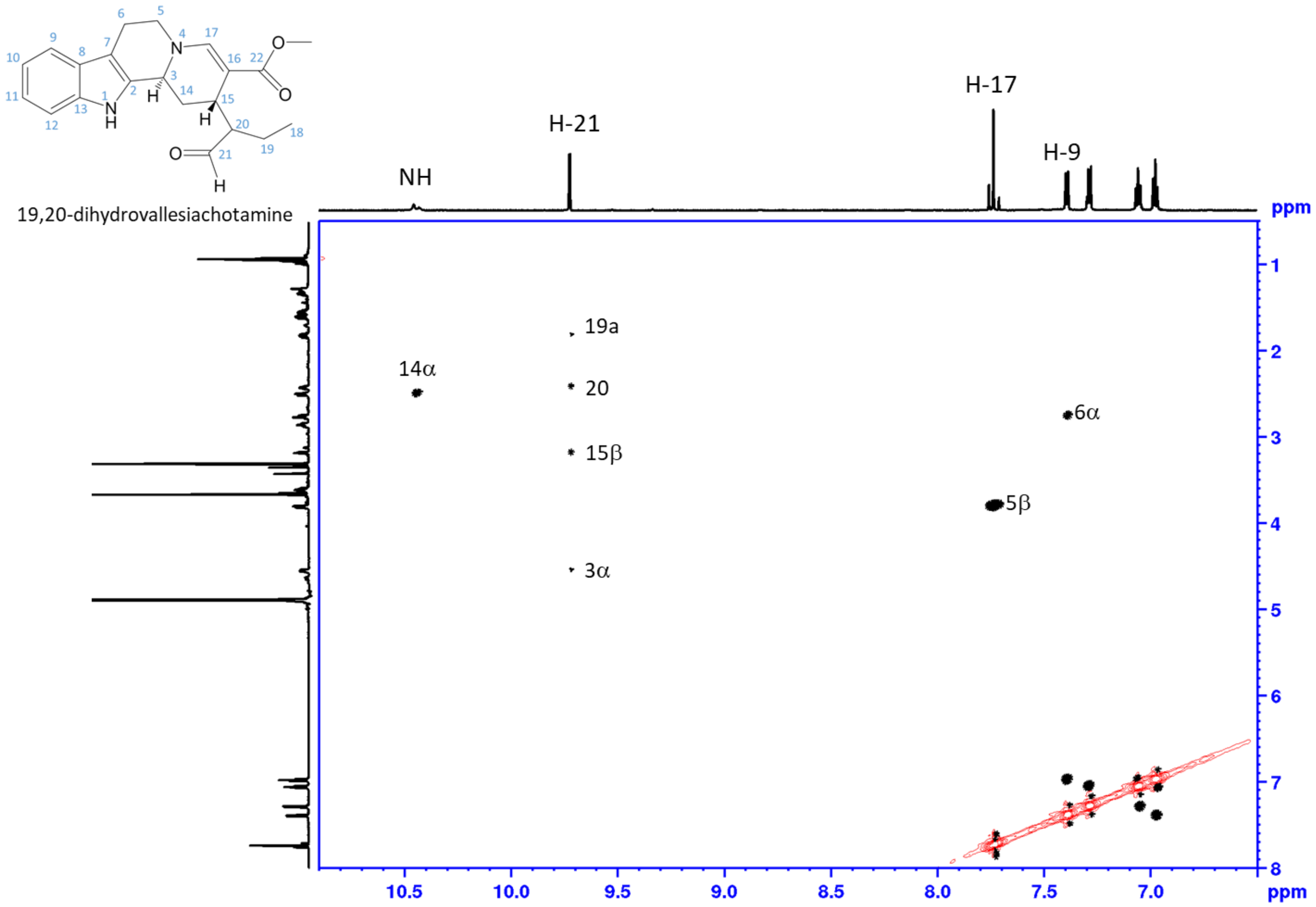

Figure S38. NMR data of 19,20-dihydrovallesiachotamine, ^{1}H-^{1}H ROESY with water suppression, aldehyde and aromatic range in MeOH-d_3

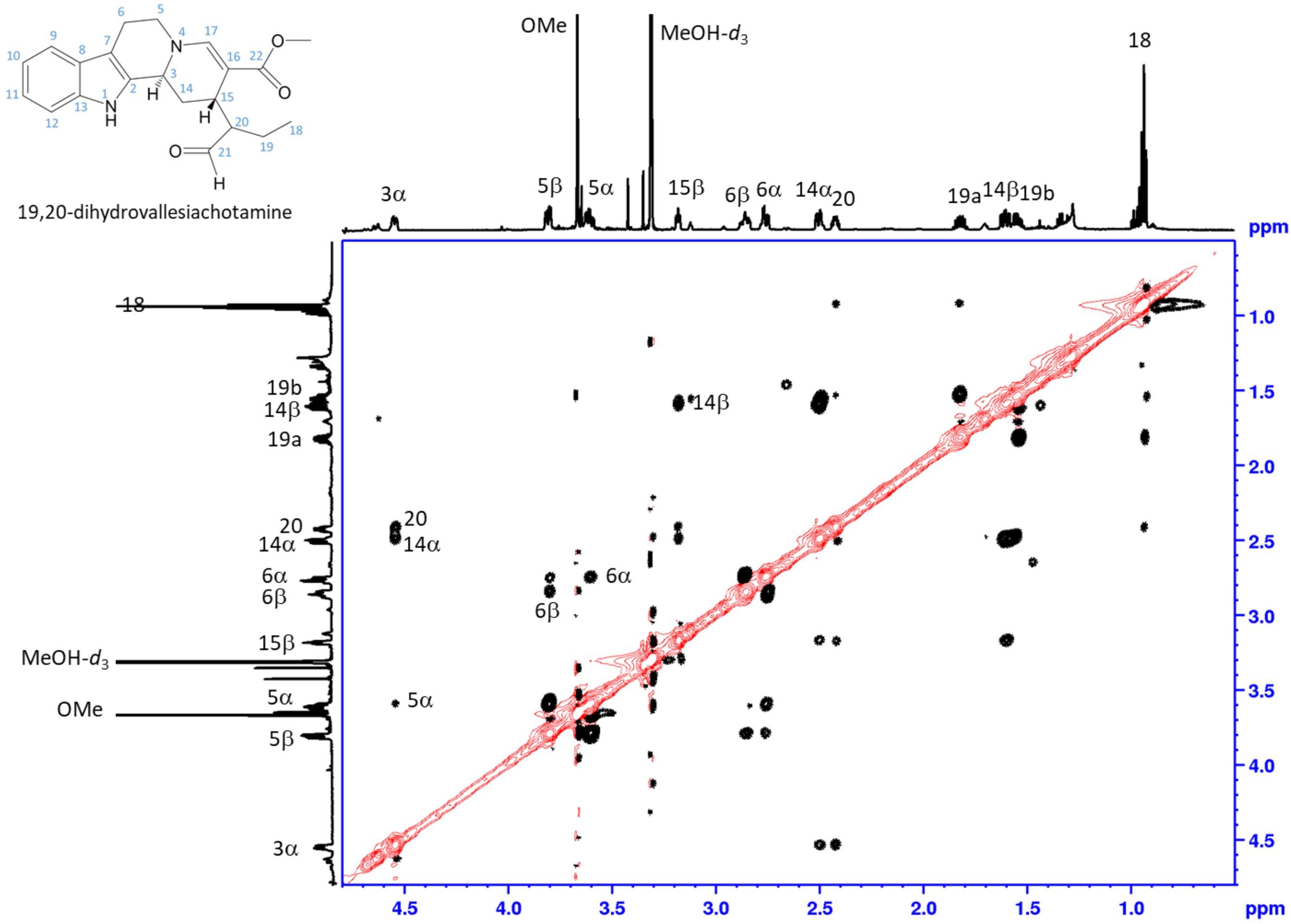

Figure S39. NMR data of 19,20-dihydrovallesiachotamine, ^{1}H-^{1}H ROESY with water suppression, aliphatic range in MeOH-d_3

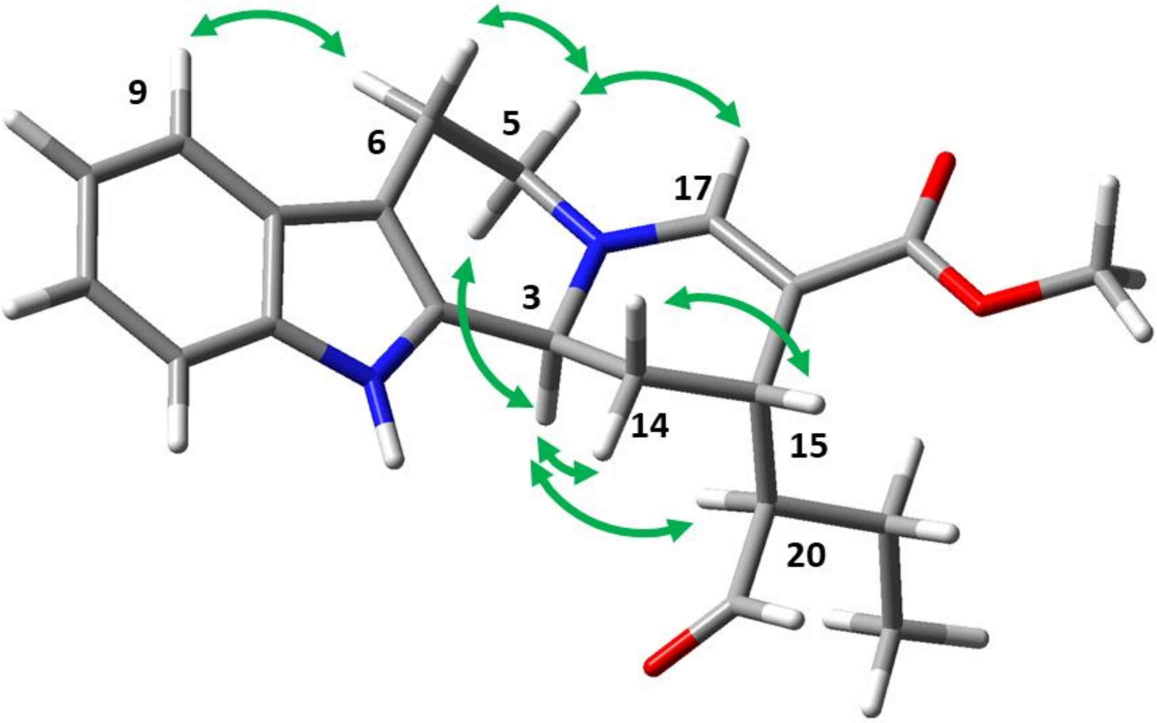

Figure S40. Structure of 19,20-dihydrovallesiachotamine optimized using Gaussian 16 (DFT APFD/6-311G++(2d,p), solvent MeOH). Important ROESY correlations extracted from NMR data are depicted in green.

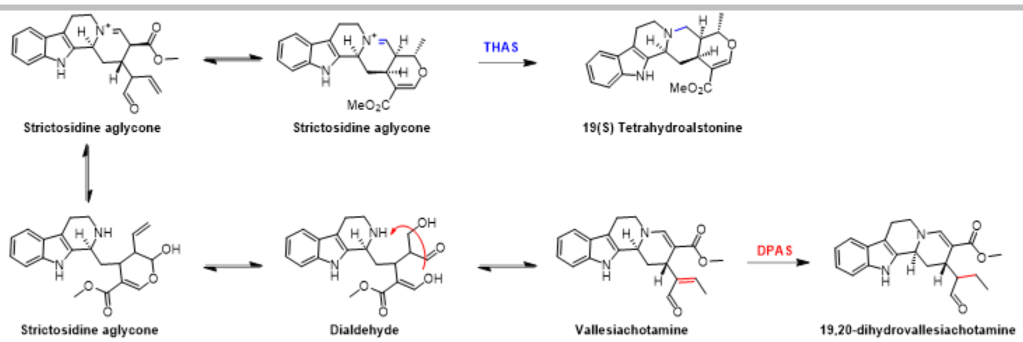

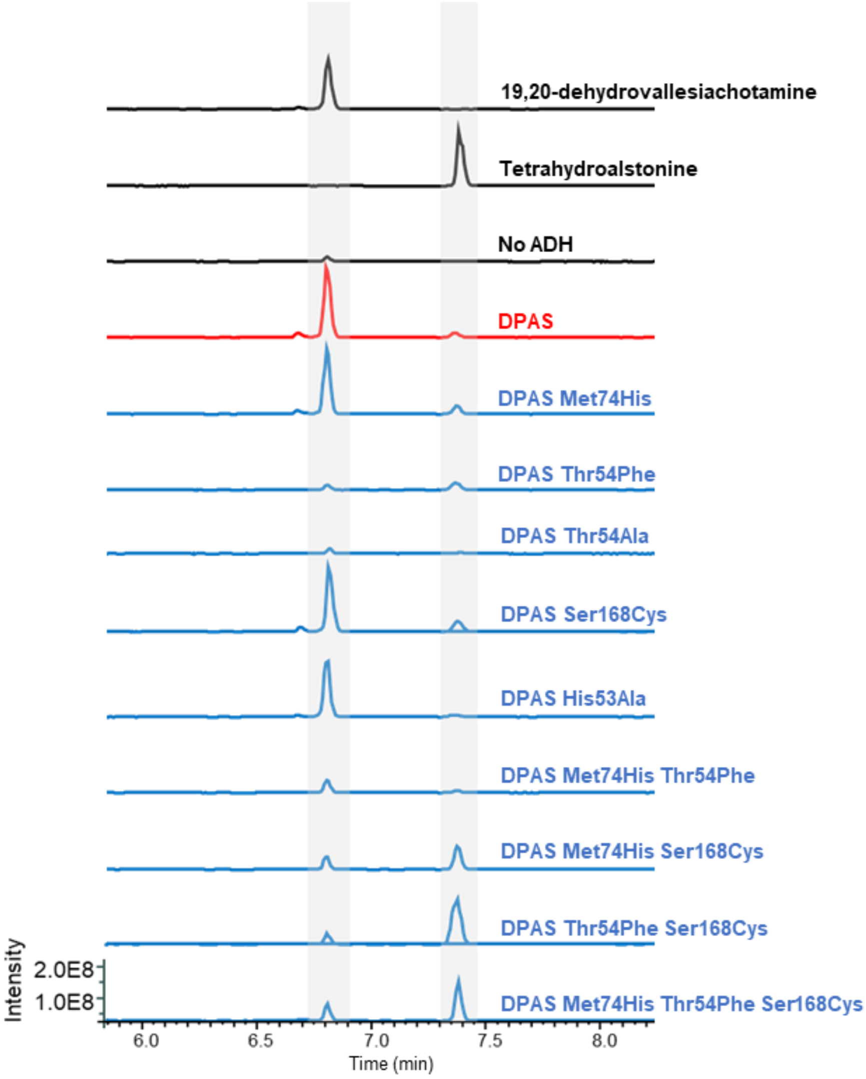

Figure S41. LC-MS chromatograms of *Cr*DPAS and mutants reacted with substrate strictosidine aglycone and cofactor NADPH. EIC *m/z* 353.185-353.225.

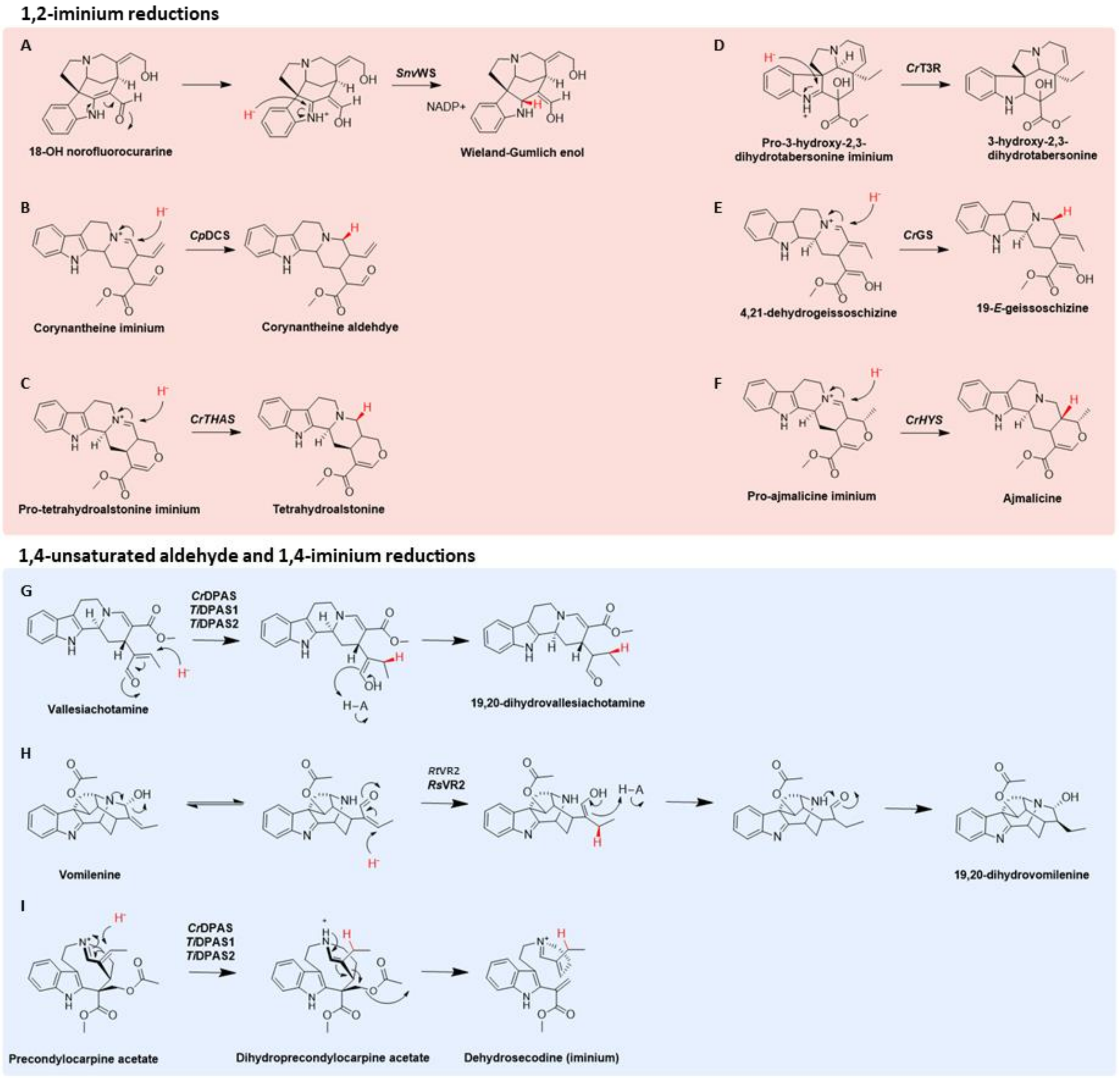

Figure S42. Previously characterised reactions in monoterpene indole alkaloid biosynthesis catalysed by ADHs. Reactions A-F are 1,2-iminium reductions, reactions G-H are 1,4 unsaturated aldehyde reductions and reaction I is 1,4-iminium reduction. **A.** *Strychnos nux-vomica* Wieland-Gumlich synthase (WS) [27]; **B.** *Chinchona pubescens* dihydrocorynantheine aldehyde synthase (DCS) [28]; **C.** *Catharanthus roseus* tetrahydroalstonine synthase (THAS) [4]; **D.** *Catharanthus roseus* tabersonine-3-reductase (T3R) [29]; **E.** *Catharanthus roseus* geissoschizine synthase (GS) [5]; **F.** *Catharanthus roseus* heteroyohimbine synthase (HYS) [30]; **G.** *Catharanthus roseus* and Tabernanthe iboga dihydroprecondylocarpine acetate synthase (DPAS), this study; **H.** *Rauwolfia serpentina* vomilenine reductase 2 (VR2) [31]; **I.** *Catharanthus roseus* and *Tabernanthe iboga* dihydroprecondylocarpine acetate synthase (DPAS) [1,2].

Supporting Tables

Table S1. Primer sequences used in this study. Cloning overhangs are underlined. Mutated codons are in bold.

**Primers for full length gene amplification**	
CrDPAS_Fwd	AAGTTCTGTTTCAGGGCCCGATGGCAGGTAAAAGCGCAGAAGAAG
CrDPAS_Rev	ATGGTCTAGAAAGCTTTACAGTTCGCTAGGCGGTGTCAG
TiDPAS1_Fwd	AAGTTCTGTTTCAGGGCCCGATGGCAGTTAAGTCACCAGAAG
TiDPAS1_Rev	ATGGTCTAGAAAGCTTTACTCAGGGGGCGTAAGGGTGTTA
TiDPAS2_Fwd	AAGTTCTGTTTCAGGGCCCGATGGCGGGCAAATCCCCCGAAG
TiDPAS2_Rev	ATGGTCTAGAAAGCTTTACGGTTCTGGAGGCGGAGTCAAAG
CrGS_Fwd	AAGTTCTGTTTCAGGGCCCGATGGCTGGTGAAACCACCAAAC
CrGS_Rev	ATGGTCTAGAAAGCTTTATTCTTCGAATTTCAGGGTGTTAC
CrTHAS_Fwd	AAGTTCTGTTTCAGGGCCCGGCAATGGCTTCAAAGTCACCTTCTG
CrTHAS_Rev	ATGGTCTAGAAAGCTTTAATTTGATTTCAGAGTGTTC

**Primers for mutagenesis**	
CrDPAS_M74H_Fwd	TATCCGCTGGTTCCTGGT**CAT**GAAATTGTTGGTATTGCAAC
CrDPAS_M74H_Rev	ACCAGGAACCAGCGGATAGCTCAG
CrDPAS_T54F_Fwd	GTATTGCGGCATTTGTCAT**TTC**GATCTGGCAAGCATTAAAAAC
CrDPAS_T54F_Rev	ATGACAAATGCCGCAATACAGAATTTTG
CrDPAS_S168C_Fwd	GGTGCTCCGCTGCTG**TGT**GCAGGTATTACCAGCTTTAG
CrDPAS_S168C_Rev	CAGCAGCGGAGCACCGCCTGC
CrDPAS_T54A_Fwd	TATTGCGGCATTTGTCAT**GCC**GATCTGGCAAGCATTAAAAAC
CrDPAS_T54A_Rev	ATGACAAATGCCGCAATACAGAATTTTG
CrDPAS_H53A_Fwd	TGTATTGCGGCATTTGT**GCT**ACCGATCTGGCAAGCATT
CrDPAS_H53A_Rev	ACAAATGCCGCAATACAGAATTTTGA
CrGS_H73M_Fwd	TACCCGTACGTTTTCGGT**ATG**GAAACCGCTGGTGAAGTTGT
CrGS_H73M_Rev	ACCGAAAACGTACGGGTAACGGGT
CrGS_F53T_Fwd	GTACTCTGGTGTTTGCAAC**ACC**GACATGGAAATGGTTCGTAAC
CrGS_F53T_Rev	GTTGCAAACACCAGAGTACAGAACACGG
CrGS_C168S_Fwd	GGTGTTGCTCTGCTG**AGC**GCTGGTGTTGTTGTTTACTC
CrGS_C168S_Rev	CAGCAGAGCAACACCTTTGTC
CrGS_F53Y_Fwd	TACTCTGGTGTTTGCAAC**TAC**GACATGGAAATGGTTCGT
CrGS_F53Y_Rev	GTTGCAAACACCAGAGTACAGAACACGG
CrTHAS_Y55F_Fwd	GTGGGACTTGCCAA**TTT**GACAGGGAAATGAG
CrTHAS_Y55F_Rev	TTGGCAAGTCCCACAGTATAATAC

SUPPORTING INFORMATION

Table S2. Full length sequences of codon optimized genes used in this study.

Codon optimized CrDPAS	ATGGCAGGTAAAAGCGCAGAAGAAGAACATCCGATTAAAGCATATGGTTGGGCAGTTAAAGATCGTACCACCGGTATTCTGAGCCCGTT<br>TAAATTCAGCCGTCGTGCAACCGGTGATGATGATGTTCGTATCAAAATTCTGTATTGCGGCATTTGTCATACCGATCTGGCAAGCATTAA<br>AAACGAATATGAGTTTCTGAGCTATCCGCTGGTTCCTGGTATGGAAATTGTTGGTATTGCAACCGAAGTTGGTAAAGATGTGACCAAAGT<br>TAAAGTGGGTGAAAAAGTTGCACTGAGCGCATATCTGGGTTGTTGTGGTAAATGTTATAGCTGCGTGAATGAGCTGGAAAACTATTGTC<br>CGGAAGTGATTATTGGTTATGGCACCCCGTATCATGATGGCACCATTTGTTATGGTGGTCTGAGCAATGAAACCGTTGCAAATCAGAGC<br>TTTGTTCTGCGTTTTCCGGAACGTCTGAGTCCGGCAGGCGGTGCTCCGCTGCTGAGCGCAGGTATTACCAGCTTTAGCGCAATGCGTA<br>ATAGCGGTATTGATAAACCGGGTCTGCATGTTGGTGTTGTTGGTTTAGGTGGTCTGGGTCATCTGGCCGTTAAATTTGCAAAAGCATTTG<br>GTCTGAAAGTGACCGTTATTAGCACCACACCGAGCAAAAAAGATGATGCAATTAATGGCCTGGGTGCAGATGGTTTTCTGCTGAGCCGT<br>GATGACGAGCAGATGAAAGCAGCAATTGGCACCCTGGATGCCATTATTGATACCCTGGCAGTTGTTCATCCGATTGCACCGCTGCTGG<br>ATCTGCTGCGTAGCCAGGGTAAATTTCTGCTGCTGGGTGCACCGAGCCAGAGCCTGGAACTGCCTCCGATTCCTCTGCTGAGTGGTGG<br>TAAAAGCATTATTGGTAGCGCAGCAGGTAATGTTAAACAGACCCAAGAAATGCTGGATTTTGCAGCCGAACATGATATTACCGCCAACG<br>TTGAAATTATCCCGATCGAATACATTAACACCGCAATGGAACGCCTGGATAAAGGTGATGTGCGTTATCGTTTTGTGGTGGATATTGAAA<br>ATACCCTGACACCGCCTAGCGAACTGTAA
Codon optimized TiDPAS2	ATGGCGGGCAAATCCCCCGAAGAAGAGCACCCGGTAAAAGCCTATGGCTGGGCAGTGAAAGACCGCACCACGGGTATTCTGTCTCCA<br>TTCAAGTTCTCACGCCGGGCAACAGGTGATAATGACATTCGCATTAAAATTCTTTACTGCGGGATTTGCCATACTGACTTGACATCAGTG<br>AAAAACGAATACGAGTTCCTTTCATACCCCTTAGTACCGGGTATGGAGATCGTTGGTATCGCTACTGAAGTGGGAAGTAAGGTTACGAA<br>AATCAAGGTTGGGGAGAAAGTGGCGGTGGCCGCGTACTTGGGGACTTGTGGTAAGTGTTATAATTGCGTGAACGATCTTGAAAACTACT<br>GTCCCGAGGTCATTATTGGATACGGTACTCCATATCACGACGGGACGATTAACTATGGAGGACTTTCGAACGAGACCGTAGTGAATGAG<br>AGATTTGTCTTGCGTTTTCCGGAAAAGCTTTCGCCGGCTGGCGGAGCTCCTCTGTTAAGCGCAGGGATTACCGCATACTCGGCTATGC<br>GTAACCATGGACTTGACAAGCCAGGTATCCATCTTGGTGTTGTCGGGCTTGGAGGACTGGGGCACCTTGCTGTTAAGTTTGCTAAAGC<br>GTTTGGCGTTCGTGTCACCGTCATCTCTACTACGCCTTCTAAAAAAGATGAGGCTATCAATAACCTGGGCGCGGACGCCTTCTTATTCA<br>GCCGGGACGATAAGCAGATGCGGGCTGCCATCGGTACCTTTGATGCCATAATCGATACGTTGGCGGTGGTCCATCCCATCGCACCATT<br>ATTAGATTTATTGCGTAGCCATGGTAAGCTGGTTCTTGTTGGCGCTCCGAGTAAGCCTTTAGAATTGCCAACAATCCCATTACTGAGCGG<br>GGGGAAGTCGCTTATAGGCTCCGCAGCAGGGAATGTCAAGCAAACTCAGGAGATGTTGGACTTTGCTGCTGAACATGATATCACCGCC<br>AACATAGAGGTAATACCTATTGACTATATCAATACAGCCATGGAACGTCTTGATAAGGGAGATATACGCTTTCGTTTCGTTGTAGACATC<br>GAAAACACTTTGACTCCGCCTCCAGAACCGTAA
Codon optimized CrGS	ATGGCTGGTGAAACCACCAAACTGGACCTGTCTGTTAAAGCTGTTGGTTGGGGTGCTGCTGACGCTTCTGGTGTTCTGCAGCCGATCA<br>AATTCTACCGTCGTGTTCCGGGTGAACGTGACGTTAAAATCCGTGTTCTGTACTCTGGTGTTTGCAACTTCGACATGGAAATGGTTCGTA<br>ACAAATGGGGTTTCACCCGTTACCCGTACGTTTTCGGTCACGAAACCGCTGGTGAAGTTGTTGAAGTTGGTTCTAAAGTTGAAAAATTCA<br>AAGTTGGTGACAAAGTTGCTGTTGGTTGCATGGTTGGTTCTTGCGGTCAGTGCTACAACTGCCAGTCTGGTATGGAAAACTACTGCCCG<br>GAACCGAACATGGCTGACGGTTCTGTTTACCGTGAACAGGGTGAACGTTCTTACGGTGGTTGCTCTAACGTTATGGTTGTTGACGAAAA<br>ATTCGTTCTGCGTTGGCCGGAAAACCTGCCGCAGGACAAAGGTGTTGCTCTGCTGTGCGCTGGTGTTGTTGTTTACTCTCCGATGAAAC<br>ACCTGGGTCTGGACAAACCGGGTAAACACATCGGTGTTTTCGGTCTGGGTGGTCTGGGTTCTGTTGCTGTTAAATTCATCAAAGCTTTC<br>GGTGGTAAAGCTACCGTTATCTCTACCTCTCGTCGTAAAGAAAAAGAAGCTATCGAAGAACACGGTGCTGACGCTTTCGTTGTTAACAC<br>CGACTCTGAACAGCTGAAAGCTCTGGCTGGTACCATGGACGGTGTTGTTGACACCACCCCGGGTGGTCGTACCCCGATGTCTCTGATG<br>CTGAACCTGCTGAAATTCGACGGTGCTGTTATGCTGGTTGGTGCTCCGGAATCTCTGTTCGAACTGCCGGCTGCTCCGCTGATCATGG<br>GTCGTAAAAAAATCATCGGTTCTTCTACCGGTGGTCTGAAAGAATACCAGGAAATGCTGGACTTCGCTGCTAAACACAACATCGTTTGC<br>GACACCGAAGTTATCGGTATCGACTACCTGTCTACCGCTATGGAACGTATCAAAAACCTGGACGTTAAATACCGTTTCGCTATCGACATC<br>GGTAACACCCTGAAATTCGAAGAATAA
Codon optimized CrTHAS	ATGGCAATGGCTTCAAAGTCACCTTCTGAAGAAGTATATCCAGTGAAGGCATTTGGTTTGGCTGCTAAGGATTCTTCTGGGCTTTTCTCT<br>CCATTCAACTTCTCAAGAAGGGCCACAGGGGAACACGATGTGCAGCTCAAAGTATTATACTGTGGGACTTGCCAATATGACAGGGAAAT<br>GAGCAAAAACAAATTTGGATTTACAAGCTATCCTTATGTTTTAGGGCATGAAATTGTGGGTGAGGTAACTGAAGTTGGCAGCAAGGTGCA<br>GAAATTCAAAGTCGGGGACAAAGTGGGCGTAGCAAGCATAATTGAAACTTGTGGCAAATGTGAAATGTGTACAAATGAAGTTGAAAATTA<br>CTGTCCAGAAGCAGGATCAATAGACAGCAATTACGGGGCATGTTCAAATATAGCAGTGATAAACGAGAATTTTGTCATCCGTTGGCCTG<br>AAAATCTTCCTTTGGATTCTGGTGTTCCTCTTCTATGTGCAGGAATCACGGCTTATAGTCCCATGAAACGTTATGGACTTGATAAACCTG<br>GAAAACGTATCGGCATAGCCGGTCTAGGAGGACTTGGACATGTAGCTCTTAGATTTGCCAAAGCTTTTGGGGCTAAGGTGACAGTGATT<br>AGTTCTTCACTTAAGAAAAAACGTGAAGCCTTTGAGAAATTCGGAGCAGATTCTTTCTTGGTCAGCAGTAATCCAGAAGAAATGCAGGGT<br>GCAGCAGGAACATTGGATGGGATCATAGACACTATACCAGGGAATCACTCTCTTGAGCCACTCCTTGCTTTATTGAAGCCTCTTGGGAA<br>GCTTATCATTTTAGGTGCACCAGAAATGCCCTTTGAGGTTCCCGCTCCTTCCCTGCTTATGGGTGGAAAAGTAATGGCTGCCAGTACTG<br>CTGGGAGTATGAAGGAAATACAAGAGATGATTGAATTTGCAGCAGAACACAACATAGTAGCAGATGTGGAGGTTATCTCTATTGACTATG<br>TGAACACTGCAATGGAGCGCCTTGATAACTCTGATGTGAGATATCGTTTCGTGATTGATATAGGGAACACTCTGAAATCAAATTAA
*Tb*ADH	AAGTTCTGTTTCAGGGCCCGAAAGGTTTTGCAATGCTCAGTATCGGTAAAGTTGGCTGGATTGAGAAGGAAAAGCCTGCTCCTGGCCCA<br>TTTGATGCTATTGTAAGACCTCTAGCTGTGGCCCCTTGCACTTCGGACATTCATACCGTTTTTGAAGGCGCCATTGGCGAAAGACATAAC<br>ATGATACTCGGTCACGAAGCTGTAGGTGAAGTAGTTGAAGTAGGTAGTGAGGTAAAAGATTTTAAACCTGGTGATCGCGTTGTTGTGCC<br>AGCTATTACCCCTGATTGGCGGACCTCTGAAGTACAAAGAGGATATCACCAGCACTCCGGTGGAATGCTGGCAGGCTGGAAATTTTCG<br>AATGTAAAAGATGGTGTTTTTGGTGAATTTTTTCATGTGAATGATGCTGATATGAATTTAGCACATCTGCCTAAAGAAATTCCATTGGAAG<br>CTGCAGTTATGATTCCCGATATGATGACCACTGGTTTTCACGGAGCTGAACTGGCAGATATAGAATTAGGTGCGACGGTAGCAGTTTTG<br>GGTATTGGCCCAGTAGGTCTTATGGCAGTCGCTGGTGCCAAATTGCGTGGAGCCGGAAGAATTATTGCCGTAGGCAGTAGACCAGTTT<br>GTGTAGATGCTGCAAAATACTATGGAGCTACTGATATTGTAAACTATAAAGATGGTCCTATCGAAAGTCAGATTATGAATCTAACTGAAG<br>GCAAAGGTGTCGATGCTGCCATCATCGCTGGAGGAAATGCTGACATTATGGCTACAGCAGTTAAGATTGTTAAACCTGGTGGCACCATC<br>GCTAATGTAAATTATTTTGGCGAAGGAGAGGTTTTGCCTGTTCCTCGTCTTGAATGGGGTTGCGGCATGGCTCATAAAACTATAAAAGGC<br>GGGCTATGCCCCGGTGGACGTCTAAGAATGGAAAGACTGATTGACCTTGTTTTTTATAAGCGTGTCGATCCTTCTAAGCTCGTCACTCA<br>CGTTTTCCGGGGATTTGACAATATTGAAAAAGCCTTTATGTTGATGAAAGACAAACCAAAAGACCTAATCAAACCTGTTGTAATATTAGCA<br>TAAAGCTTTCTAGACCAT

Table S3. Summary of X-ray data and model parameters for *Cr*DPAS

Data collection	
Paul Scherrer Institute	10SA (PX II)
Wavelength (Å)	1
Resolution range (Å)	44.62 - 2.45 (2.548 - 2.45)
Space Group	*P 21 21 21*
Cell parameters (Å)	*a* = 61.019, *b* = 114.015, *c* = 143.357, β = 90°
Total no. of measured reflections	494135 (51201)
Unique reflections	37564 (3719)
Multiplicity	13.2 (13.8)
Mean $I/\sigma(I)$	21.46 (3.10)
Completeness (%)	98.7 (96.8)
R_{merge}[a]	0.2154 (1.406)
R_{meas}[b]	0.2242 (1.46)
$CC_{½}$[c]	0.999 (0.879)
Wilson *B* value ($Å^2$)	53.18
Refinement	
Reflections used in refinement	37560 (3719)
Reflections used for R-free	1877 (186)
R_{work}	0.2217 (0.2772)
R_{free}	0.2501 (0.3143)
CC_{work}	0.942 (0.845)
CC_{free}	0.958 (0.704)
Protein residues	640
Number of non-hydrogen atoms	4668
macromolecules	4566
ligands	43
solvent	69
Ramachandran plot: favoured/allowed/disallowed[f] (%)	98.1/1.58/0.32
Rotamer outliers (%)	3.97
R.m.s. bond distance deviation (Å)	0.007
R.m.s. bond angle deviation (º)	0.98
Clashscore	19.95
Mean *B* factors: protein/waters/ligands/overall ($Å^2$)	62.93/54.82/76.91/62.91
PDB accession code	8B27

Statistics for the highest-resolution shell are shown in parentheses.
[a] $R_{merge} = \sum_{hkl} \sum_i |I_i(hkl) - \langle I(hkl)\rangle| / \sum_{hkl} \sum_i I_i(hkl)$.
[b] $R_{meas} = \sum_{hkl} [N/(N-1)]^{1/2} \times \sum_i |I_i(hkl) - \langle I(hkl)\rangle| / \sum_{hkl} \sum_i I_i(hkl)$, where $I_i(hkl)$ is the *i*th observation of reflection *hkl*, $\langle I(hkl)\rangle$ is the weighted average intensity for all observations *i* of reflection *hkl* and *N* is the number of observations of reflection *hkl*.
[c] $CC_{½}$ is the correlation coefficient between symmetry equivalent intensities from random halves of the dataset.
[d] The data set was split into "working" and "free" sets consisting of 95 and 5% of the data respectively. The free set was not used for refinement.
[e] The R-factors R_{work} and R_{free} are calculated as follows: $R = \sum(|F_{obs} - F_{calc}|)/\sum|F_{obs}|$, where F_{obs} and F_{calc} are the observed and calculated structure factor amplitudes, respectively.
[f] As calculated using MolProbity [32].

Table S4. Summary of X-ray data and model parameters for apo-*Ti*DPAS2

Data collection	
Paul Scherrer Institute	10SA (PX II)
Wavelength (Å)	1
Resolution range (Å)	41.64 - 2.421 (2.508 - 2.421)
Space Group	*P 21 21 21*
Cell parameters (Å)	*a* = 74.422, *b* = 78.124, *c* = 131.207, β = 90°
Total no. of measured reflections	341639 (17075)
Unique reflections	29562 (2702)
Multiplicity	11.6 (6.3)
Mean $I/\sigma(I)$	16.49 (1.32)
Completeness (%)	98.98 (91.90)
$R_{merge}{}^{a}$	0.08578 (0.8446)
$R_{meas}{}^{b}$	0.0897 (0.9206)
$CC_{½}{}^{c}$	0.999 (0.785)
Wilson *B* value ($Å^2$)	64.90
Refinement	
Reflections used in refinement	29531 (2700)
Reflections used for R-free	1477 (135)
R_{work}	0.2082 (0.3777)
R_{free}	0.2552 (0.4209)
CC_{work}	0.965 (0.812)
CC_{free}	0.901 (0.716)
Protein residues	716
Number of non-hydrogen atoms	5305
macromolecules	5269
ligands	2
solvent	34
Ramachandran plot: favoured/allowed/disallowed[f] (%)	98.87/1.13/0.00
Rotamer outliers (%)	2.43
R.m.s. bond distance deviation (Å)	0.006
R.m.s. bond angle deviation (º)	0.95
Clashscore	5.27
Mean *B* factors: protein/waters/ligands/overall ($Å^2$)	71.16/57.66/62.53/71.07
PDB accession code	8B26

Statistics for the highest-resolution shell are shown in parentheses.
[a] $R_{merge} = \sum_{hkl} \sum_i |I_i(hkl) - \langle I(hkl)\rangle| / \sum_{hkl} \sum_i I_i(hkl)$.
[b] $R_{meas} = \sum_{hkl} [N/(N-1)]^{1/2} \times \sum_i |I_i(hkl) - \langle I(hkl)\rangle| / \sum_{hkl} \sum_i I_i(hkl)$, where $I_i(hkl)$ is the *i*th observation of reflection *hkl*, $\langle I(hkl)\rangle$ is the weighted average intensity for all observations *i* of reflection *hkl* and *N* is the number of observations of reflection *hkl*.
[c] $CC_{½}$ is the correlation coefficient between symmetry equivalent intensities from random halves of the dataset.
[d] The data set was split into "working" and "free" sets consisting of 95 and 5% of the data respectively. The free set was not used for refinement.
[e] The R-factors R_{work} and R_{free} are calculated as follows: $R = \sum(|F_{obs} - F_{calc}|)/\sum|F_{obs}|$, where F_{obs} and F_{calc} are the observed and calculated structure factor amplitudes, respectively.
[f] As calculated using MolProbity [32].

Table S5. Summary of X-ray data and model parameters for precondylocarpine acetate-bound *Ti*DPAS2

Data collection	
Paul Scherrer Institute	10SA (PX II)
Wavelength (Å)	1
Resolution range (Å)	39.81 – 1.882 (1.949 – 1.882)
Space Group	*P 21 21 21*
Cell parameters (Å)	*a* = 72.888, *b* = 79.624, *c* = 130.801, β = 90°
Total no. of measured reflections	809479 (78567)
Unique reflections	62174 (5895)
Multiplicity	13.0 (13.3)
Mean $I/\sigma(I)$	14.05 (0.88)
Completeness (%)	99.49 (95.74)
R_{merge} [a]	0.1082 (3.23)
R_{meas} [b]	0.1128 (3.357)
$CC_{½}$ [c]	0.999 (0.463)
Wilson *B* value (Å^2)	40.94
Refinement	
Reflections used in refinement	62152 (5895)
Reflections used for R-free	3104 (295)
R_{work}	0.1927 (0.4735)
R_{free}	0.2216 (0.5240)
CC_{work}	0.972 (0.696)
CC_{free}	0.966 (0.671)
Protein residues	716
Number of non-hydrogen atoms	5601
macromolecules	5272
ligands	97
solvent	242
Ramachandran plot: favoured/allowed/disallowed[f] (%)	97.33/2.67/0.00
Rotamer outliers (%)	0.93
R.m.s. bond distance deviation (Å)	0.004
R.m.s. bond angle deviation (º)	0.71
Clashscore	3.89
Mean *B* factors: protein/waters/ligands/overall (Å^2)	44.88/47.01/46.74/45.00
PDB accession code	8B1V

Statistics for the highest-resolution shell are shown in parentheses.

[a] $R_{merge} = \sum_{hkl} \sum_i |I_i(hkl) - \langle I(hkl)\rangle| / \sum_{hkl} \sum_i I_i(hkl)$.

[b] $R_{meas} = \sum_{hkl} [N/(N-1)]^{1/2} \times \sum_i |I_i(hkl) - \langle I(hkl)\rangle| / \sum_{hkl} \sum_i I_i(hkl)$, where $I_i(hkl)$ is the *i*th observation of reflection *hkl*, $\langle I(hkl)\rangle$ is the weighted average intensity for all observations *i* of reflection *hkl* and *N* is the number of observations of reflection *hkl*.

[c] $CC_{½}$ is the correlation coefficient between symmetry equivalent intensities from random halves of the dataset.

[d] The data set was split into "working" and "free" sets consisting of 95 and 5% of the data respectively. The free set was not used for refinement.

[e] The R-factors R_{work} and R_{free} are calculated as follows: $R = \sum(|F_{obs} - F_{calc}|)/\sum|F_{obs}|$, where F_{obs} and F_{calc} are the observed and calculated structure factor amplitudes, respectively.

[f] As calculated using MolProbity [32].

Table S6. Summary of X-ray data and model parameters for stemmadenine acetate-bound *Ti*DPAS2

Data collection	
Paul Scherrer Institute	10SA (PX II)
Wavelength (Å)	1
Resolution range (Å)	39.92 – 2.24 (2.32 – 2.24)
Space Group	*P 21 21 21*
Cell parameters (Å)	a = 73.186, b = 79.845, c = 130.922, β = 90°
Total no. of measured reflections	432608 (21387)
Unique reflections	35719 (2561)
Multiplicity	12.1 (8.4)
Mean $I/\sigma(I)$	17.69 (1.77)
Completeness (%)	94.79 (68.96)
R_{merge}^{a}	0.1239 (1.273)
R_{meas}^{b}	0.1294 (1.358)
$CC_{½}^{c}$	0.999 (0.586)
Wilson B value (Å^2)	44.54
Refinement	
Reflections used in refinement	35691 (2561)
Reflections used for R-free	1786 (128)
R_{work}	0.1737 (0.3245)
R_{free}	0.2199 (0.3957)
CC_{work}	0.972 (0.790)
CC_{free}	0.957 (0.700)
Protein residues	717
Number of non-hydrogen atoms	5530
macromolecules	5272
ligands	114
solvent	168
Ramachandran plot: favoured/allowed/disallowed[f] (%)	96.49/3.51/0.00
Rotamer outliers (%)	2.79
R.m.s. bond distance deviation (Å)	0.148
R.m.s. bond angle deviation (º)	4.02
Clashscore	5.86
Mean B factors: protein/waters/ligands/overall (Å^2)	45.79/46.44/45.13/45.78
PDB accession code	8B25

Statistics for the highest-resolution shell are shown in parentheses.
[a] $R_{merge} = \sum_{hkl} \sum_i |I_i(hkl) - \langle I(hkl)\rangle| / \sum_{hkl} \sum_i I_i(hkl)$.
[b] $R_{meas} = \sum_{hkl} [N/(N-1)]^{1/2} \times \sum_i |I_i(hkl) - \langle I(hkl)\rangle| / \sum_{hkl} \sum_i I_i(hkl)$, where $I_i(hkl)$ is the ith observation of reflection hkl, $\langle I(hkl)\rangle$ is the weighted average intensity for all observations i of reflection hkl and N is the number of observations of reflection hkl.
[c] $CC_{½}$ is the correlation coefficient between symmetry equivalent intensities from random halves of the dataset.
[d] The data set was split into "working" and "free" sets consisting of 95 and 5% of the data respectively. The free set was not used for refinement.
[e] The R-factors R_{work} and R_{free} are calculated as follows: $R = \sum(| F_{obs} - F_{calc} |)/\sum| F_{obs} |$, where F_{obs} and F_{calc} are the observed and calculated structure factor amplitudes, respectively.
[f] As calculated using MolProbity [32].

Table S7. Summary of X-ray data and model parameters for *Cr*GS

Data collection	
Diamond Light Source beamline	I03
Wavelength (Å)	0.9762
Detector	Pilatus3 6M
Resolution range (Å)	63.84 – 2.00 (2.05 – 2.0)
Space Group	$P2_1$
Cell parameters (Å)	a = 55.4, b = 101.0, c = 66.7, β = 106.8°
Total no. of measured intensities	299680 (22122)
Unique reflections	46753 (3406)
Multiplicity	6.4 (6.5)
Mean $I/\sigma(I)$	9.8 (3.2)
Completeness (%)	98.7 (96.8)
R_{merge}[a]	0.150 (0.740)
R_{meas}[b]	0.180 (0.886)
$CC_{½}$[c]	0.986 (0.648)
Wilson B value ($Å^2$)	19.2
Refinement	
Resolution range (Å)	63.84 – 2.00 (2.05 – 2.00)
Reflections: working/free[d]	44395/2334
R_{work}/ R_{free}[e]	0.205/0.232 (0.401/0.413)
Ramachandran plot: favoured/allowed/disallowed[f] (%)	97.1/2.8/0.1
R.m.s. bond distance deviation (Å)	0.004
R.m.s. bond angle deviation (º)	1.65
No. of protein residues: (ranges)	A:351 (9-129, 132-361)<br>B:352 (8-129, 134-363)
No. of water/Zinc/NAP molecules	279/4/2
Mean B factors: protein/waters/ligands/overall ($Å^2$)	15/33/24
PDB accession code	8A3N

Values in parentheses are for the outer resolution shell.

[a] $R_{merge} = \sum_{hkl} \sum_i |I_i(hkl) - \langle I(hkl)\rangle| / \sum_{hkl} \sum_i I_i(hkl)$.

[b] $R_{meas} = \sum_{hkl} [N/(N-1)]^{1/2} \times \sum_i |I_i(hkl) - \langle I(hkl)\rangle| / \sum_{hkl} \sum_i I_i(hkl)$, where $I_i(hkl)$ is the ith observation of reflection hkl, $\langle I(hkl)\rangle$ is the weighted average intensity for all observations i of reflection hkl and N is the number of observations of reflection hkl.

[c] $CC_{½}$ is the correlation coefficient between symmetry equivalent intensities from random halves of the dataset.

[d] The data set was split into "working" and "free" sets consisting of 95 and 5% of the data respectively. The free set was not used for refinement.

[e] The R-factors R_{work} and R_{free} are calculated as follows: $R = \sum(|F_{obs} - F_{calc}|)/\sum|F_{obs}|$, where F_{obs} and F_{calc} are the observed and calculated structure factor amplitudes, respectively.

[f] As calculated using MolProbity [32].

Table S8. Genbank accession for sequences used to construct tree of maximum likelihood in Figure 4A.

Gene Name	Genbank accession
Arabidopsis thaliana cinnamyl alcohol dehydrogenase 5 (CAD5)	NM_119587.4
Populus tremuloides sinapyl alcohol dehydrogenase (SAD)	AF273256.1
Camptotheca accuminata 8-hydroxygeraniol oxidase (8HGO)	AY342355.1
Ocimum basilcum geraniol dehydrogenase (GEDH)	AY879284.1
Rauwolfia serpentina cinnamyl alcohol dehydrogenase (CAD)	KT369739.1
Catharanthus roseus 8-hydrogeraniol dehydrogenas (8HGO)	KF561458.1
Strychnos speciosa Wieland-Gumlich aldehyde synthase (WS)	OM304303.1
Strychnos nux-vomica Wieland-Gumlich aldehyde synthase (WS)	OM304294.1
Catharanthus roseus Geissoschizine synthase (GS)	MF770507.1
Cinchona pubescens dihydrocorinantheine aldehyde synthase (DCS)	MW456554
Catharanthus roseus tabersonine 3- reductase (T3R)	KP122966.1
Catharanthus roseus tetrahydroalstonine synthase (THAS)	KM524258.1
Catharanthus roseus heteroyohimbine synthase (HYS)	KU865325.1
Rauwolfia serpentina vomilenine reductase 2 (VR2)	KT369740.1
Rauwolfia tetraphylla vomilenine reductase 2 (VR2)	KT369741.1
Tabernanthe iboga dihydroprecondylocarpine acetate synthase 1 (DPAS1)	MK840855.1
Tabernanthe iboga dihydroprecondylocarpine acetate synthase 2 (DPAS2)	MK840856.1
Catharanthus roseus dihydroprecondylocarpine acetate synthase 1 (DPAS)	KU865331.1

References

[1] L. Caputi, J. Franke, S. C. Farrow, K. Chung, R. M. E. Payne, T.-D. Nguyen, T.-T. T. Dang, I. S. T. Carqueijeiro, K. Koudounas, T. D. de Bernonville, B. Ameyaw, D. M. Jones, I. J. C. Vieira, V. Courdavault, S. E. O'Connor, *Science* **2018**, *360*, 1235–1239.
[2] S. C. Farrow, M. O. Kamileen, L. Caputi, K. Bussey, J. E. A. Mundy, R. C. McAtee, C. R. J. Stephenson, S. E. O'Connor, *J. Am. Chem. Soc.* **2019**, *141*, 12979–12983.
[3] M. Jarret, V. Turpin, A. Tap, J. Gallard, C. Kouklovsky, E. Poupon, G. Vincent, L. Evanno, *Angewandte Chemie Int Ed* **2019**, *58*, 9861–9865.
[4] A. Stavrinides, E. C. Tatsis, E. Foureau, L. Caputi, F. Kellner, V. Courdavault, S. E. O'Connor, *Chem. Biol.* **2015**, *22*, 336–41.
[5] Y. Qu, M. E. A. M. Easson, R. Simionescu, J. Hajicek, A. M. K. Thamm, V. Salim, V. D. Luca, *Proc. Natl. Acad. Sci. U S A* **2018**, *115*, 3180–3185.
[6] L. Caputi, J. Franke, K. Bussey, S. C. Farrow, I. J. C. Vieira, C. E. M. Stevenson, D. M. Lawson, S. E. O'Connor, *Nat. Chem. Biol.* **2020**, *16*, 383–386.
[7] N. S. Berrow, D. Alderton, S. Sainsbury, J. Nettleship, R. Assenberg, N. Rahman, D. I. Stuart, R. J. Owens, *Nucleic Acids Res.* **2007**, *35*, e45–e45.
[8] S. S. Jeong, J. E. Gready, *Anal. Biochem.* **1994**, *221*, 273–277.
[9] T. Bruhn, A. Schaumlöffel, Y. Hemberger, G. Bringmann, *Chirality* **2013**, *25*, 243–249.
[10] D. Liebschner, P. V. Afonine, M. L. Baker, G. Bunkóczi, V. B. Chen, T. I. Croll, B. Hintze, L.-W. Hung, S. Jain, A. J. McCoy, N. W. Moriarty, R. D. Oeffner, B. K. Poon, M. G. Prisant, R. J. Read, J. S. Richardson, D. C. Richardson, M. D. Sammito, O. V. Sobolev, D. H. Stockwell, T. C. Terwilliger, A. G. Urzhumtsev, L. L. Videau, C. J. Williams, P. D. Adams, *Acta Crystallogr. Sect. D* **2019**, *75*, 861–877.
[11] P. Emsley, B. Lohkamp, W. G. Scott, K. Cowtan, *Acta Crystallogr. Sect D Biological Crystallogr.* **2010**, *66*, 486–501.
[12] W. Kabsch, *Acta Crystallogr. Sect D Biological Crystallogr.* **2010**, *66*, 125–132.
[13] G. Winter, *J. Appl. Crystallogr.* **2010**, *43*, 186–190.
[14] P. R. Evans, G. N. Murshudov, *Acta Crystallogr. Sect D Biological Crystallogr.* **2013**, *69*, 1204–1214.
[15] N. Stein, *J. Appl. Crystallogr.* **2008**, *41*, 641–643.
[16] A. J. McCoy, R. W. Grosse-Kunstleve, P. D. Adams, M. D. Winn, L. C. Storoni, R. J. Read, *J. Appl. Crystallogr.* **2007**, *40*, 658–674.
[17] G. N. Murshudov, P. Skubák, A. A. Lebedev, N. S. Pannu, R. A. Steiner, R. A. Nicholls, M. D. Winn, F. Long, A. A. Vagin, *Acta Crystallogr. Sect. D Biological Crystallogr.* **2011**, *67*, 355–367.
[18] K. Cowtan, *Acta Crystallogr. Sect. D Biological Crystallogr.* **2006**, *62*, 1002–1011.
[19] O. Trott, A. J. Olson, *J. Comput. Chem.* **2010**, *31*, 455–461.
[20] Y. Kochnev, E. Hellemann, K. C. Cassidy, J. D. Durrant, *Bioinformatics* **2020**, *36*, btaa579-.
[21] W. Tian, C. Chen, X. Lei, J. Zhao, J. Liang, *Nucleic Acids Res.* **2018**, *46*, gky473-.
[22] R. C. Edgar, *Biorxiv* **2021**, 2021.06.20.449169.
[23] J. Trifinopoulos, L.-T. Nguyen, A. von Haeseler, B. Q. Minh, *Nucleic Acids Res.* **2016**, *44*, W232–W235.
[24] I. Letunic, P. Bork, *Nucleic Acids Res.* **2021**, *49*, W293-W296.
[25] S. Zhao, R. B. Andrade, *J. Org. Chem.* **2017**, *82*, 521–531.
[26] M. E. Kuehne, U. K. Bandarage, A. Hammach, Y.-L. Li, T. Wang, *J. Org. Chem.* **1998**, *63*, 2172–2183.
[27] B. Hong, D. Grzech, L. Caputi, P. Sonawane, C. E. R. López, M. O. Kamileen, N. J. H. Lozada, V. Grabe, S. E. O'Connor, *Nature* **2022**, 1–6.
[28] F. Trenti, K. Yamamoto, B. Hong, C. Paetz, Y. Nakamura, S. E. O'Connor, *Org. Lett.* **2021**, *23*, 1793–1797.
[29] Y. Qu, M. L. A. E. Easson, J. Froese, R. Simionescu, T. Hudlicky, V. DeLuca, *Proc. Natl. Acad. Sci. U S A* **2015**, *112*, 6224–6229.
[30] A. Stavrinides, E. C. Tatsis, L. Caputi, E. Foureau, C. E. M. Stevenson, D. M. Lawson, V. Courdavault, S. E. O'Connor, *Nat. Commun.* **2016**, *7*, 12116.
[31] M. Geissler, M. Burghard, J. Volk, A. Staniek, H. Warzecha, *Planta* **2016**, *243*, 813–824.
[32] I. W. Davis, A. Leaver-Fay, V. B. Chen, J. N. Block, G. J. Kapral, X. Wang, L. W. Murray, W. B. Arendall, J. Snoeyink, J. S. Richardson, D. C. Richardson, *Nucleic Acids Res.* **2007**, *35*, W375–W383.
